# Supplementary material for: Measles vaccines and non-specific effects on mortality or morbidity: A systematic review and meta-analysis
Source: PLoS One. 2025 Jul 2;20(7):e0321982. doi: 10.1371/journal.pone.0321982 (PMC12221017; doi:10.1371/journal.pone.0321982)
Supplement: S1 Appendix — (DOCX) [file pone.0321982.s001.docx]

# **S1 appendix. Supplementary methods**

# **Abbreviations:**

CI: Confidence interval

EZ-ST = Edmonston-Zagreb standard titre

EZ-HT = Edmonston-Zagreb high titre

HR = Hazard ratio

IPV = Inactivated polio vaccine
ITT = Intention to treat

MCV = Measles containing vaccine

MRR = Mortality rate ratio

MUAC = Mid-upper-arm-circumference

OIS = Optimal information size

OPV = Oral polio vaccine

PP = Per protocol

RR = Risk ratio

RRR = Relative risk reduction

SW-std/SW-ST/SW-medium = Schwartz standard titre

# **Methods**

## **Inclusion and exclusion criteria**

Criteria were determined based on the population, intervention, comparator, outcome (PICO) framework:

- P: The population was required to be humans < 18 years of age with no chronic illnesses.
- I: The intervention was required to be STMV or HTMV.
- C: The comparator was required to be other vaccines, other vaccine sequences, or placebo.
- O: Primary outcomes were all-cause mortality (excluding measles deaths) and all-cause consultations or admissions (excluding measles infections). The following off-target outcomes were included: infections excluding measles, antibiotic use, atopy, allergies, asthma, and atopic dermatitis.

Trials from both high- and low-income settings were included. To identify trials investigating clinically relevant non-specific effects of MCV, trials on immunogenicity and adverse events (within 30 days), and trials with para-clinical outcomes were excluded. Trials giving the same measles vaccine (same dose, same number of vaccines) to both the intervention and control groups were excluded.

**Systematic literature search**
A systematic search was developed in February 2022. The search was computerised and performed on EMBASE and PubMed. The search was repeated weekly from February 2022 to October 2022 and confirmed in August 2023. The search string can be found in Table in S1 Table along with raw text that can be used to reproduce the exact same search. The following languages were accepted: Danish, Swedish, Norwegian, and English. The vast majority (> 88%) of the articles relevant for the present review were presented in English. Article languages were chosen to ensure that all authors were able to understand the text without translational assistance. The language criteria excluded 526 articles. Trials with non-human populations (n=512) were excluded. Excluded articles can be found in S1 file. Reference lists of all included articles were examined to identify potential articles not found in the database searches. An ongoing trial was identified and thus not included[1].

**Screening**
EndNote 20 and Covidence were used to manage the identified articles. Covidence was used for the screening process. Two authors independently screened all abstracts in accordance with the PICO criteria and discrepancies were solved by consulting a third author. The three authors independently screened all full text articles. Disagreements were solved by discussion in the group or with the senior researcher.

## **Quality assessment and data extraction**

Three authors read all included trials and performed independent quality assessment and data extraction. Disagreements were discussed in the group. The quality assessment was based on the ROB-2 tool as recommended by the Cochrane Handbook for evaluating RCTs included in a systematic review[2]. The template ROB-2 includes evaluation of:

*Randomisation process, deviations from intended interventions, blinding of participants and personnel, measurement of the outcome, missing outcome data, selection of the reported results, and other sources of bias*[2].

Each paper was evaluated as low risk, high risk, or moderate risk. Disagreements were discussed and consensus obtained. Two cluster RCTs were included in this review and were also quality assessed by the same standards. Results of the quality assessment can be found in S2 Table. The quality of the body of evidence was furthermore evaluated using the Grading of Recommendations, Assessment, Development and Evaluation (GRADE) system[3].

The data extraction was performed using ©Microsoft Excel, version 16.60. Information on study year, author, design, originality, sample size, population age, follow-up period, vaccine types, main findings, secondary outcomes, blinding, and conflicts of interest were extracted. Crude and adjusted estimates were also extracted. All study characteristics are presented in S3 Table, S1 Fig and S4 Table. All studies excluded after full text screening and reason for exclusion are listed in S5 Table.

## **Interventions and outcomes**

To enhance the understanding of the practical implications of currently utilized vaccines we investigated the non-specific effects of standard-titre MCVs on both mortality and morbidity outcomes.

Given the well-established benefits of measles vaccination against the targeted infection[4], conducting RCTs that solely compare zero versus one measles vaccine would be deemed unethical. Consequently, to examine potential non-specific effects of vaccines, trials commonly incorporate an additional early or late vaccine besides the vaccines administered according to the routine child vaccination programme. Several trials with the intervention of an additional early MVC, present the results within the follow-up period until the second vaccine along with results of various further follow-up time. To provide an overview of the non-specific effects of MCVs across these follow-up periods, we performed meta-analyses for both one versus zero, and two versus one MCV on the outcomes of mortality and morbidity. Only estimates from intention-to-treat analyses were included.

To offer a historical and contextual perspective on the research surrounding the non-specific effects of MCVs, we examined non-specific-effects of high-titre MCVs on the outcome of mortality.

## **Statistical methods**

TSA analysis

In the TSA software, the intervention was specified as ’vaccines containing live attenuated measles‘ and the comparator as ‘other vaccine’, ‘other vaccine sequence’, or ‘placebo’. TSA was applied to the dichotomous outcomes of mortality and admissions/consultations, using relative risk (RR) as the effect estimate. Heterogeneity was calculated as I^2^ and the DerSimonian-Laird random effects model [5, 6] was chosen to estimate the between-trial variance. All zero event trials were included using a constant continuity correction of 0.5. All RCTs included in the meta-analysis presented a 95% confidence interval. The alpha spending function was calculated based on alpha = 0.05 and beta = 0.20. The control event proportion (Pc) was based on the observed values of the included RCTs. Heterogeneity correction was implemented using the diversity estimate, D^2^, providing a diversity-adjusted required information size (DARIS). This was incorporated via the ‘model variance option‘ in the TSA software[7, 8]. A relative risk reduction (RRR) of 25% was chosen to match the 25% risk reduction investigated in the included RCTs[9]. For some populations a 33% RRR was also investigated. Trials were included in the analysis independently of risk of bias but only trials with compatible intervention strategies and outcomes were included. TSAs yield either a conclusive or inconclusive result. If a TSA z-curve (green) crosses the boundary of harm or benefit or enters the area of futility (red)[10], the analysis is deemed conclusive meaning that enough evidence has been gathered to suggest that even if more data were included in the analysis, the conclusion would not change. For clarity, S6 Table provides an exact overview of every choice made in the TSA program.

TSA limitations

TSA of the outcome morbidity requires crude data preferably from comparable original trials. Eight trials investigated morbidity. Five came from different populations. Two trials were chosen with matching intervention strategies, but they were re-analyses of other trials. It was ensured that no populations were overlapping to mitigate risk of bias. In sub-group analyses, trials with different intervention strategies were included to gain enough data for conclusive analysis. Different combinations of studies were attempted for the TSA, but the overall quality of sub-group analyses was lower than of the primary analyses.

Forest plots

R was used to perform two forest plots; one based on crude data and another on data adjusted for measles cases, accidental deaths, season, age, and sex. Using the inverse variance method both fixed effect and random effects models were fitted. All trials were included in the forest plots regardless of risk of bias and compatibility.
