# Supplementary material for: Measles vaccines and non-specific effects on mortality or morbidity: A systematic review and meta-analysis
Source: PLoS One. 2025 Jul 2;20(7):e0321982. doi: 10.1371/journal.pone.0321982 (PMC12221017; doi:10.1371/journal.pone.0321982)
Supplement: S1 Fig — Shows how the included trials were categorised by intervention, outcome and study populations. (DOCX) [file pone.0321982.s002.docx]

## **S1 Figure. Overview of trial populations.**


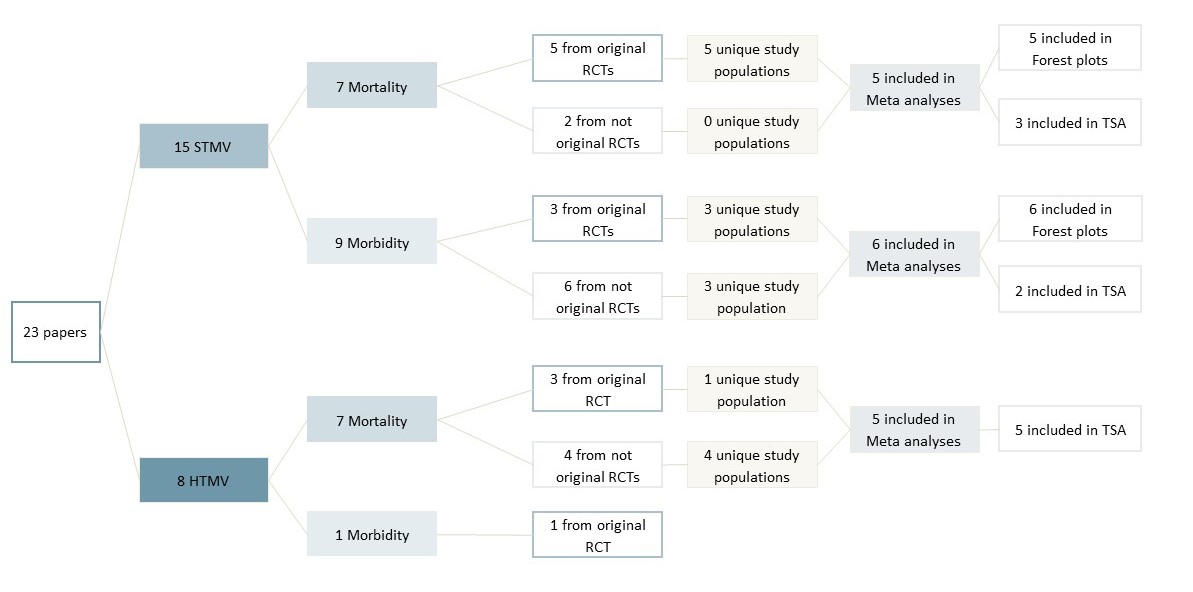


S1 Figure. Shows how the included trials were categorised by intervention, outcome and study populations.
