# Supplementary material for: Measles vaccines and non-specific effects on mortality or morbidity: A systematic review and meta-analysis
Source: PLoS One. 2025 Jul 2;20(7):e0321982. doi: 10.1371/journal.pone.0321982 (PMC12221017; doi:10.1371/journal.pone.0321982)
Supplement: S1 File — (PDF) [file pone.0321982.s003.pdf]

S1 File. Citations excluded with animal and language filters.

Citations excluded as animal studies

1. Symposium on Immunity to Selected Canine Infectious Diseases. Report of the panel. J Am Vet Med Assoc 1970;156(12):1661-8.
2. [Reported cases of communicable reportable diseases in Costa Rica, week no. 52, ending December 30, 1978]. Sem Epidemiol 1978;6(52):1-7.
3. EPI in Cuba: immunization gets top priority. EPI Newsl 1982;4(3):1-3.
4. EPI in the Americas: a regional overview. EPI Newsl 1983;5(5):1-4.
5. Diarrhoea and immunization: a valuable ally? Diarrhoea Dialogue 1984(16):1.
6. How to give an immunization. Salubritas 1986;9(2):3.
7. Global Advisory Group: conclusions and recommendations. EPI Newsl 1986;13(1):4-6.
8. [Immunization: an important aspect often neglected]. Rumah Tangga Kesehatan 1987(8):10-1.
9. [Public health programs have greatly reduced infant mortality in Costa Rica]. Perspect Int Planif Fam 1987(Spec No):33-4.
10. Expanded Programme on Immunization -- Philippines. Virus Inf Exch Newsl South East Asia West Pac 1988;5(2):50.
11. Immunization. Health Technol Dir 1989;9(2):1-15.
12. [Network of researchers on the health aspects of reproduction]. Vie Sante 1990(4):18-21.
13. China achieves great success in planned immunization for children. China Popul Today 1991;8(3):17-8.
14. Diarrhoeal and acute respiratory disease: the current situation. In Point Fact 1991(76):1-3.
15. The State of the World's Children 1993. The age of neglect may be giving way to an age of concern. People Count 1993;3(7):1-4.
16. Improved child survival in Cameroon. Newsl Macro Syst Inst Resour Dev Demogr Health Surv 1993;6(1):3-4.
17. Nigeria high in child deaths -- UNICEF. Niger Pop 1993:39.
18. Senegal links up with the past to protect the future. CVI Forum 1993(5):9-10.
19. Vaccination efforts lauded in Brazil. Vaccine Wkly 1994:12-3.
20. Neonatal tetanus: the final countdown. CVI Forum 1994(8):6-9.
21. Measles elimination by the year 2000. EPI Newsl 1994;16(5):1-2.
22. Health ministry guidelines on measles immunisation. Indian Med Trib 1994;2(4):4.
23. Sustainable development and quality health care. Sante Salud 1994:6.
24. The SWACH Foundation of India. Safe Mother 1994(14):9.
25. Doubt over measles targets prompts new vaccination strategy. CVI Forum 1994(7):2-6.
26. Coping with size: China's efforts pay off. CVI Forum 1994(8):10-1.
27. A Moroccan priority -- making success sustainable. CVI Forum 1994(7):11-2.
28. The progress of human rights in China. Protecting the legitimate rights and interests of women and children. China Popul Today 1996;13(1):18-9.
29. A pneumococcal vaccine to save children of all ages nears final testing. CVI Forum 1996(13):3-11.
30. Twenty years of EPI. EPI Newsl 1997;19(4):1-3.
31. A plan for immediate action, a vision for the future. Interview [with John La Montagne]. CVI Forum 1997(14):7-8.
32. Scientific achievements of the Centre, 1991-1995, in research on child survival. Glimpse 1997;19(2):2-5.
33. Evaluation of Mexico's Universal Vaccination Program. EPI Newsl 1998;20(2):4-5.
34. Spotlight: Azerbaijan. Popul Today 1999;27(2):7.
35. A glimpse at program for children's development in China. China Popul Today 1999;16(3):19-20.
36. Progress towards reducing measles mortality and eliminating measles, WHO Eastern Mediterranean Region, 1997-2007. Wkly Epidemiol Rec 2008;83(11):97-104.
37. WHO Guidelines Approved by the Guidelines Review Committee. Response to Measles Outbreaks in Measles Mortality Reduction Settings: Immunization, Vaccines and Biologicals. Geneva: World Health Organization
Copyright © 2009, World Health Organization. 2009.
38. WHO Guidelines Approved by the Guidelines Review Committee. Pocket Book of Hospital Care for Children: Guidelines for the Management of Common Childhood Illnesses. Geneva: World Health Organization
Copyright © World Health Organization 2013. 2013.
39. Association of BCG, DTP, and measles containing vaccines with childhood mortality: systematic review. Bmj 2017;356:j1241. doi: 10.1136/bmj.j1241 [published Online First: 20170308]
40. Abdulah DM, Hassan AB. Exploration of Association Between Respiratory Vaccinations With Infection and Mortality Rates of COVID-19. Disaster Med Public Health Prep 2021;17:e14. doi: 10.1017/dmp.2021.47 [published Online First: 20210216]
41. Abdullahi AM, Sarmast ST, Singh R. Molecular Biology and Epidemiology of Neurotropic Viruses. Cureus 2020;12(8):e9674. doi: 10.7759/cureus.9674 [published Online First: 20200811]

42. Abe Y, Hashimoto K, Watanabe M, et al. Characteristics of viruses derived from nude mice with persistent measles virus infection. J Virol 2013;87(8):4170-5. doi: 10.1128/jvi.03117-12 [published Online First: 20130123]
43. Ablett RE. Prophylaxis and clinical evaluation concerning measles virus for distemper immunization. J Am Vet Med Assoc 1970;156(12):1766-8.
44. Aborode AT, Babatunde AO, Osayomwanbor BS, et al. Measles outbreak amidst COVID-19 pandemic in Africa: grappling with looming crises. Trop Med Health 2021;49(1):89. doi: 10.1186/s41182-021-00375-3 [published Online First: 20211102]
45. Abu-Raya B, Kollmann TR, Marchant A, et al. The Immune System of HIV-Exposed Uninfected Infants. Front Immunol 2016;7:383. doi: 10.3389/fimmu.2016.00383 [published Online First: 20160928]
46. Ackermann O. Early immunization against canine distemper and hepatitis, using combined vaccines. J Am Vet Med Assoc 1970;156(12):1755-8.
47. Adeboye M, Adesiyun O, Adegboye A, et al. Measles in a tertiary institution in bida, niger state, Nigeria: prevalence, immunization status and mortality pattern. Oman Med J 2011;26(2):114-7. doi: 10.5001/omj.2011.28
48. Afzal A, Saleel CA, Bhattacharyya S, et al. Merits and Limitations of Mathematical Modeling and Computational Simulations in Mitigation of COVID-19 Pandemic: A Comprehensive Review. Arch Comput Methods Eng 2022;29(2):1311-37. doi: 10.1007/s11831-021-09634-2 [published Online First: 20210811]
49. Agranovski IE, Safatov AS, Borodulin AI, et al. Inactivation of viruses in bubbling processes utilized for personal bioaerosol monitoring. Appl Environ Microbiol 2004;70(12):6963-7. doi: 10.1128/aem.70.12.6963-6967.2004
50. Agrawal A, Kolhapure S, Di Pasquale A, et al. Vaccine Hesitancy as a Challenge or Vaccine Confidence as an Opportunity for Childhood Immunisation in India. Infect Dis Ther 2020;9(3):421-32. doi: 10.1007/s40121-020-00302-9 [published Online First: 20200523]
51. Ahmadu BU, Yakubu M, Abdullahi IB, et al. Newborn measles antibody profile in a teaching hospital: Can sex of babies determine measles IgG acquisition from their respective mothers? J Pediatr Genet 2013;2(1):21-4. doi: 10.3233/pge-13043
52. Ahmed EM, Solyman SM, Mohamed N, et al. Antiviral activity of Ribavirin nano-particles against measles virus. Cell Mol Biol (Noisy-le-grand) 2018;64(9):24-32. [published Online First: 20180630]
53. Alberti KP, King LA, Burny ME, et al. Reactive vaccination as an effective tool for measles outbreak control in measles mortality reduction settings, Democratic Republic of Congo, 2005-2006. Int Health 2010;2(1):65-8. doi: 10.1016/j.inhe.2009.12.009
54. Albrecht P, Lorenz D, Klutch MJ, et al. Fatal measles infection in marmosets pathogenesis and prophylaxis. Infect Immun 1980;27(3):969-78. doi: 10.1128/iai.27.3.969-978.1980
55. Albrecht P, Shabo AL, Burns GR, et al. Experimental measles encephalitis in normal and cyclophosphamide-treated rhesus monkeys. J Infect Dis 1972;126(2):154-61. doi: 10.1093/infdis/126.2.154
56. Alemayehu K, Oljira L, Demena M, et al. Prevalence and Determinants of Diarrheal Diseases among Under-Five Children in Horo Guduru Wollega Zone, Oromia Region, Western Ethiopia: A Community-Based Cross-Sectional Study. Can J Infect Dis Med Microbiol 2021;2021:5547742. doi: 10.1155/2021/5547742 [published Online First: 20210623]
57. Allen C, Paraskevakou G, Iankov I, et al. Interleukin-13 Displaying Retargeted Oncolytic Measles Virus Strains Have Significant Activity Against Gliomas With Improved Specificity. Mol Ther 2008;16(9):1556-64. doi: 10.1038/mt.2008.152 [published Online First: 20161208]
58. Ameh CA, Sufiyan MB, Jacob M, et al. Evaluation of the Measles Surveillance System in Kaduna State, Nigeria (2010-2012). Online J Public Health Inform 2016;8(3):e206. doi: 10.5210/ojphi.v8i3.7089 [published Online First: 20161128]
59. Andersen A, Fisker AB, Rodrigues A, et al. National Immunization Campaigns with Oral Polio Vaccine Reduce All-Cause Mortality: A Natural Experiment within Seven Randomized Trials. Front Public Health 2018;6:13. doi: 10.3389/fpubh.2018.00013 [published Online First: 20180202]
60. Andrie EK, Sakellari E, Barbouni A, et al. Vaccination Coverage during Childhood and Adolescence among Undergraduate Health Science Students in Greece. Children (Basel) 2022;9(10) doi: 10.3390/children9101553 [published Online First: 20221013]
61. Auwaerter PG, Rota PA, Elkins WR, et al. Measles virus infection in rhesus macaques: altered immune responses and comparison of the virulence of six different virus strains. J Infect Dis 1999;180(4):950-8. doi: 10.1086/314993
62. Azad MB, Wade KH, Timpson NJ. FUT2 secretor genotype and susceptibility to infections and chronic conditions in the ALSPAC cohort. Wellcome Open Res 2018;3:65. doi: 10.12688/wellcomeopenres.14636.2 [published Online First: 20180925]
63. Backhaus PS, Veinalde R, Hartmann L, et al. Immunological Effects and Viral Gene Expression Determine the Efficacy of Oncolytic Measles Vaccines Encoding IL-12 or IL-15 Agonists. Viruses 2019;11(10) doi: 10.3390/v11100914 [published Online First: 20191003]
64. Baguune B, Ndago JA, Adokiya MN. Immunization dropout rate and data quality among children 12-23 months of age in Ghana. Arch Public Health 2017;75:18. doi: 10.1186/s13690-017-0186-8 [published Online First: 20170417]
65. Bahreynian M, Qorbani M, Naderimagham S, et al. Burden of disease attributable to vitamin A deficiency in Iranian population aged less than five years: findings from the global burden of disease study 2010. J Diabetes Metab Disord 2017;16:32. doi: 10.1186/s40200-017-0313-7 [published Online First: 20170809]
66. Bain S. Indonesia lowers infant mortality. Front Lines 1991:16.
67. Bangham CR. Passively acquired antibodies to respiratory syncytial virus impair the secondary cytotoxic T-cell response in the neonatal mouse. Immunology 1986;59(1):37-41.
68. Bansal P, Srivastava A, Aggarwal R, et al. Measles in immunized medical residents: Case series with brief review. J Family Med Prim Care 2021;10(11):4311-13. doi: 10.4103/jfmpc.jfmpc_714_21 [published Online First: 20211129]
69. Barnard DL, Stowell VD, Seley KL, et al. Inhibition of measles virus replication by 5'-nor carbocyclic adenosine analogues. Antivir Chem Chemother 2001;12(4):241-50. doi: 10.1177/095632020101200405

70. Barrett T, Blixenkrone-Møller M, Domingo M, et al. Round table on morbilliviruses in marine mammals. Vet Microbiol 1992;33(1-4):287-95. doi: 10.1016/0378-1135(92)90056-y
71. Barsky D, Palmer AE, London WT, et al. Use of immune serum globulin (human) to reduce mortality in newly imported rhesus monkeys (Macaca mulatta). J Med Primatol 1976;5(3):150-9. doi: 10.1159/000459942
72. Basak M, Chaudhuri SB, Ishore K, et al. Pattern and Trend of Morbidity in the Infectious Disease Ward of North Bengal Medical College and Hospital. J Clin Diagn Res 2015;9(11):Lc01-4. doi: 10.7860/jcdr/2015/15202.6741 [published Online First: 20151101]
73. Baya B. [High infant mortality rates that can be substantially lowered]. Pop Sahel 1989(10):6-9.
74. Bbaale E. Determinants of diarrhoea and acute respiratory infection among under-fives in Uganda. Australas Med J 2011;4(7):400-9. doi: 10.4066/amj.2011.723 [published Online First: 20110731]
75. Bégué P. [Varicella and zoster vaccines]. Virologie (Montrouge) 2006;10(6):407-14. doi: 10.1684/vir.2011.8640
76. Benchimol EI, Tse F, Carroll MW, et al. Canadian Association of Gastroenterology Clinical Practice Guideline for Immunizations in Patients With Inflammatory Bowel Disease (IBD)-Part 1: Live Vaccines. J Can Assoc Gastroenterol 2021;4(4):e59-e71. doi: 10.1093/jcag/gwab015 [published Online First: 20210729]
77. Benedicto-Matambo P, Bines JE, Malamba-Banda C, et al. Leveraging Beneficial Off-Target Effects of Live-Attenuated Rotavirus Vaccines. Vaccines (Basel) 2022;10(3) doi: 10.3390/vaccines10030418 [published Online First: 20220310]
78. Benn CS, Martins CL, Andersen A, et al. Measles Vaccination in Presence of Measles Antibody May Enhance Child Survival. Front Pediatr 2020;8:20. doi: 10.3389/fped.2020.00020 [published Online First: 20200207]
79. Bhattacharjee S, Yadava PK. Measles virus: Background and oncolytic virotherapy. Biochem Biophys Rep 2018;13:58-62. doi: 10.1016/j.bbrep.2017.12.004 [published Online First: 20180102]
80. Bhattacharya S, Singh A. Phasing out of the Universal Mega Dose of Vitamin-A Prophylaxis to Avoid Toxicity. AIMS Public Health 2017;4(1):38-46. doi: 10.3934/publichealth.2017.1.38 [published Online First: 20170120]
81. Bhella D, Ralph A, Yeo RP. Conformational flexibility in recombinant measles virus nucleocapsids visualised by cryo-negative stain electron microscopy and real-space helical reconstruction. J Mol Biol 2004;340(2):319-31. doi: 10.1016/j.jmb.2004.05.015
82. Bittle JL. Newer knowledge on the use of measles vaccine for canine distemper prophylaxis. J Am Vet Med Assoc 1970;156(12):1752-5.
83. Blixenkrone-Møller M. Biological properties of phocine distemper virus and canine distemper virus. APMIS Suppl 1993;36:1-51.
84. Bogomolova NN, Burgasova MP, Chaplygina NM, et al. [Experimental measles infection in rodents]. Vopr Virusol 1989;34(5):590-5.
85. Bolongaita S, Villano D, Memirie ST, et al. Modeling the relative risk of incidence and mortality of select vaccine-preventable diseases by wealth group and geographic region in Ethiopia. PLOS Glob Public Health 2022;2(8) doi: 10.1371/journal.pgph.0000819 [published Online First: 20220831]
86. Bonnan M. Does disease-irrelevant intrathecal synthesis in multiple sclerosis make sense in the light of tertiary lymphoid organs? Front Neurol 2014;5:27. doi: 10.3389/fneur.2014.00027 [published Online First: 20140311]
87. Bozzola E, Bozzola M, Calcaterra V, et al. Infectious diseases and vaccination strategies: how to protect the "unprotectable"? ISRN Prev Med 2013;2013:765354. doi: 10.5402/2013/765354 [published Online First: 20130403]
88. Brachman PS. Epidemiology. In: Baron S, ed. Medical Microbiology. Galveston (TX): University of Texas Medical Branch at Galveston
Copyright © 1996, The University of Texas Medical Branch at Galveston. 1996.
89. Brannen DE, Alhammad A, Branum M, et al. International Air Travel to Ohio, USA, and the Impact on Malaria, Influenza, and Hepatitis A. Scientifica (Cairo) 2016;2016:8258946. doi: 10.1155/2016/8258946 [published Online First: 20160331]
90. Bringolf F, Herren M, Wyss M, et al. Dimerization Efficiency of Canine Distemper Virus Matrix Protein Regulates Membrane-Budding Activity. J Virol 2017;91(16) doi: 10.1128/jvi.00521-17 [published Online First: 20170727]
91. Brown AF. Cuban health system -- call it superior? Links 1992;9(2):5-6.
92. Bukreyev A, Skiadopoulos MH, McAuliffe J, et al. More antibody with less antigen: can immunogenicity of attenuated live virus vaccines be improved? Proc Natl Acad Sci U S A 2002;99(26):16987-91. doi: 10.1073/pnas.252649299 [published Online First: 20021213]
93. Burnett M. Measles (rubeola): an ongoing series on infectious diseases of importance to the deployed special forces. J Spec Oper Med 2013;13(1):66-7.
94. Busse E, Helmholz M. [The influence of L-dopa and of thymus fraction on the survival rate of whole-body-irradiated mice]. Arch Geschwulstforsch 1982;52(4):253-7.
95. Byington DP, Johnson KP. Subacute sclerosing panencephalitis virus in immunosuppressed adult hamster. Lab Invest 1975;32(1):91-7.
96. Cabezas C, Ramos F, Vega M, et al. [IMPACT OF THE IMMUNIZATION PROGRAM INTEGRATED TO THE EXPANDED IMMUNIZATION PROGRAM(EPI) IN HUANTA,1994-1997]. Rev Gastroenterol Peru 2000;20(3):201-12.
97. Cairns KL, Nandy R, Grais RF. Challenges in measuring measles case fatality ratios in settings without vital registration. Emerg Themes Epidemiol 2010;7(1):4. doi: 10.1186/1742-7622-7-4 [published Online First: 20100719]
98. Calder PC, Jackson AA. Undernutrition, infection and immune function. Nutr Res Rev 2000;13(1):3-29. doi: 10.1079/095442200108728981
99. Campbell H, Cutting W. Integrating treatment and prevention: the small hospital. Child Health Dialogue 1996(2):1.
100. Campos LF, Şentürk D, Chen Y, et al. Bias and estimation under misspecification of the risk period in self-controlled case series studies. Stat (Int Stat Inst) 2017;6(1):373-89. doi: 10.1002/sta4.166 [published Online First: 20171020]

101. Capozzo AV, Ramírez K, Polo JM, et al. Neonatal immunization with a Sindbis virus-DNA measles vaccine induces adult-like neutralizing antibodies and cell-mediated immunity in the presence of maternal antibodies. J Immunol 2006;176(9):5671-81. doi: 10.4049/jimmunol.176.9.5671
102. Caputo M, Raupach-Rosin H, Karch A, et al. Vaccinations and Infections Are Associated With Unrelated Antibody Titers: An Analysis From the German Birth Cohort Study LISA. Front Pediatr 2019;7:254. doi: 10.3389/fped.2019.00254 [published Online First: 20190625]
103. Carrasco-Garrido P, Gallardo-Pino C, Jiménez-García R, et al. Incidence of adverse reactions to vaccines in a paediatric population. Clin Drug Investig 2004;24(8):457-63. doi: 10.2165/00044011-200424080-00004
104. Carsillo T, Traylor Z, Choi C, et al. hsp72, a host determinant of measles virus neurovirulence. J Virol 2006;80(22):11031-9. doi: 10.1128/jvi.01438-06 [published Online First: 20060913]
105. Casale M, Di Maio N, Verde V, et al. Response to Measles, Mumps and Rubella (MMR) Vaccine in Transfusion-Dependent Patients. Vaccines (Basel) 2021;9(6) doi: 10.3390/vaccines9060561 [published Online First: 20210527]
106. Cazes MH. [Demographic and evolutionary dynamics of an isolate: the Dogon of Boni]. Etudes Mali 1991(44):57-64.
107. Celiloğlu C, Tolunay O, Çelik Ü. Evaluation of Pediatric Measles Cases Hospitalized in 2019. Turk Arch Pediatr 2021;56(4):328-31. doi: 10.5152/TurkArchPediatr.2021.20216 [published Online First: 20210701]
108. Chalmers WS, Baxendale W. A comparison of canine distemper vaccine and measles vaccine for the prevention of canine distemper in young puppies. Vet Rec 1994;135(15):349-53. doi: 10.1136/vr.135.15.349
109. Chambouleyron R, Barbosa BC, Bombardi FA, et al. 'Formidable contagion': epidemics, work and recruitment in Colonial Amazonia (1660-1750). Hist Cienc Saude Manguinhos 2011;18(4):987-1004. doi: 10.1590/s0104-59702011000400002
110. Chan SP. Induction of chronic measles encephalitis in C57BL/6 mice. J Gen Virol 1985;66 (Pt 9):2071-6. doi: 10.1099/0022-1317-66-9-2071
111. Chard AN, Gacic-Dobo M, Diallo MS, et al. Routine Vaccination Coverage - Worldwide, 2019. MMWR Morb Mortal Wkly Rep 2020;69(45):1706-10. doi: 10.15585/mmwr.mm6945a7 [published Online First: 20201113]
112. Chen PH, Miller GF, Powell DA. Colitis in a female tamarin (Saguinus mystax). Contemp Top Lab Anim Sci 2000;39(2):47-9.
113. Cheng X, Shi R. Social changes and the evolution of reproduction patterns in Xishuangbanna. Chin J Popul Sci 1994;6(1):25-36.
114. Choi YK, Simon MA, Kim DY, et al. Fatal measles virus infection in Japanese macaques (Macaca fuscata). Vet Pathol 1999;36(6):594-600. doi: 10.1354/vp.36-6-594
115. Chumakov K, Avidan MS, Benn CS, et al. Old vaccines for new infections: Exploiting innate immunity to control COVID-19 and prevent future pandemics. Proc Natl Acad Sci U S A 2021;118(21) doi: 10.1073/pnas.2101718118
116. Clements CJ, Kane M, Hu DJ, et al. [Hepatitis B vaccine: a new force against pandemic diseases]. Foro Mund Salud 1990;11(2):165-8.
117. Clements J. Opportunities to immunise. Child Health Dialogue 1996(2):3-4.
118. Cockcroft A, Usman MU, Nyamucherera OF, et al. Why children are not vaccinated against measles: a cross-sectional study in two Nigerian States. Arch Public Health 2014;72(1):48. doi: 10.1186/2049-3258-72-48 [published Online First: 20141229]
119. Cody JJ, Hurst DR. Promising oncolytic agents for metastatic breast cancer treatment. Oncolytic Virother 2015;4:63-73. doi: 10.2147/ov.S63045 [published Online First: 20150603]
120. Collier MG, Cierzniewski A, Duszynski T, et al. Measles Outbreak Associated With International Travel, Indiana, 2011. J Pediatric Infect Dis Soc 2013;2(2):110-8. doi: 10.1093/jpids/pis132 [published Online First: 20130130]
121. Contreras EM, Johnston GP, Buchholz DW, et al. Roles of Cholesterol in Early and Late Steps of the Nipah Virus Membrane Fusion Cascade. J Virol 2021;95(6) doi: 10.1128/jvi.02323-20 [published Online First: 20210224]
122. Corsi DJ, Bassani DG, Kumar R, et al. Gender inequity and age-appropriate immunization coverage in India from 1992 to 2006. BMC Int Health Hum Rights 2009;9 Suppl 1(Suppl 1):S3. doi: 10.1186/1472-698x-9-s1-s3 [published Online First: 20091014]
123. Cox R, Plemper RK. The paramyxovirus polymerase complex as a target for next-generation anti-paramyxovirus therapeutics. Front Microbiol 2015;6:459. doi: 10.3389/fmicb.2015.00459 [published Online First: 20150512]
124. Cox RM, Sourimant J, Govindarajan M, et al. Therapeutic targeting of measles virus polymerase with ERDRP-0519 suppresses all RNA synthesis activity. PLoS Pathog 2021;17(2):e1009371. doi: 10.1371/journal.ppat.1009371 [published Online First: 20210223]
125. Curtis B, Liberato N, Rulien M, et al. Examination of the safety of pediatric vaccine schedules in a non-human primate model: assessments of neurodevelopment, learning, and social behavior. Environ Health Perspect 2015;123(6):579-89. doi: 10.1289/ehp.1408257 [published Online First: 20150218]
126. Das JK, Salam RA, Arshad A, et al. Systematic Review and Meta-Analysis of Interventions to Improve Access and Coverage of Adolescent Immunizations. J Adolesc Health 2016;59(4s):S40-s48. doi: 10.1016/j.jadohealth.2016.07.005
127. Davitoiu AM, Spatariu L, Plesca DA, et al. Review of the measles epidemic in children from Central Eastern Europe in the third millennium. Exp Ther Med 2021;22(2):816. doi: 10.3892/etm.2021.10248 [published Online First: 20210602]
128. De Clercq E, Cools M, Balzarini J, et al. Antiviral activities of 5-ethynyl-1-beta-D-ribofuranosylimidazole-4- carboxamide and related compounds. Antimicrob Agents Chemother 1991;35(4):679-84. doi: 10.1128/aac.35.4.679
129. De Clercq E, Descamps J, De Somer P, et al. (S)-9-(2,3-Dihydroxypropyl)adenine: an aliphatic nucleoside analog with broad-spectrum antiviral activity. Science 1978;200(4341)
130. De Jong JG, Winkler KC. SURVIVAL OF MEASLES VIRUS IN AIR. Nature 1964;201:1054-5. doi: 10.1038/2011054a0
131. de St Maurice A, Edwards KM. Vaccine Hesitancy in Children-A Call for Action. Children (Basel) 2016;3(2) doi: 10.3390/children3020007 [published Online First: 20160429]

132. de Vries RD, de Swart RL. Evaluating measles vaccines: can we assess cellular immunity? Expert Rev Vaccines 2012;11(7):779-82. doi: 10.1586/erv.12.45
133. de Vries RD, Lemon K, Ludlow M, et al. In vivo tropism of attenuated and pathogenic measles virus expressing green fluorescent protein in macaques. J Virol 2010;84(9):4714-24. doi: 10.1128/jvi.02633-09 [published Online First: 20100224]
134. Debnath SC, Haque ME, Hasan DMM, et al. Undernutrition and Morbidity Profile of Exclusively Breastfeeding Children: A Cross-sectional Study. Int J Prev Med 2018;9:55. doi: 10.4103/ijpvm.IJPVM_201_17 [published Online First: 20180626]
135. Dejene H, Girma D, Geleta LA, et al. Vaccination timeliness and associated factors among children aged 12-23 months in Debre Libanos district of North Shewa Zone, Oromia Regional State, Ethiopia. Front Pediatr 2022;10:867846. doi: 10.3389/fped.2022.867846 [published Online First: 20220727]
136. Desjardins M, Mitre X, Sherman AC, et al. Safety of Live-Attenuated Measles, Mumps, and Rubella Vaccine Administered Within 2 Years of Hematopoietic Cell Transplant. Open Forum Infect Dis 2021;8(12):ofab504. doi: 10.1093/ofid/ofab504 [published Online First: 20211123]
137. Dhar GM. An epidemiological approach to the understanding and control of acute respiratory infections in Indian children. Indian J Matern Child Health 1992;3(1):4-7.
138. Diosi P. LONG TERM CHANGES IN THE NATURAL HISTORY OF INFECTIOUS DISEASES. Centaurus 1964;9:287-91.
139. Domai FM, Agrupis KA, Han SM, et al. Measles outbreak in the Philippines: epidemiological and clinical characteristics of hospitalized children, 2016-2019. Lancet Reg Health West Pac 2022;19:100334. doi: 10.1016/j.lanwpc.2021.100334 [published Online First: 20211215]
140. Douglas JW. The extent of breast feeding in Great Britain in 1946, with special reference to the health and survival of children. J Obstet Gynaecol Br Emp 1950;57:335-61.
141. Duclos P, Okwo-Bele JM, Gacic-Dobo M, et al. Global immunization: status, progress, challenges and future. BMC Int Health Hum Rights 2009;9 Suppl 1(Suppl 1):S2. doi: 10.1186/1472-698x-9-s1-s2 [published Online First: 20091014]
142. Dutta PK. Scope of health systems research in Child Survival and Safe Motherhood programme. Indian J Matern Child Health 1993;4(2):38-41.
143. E DEC, Descamps J, P DES, et al. (S)-9-(2,3-Dihydroxypropyl)adenine: An Aliphatic Nucleoside Analog with Broad-Spectrum Antiviral Activity. Science 1978;200(4341):563-5. doi: 10.1126/science.200.4341.563
144. Ehrengruber MU, Hennou S, Büeler H, et al. Gene transfer into neurons from hippocampal slices: comparison of recombinant Semliki Forest Virus, adenovirus, adeno-associated virus, lentivirus, and measles virus. Mol Cell Neurosci 2001;17(5):855-71. doi: 10.1006/mcne.2001.0982
145. Ekhaguere OA, Oluwafemi RO, Badejoko B, et al. Automated phone call and text reminders for childhood immunisations (PRIMM): a randomised controlled trial in Nigeria. BMJ Glob Health 2019;4(2):e001232. doi: 10.1136/bmjgh-2018-001232 [published Online First: 20190403]
146. El Arifeen S. Centre for IMCI Research and Training: a centre of excellence. Glimpse 1998;20(1):5-6.
147. El Kasmi KC, Theisen D, Brons NH, et al. A hemagglutinin-derived peptide-vaccine ignored by virus-neutralizing passive antibodies, protects against murine measles encephalitis. Vaccine 1999;17(19):2436-45. doi: 10.1016/s0264-410x(99)00008-0
148. Ellis H. 'The Sister' in the early days of the NHS. J Perioper Pract 2016;26(4):90-2. doi: 10.1177/175045891602600406
149. Ene L, Duiculescu D, Radoi R, et al. Subacute myoclonic measles encephalitis - An opportunistic HIV-associated infection. Front Cell Neurosci 2023;17:1113935. doi: 10.3389/fncel.2023.1113935 [published Online First: 20230404]
150. Engeland CE, Grossardt C, Veinalde R, et al. CTLA-4 and PD-L1 checkpoint blockade enhances oncolytic measles virus therapy. Mol Ther 2014;22(11):1949-59. doi: 10.1038/mt.2014.160 [published Online First: 20140826]
151. Estofolete CF, de Andrade Gandolfi F, de Aguiar Milhim BH, et al. Reduced Prevalence of Measles Antibodies in a Cohort of Brazilian Children under 15 Years of Age. Vaccines (Basel) 2022;10(10) doi: 10.3390/vaccines10101570 [published Online First: 20220920]
152. Etchart N, Desmoulins PO, Chemin K, et al. Dendritic cells recruitment and in vivo priming of CD8+ CTL induced by a single topical or transepithelial immunization via the buccal mucosa with measles virus nucleoprotein. J Immunol 2001;167(1):384-91. doi: 10.4049/jimmunol.167.1.384
153. Ezeonu C, Uneke C, Ojukwu J, et al. The pattern of pediatric respiratory illnesses admitted in ebonyi state university teaching hospital South-East Nigeria. Ann Med Health Sci Res 2015;5(1):65-70. doi: 10.4103/2141-9248.149792
154. Fantetti KN, Gray EL, Ganesan P, et al. Interferon gamma protects neonatal neural stem/progenitor cells during measles virus infection of the brain. J Neuroinflammation 2016;13(1):107. doi: 10.1186/s12974-016-0571-1 [published Online First: 20160513]
155. Fargues P. [The month of birth: a factor of inequality before death]. Pop Sahel 1989(10):20-4.
156. Farra A, Pagonendji M, Manikariza A, et al. Epidemiology of primary rubella infection in the Central African Republic: data from measles surveillance, 2007-2014. BMC Infect Dis 2016;16(1):505. doi: 10.1186/s12879-016-1842-2 [published Online First: 20160923]
157. Fayaz A, Rajak KK, Kumar A, et al. Development and characterization of mouse monoclonal antibodies to canine morbillivirus. Biologicals 2022;79:19-26. doi: 10.1016/j.biologicals.2022.08.005 [published Online First: 20220909]
158. Feikin DR, Flannery B, Hamel MJ, et al. Vaccines for Children in Low- and Middle-Income Countries. In: Black RE, Laxminarayan R, Temmerman M, et al., eds. Reproductive, Maternal, Newborn, and Child Health: Disease Control Priorities, Third Edition (Volume 2). Washington (DC): The International Bank for Reconstruction and Development / The World Bank © 2016 International Bank for Reconstruction and Development / The World Bank. 2016.

159. Feng C, Bu Y, Cai J, et al. Persistent and Severe Viral Replication in PBMCs with Moderate Immunosuppression Served an Alternative Novel Pathogenic Mechanism for Canine Morbillivirus. Microbiol Spectr 2023;11(1):e0406022. doi: 10.1128/spectrum.04060-22 [published Online First: 20221219]
160. Figueira TN, Mendonça DA, Gaspar D, et al. Structure-Stability-Function Mechanistic Links in the Anti-Measles Virus Action of Tocopherol-Derivatized Peptide Nanoparticles. ACS Nano 2018;12(10):9855-65. doi: 10.1021/acsnano.8b01422 [published Online First: 20180924]
161. Finke D, Brinckmann UG, ter Meulen V, et al. Gamma interferon is a major mediator of antiviral defense in experimental measles virus-induced encephalitis. J Virol 1995;69(9):5469-74. doi: 10.1128/jvi.69.9.5469-5474.1995
162. Finke D, Liebert UG. CD4+ T cells are essential in overcoming experimental murine measles encephalitis. Immunology 1994;83(2):184-9.
163. Fisker AB, Benn CS, Diness BR, et al. The Effect of 50 000 IU Vitamin A with BCG Vaccine at Birth on Growth in the First Year of Life. J Trop Med 2011;2011:570170. doi: 10.1155/2011/570170 [published Online First: 20110908]
164. Fisker AB, Thysen SM. Implementation and assessment of vaccination programmes: the importance of vaccination sequence for overall health outcomes. Hum Vaccin Immunother 2018;14(12):2900-03. doi: 10.1080/21645515.2018.1496771 [published Online First: 20180828]
165. Fisker AB, Aaby P, Bale C, et al. Does the effect of vitamin A supplements depend on vaccination status? An observational study from Guinea-Bissau. BMJ Open 2012;2(1):e000448. doi: 10.1136/bmjopen-2011-000448 [published Online First: 20120112]
166. Foley E, Breit S, Marsh C, et al. Attitudes toward Rubella and Varicella Vaccination during Preconception Care. Kans J Med 2021;14:215-19. doi: 10.17161/kjm.vol1415205 [published Online First: 20210901]
167. Franke F, Coulon L, Renaudat C, et al. Epidemiologic surveillance system implemented in the Hautes-Alpes District, France, during the Winter Olympic Games, Torino 2006. Euro Surveill 2006;11(12):17-18. doi: 10.2807/esm.11.12.00671-en
168. Fusciello M, Ylösmäki E, Feola S, et al. A novel cancer vaccine for melanoma based on an approved vaccine against measles, mumps, and rubella. Mol Ther Oncolytics 2022;25:137-45. doi: 10.1016/j.omto.2022.04.002 [published Online First: 20220419]
169. Gaigbe Togbe V. [Seasonality and cause of infant deaths in Yaounde]. Ann IFORD 1988;12(2):97-126.
170. Galvez Tan JZ. Health and nutrition and the role of the family. Popul Forum 1990(1):12-4.
171. Garg RK, Pandey S, Nigam H, et al. Case Report: An Unusual Case of Subacute Sclerosing Panencephalitis with Distinctive Clinical and Neuroimaging Features. Am J Trop Med Hyg 2023 doi: 10.4269/ajtmh.22-0731 [published Online First: 20230313]
172. Gdalevich M, Ephros M, Mimouni D, et al. Measles epidemic in Israel-successful containment in the military. Prev Med 2000;31(6):649-51. doi: 10.1006/pmed.2000.0757
173. Gerard SP, Kyrousis E, Zachariah R. Measles in the Democratic Republic of Congo: an urgent wake-up call to adapt vaccination implementation strategies. Public Health Action 2014;4(1):6-8. doi: 10.5588/pha.13.0099
174. Getchell M, Mantaring EJ, Yee K, et al. Cost-effectiveness of sub-national geographically targeted vaccination programs: A systematic review. Vaccine 2023;41(14):2320-28. doi: 10.1016/j.vaccine.2023.02.006 [published Online First: 20230211]
175. Gnaneshan S, Brown KE, Green J, et al. On-line global/WHO-European regional measles nucleotide surveillance. Euro Surveill 2008;13(19) [published Online First: 20080508]
176. Goldman GS. Examples of Outcome Reporting Bias in Vaccine Studies: Illustrating How Perpetuating Medical Consensus Can Impede Progress in Public Health. Cureus 2022;14(9):e29399. doi: 10.7759/cureus.29399 [published Online First: 20220921]
177. Goodman RA, Manton KG, Nolan TF, Jr., et al. Mortality data analysis using a multiple-cause approach. Jama 1982;247(6):793-6.
178. Gourlay JA. Comments on the use of measles virus for distemper prophylaxis. J Am Vet Med Assoc 1970;156(12):1769-70.
179. Grais RF, Strebel P, Mala P, et al. Measles vaccination in humanitarian emergencies: a review of recent practice. Confl Health 2011;5(1):21. doi: 10.1186/1752-1505-5-21 [published Online First: 20110926]
180. Gras A, Parada M, Vallès J, et al. The Role of Traditional Plant Knowledge in the Fight Against Infectious Diseases: A Meta-Analytic Study in the Catalan Linguistic Area. Front Pharmacol 2021;12:744616. doi: 10.3389/fphar.2021.744616 [published Online First: 20211011]
181. Graves MC, Silver SM, Choppin PW. Measles virus polypeptides synthesis in infected cells. Virology 1978;86(1):254-63. doi: 10.1016/0042-6822(78)90025-9
182. Green MG, Petroff N, La Perle KMD, et al. Characterization of Cotton Rat (Sigmodon hispidus) Eosinophils, Including Their Response to Respiratory Syncytial Virus Infection. Comp Med 2018;68(1):31-40.
183. Griffin DE, Mullinix J, Narayan O, et al. Age dependence of viral expression: comparative pathogenesis of two rodent-adapted strains of measles virus in mice. Infect Immun 1974;9(4):690-5. doi: 10.1128/iai.9.4.690-695.1974
184. Groaz E, De Clercq E, Herdewijn P. Anno 2021: Which antivirals for the coming decade? Annu Rep Med Chem 2021;57:49-107. doi: 10.1016/bs.armc.2021.09.004 [published Online First: 20211103]
185. Grossardt C, Engeland CE, Bossow S, et al. Granulocyte-macrophage colony-stimulating factor-armed oncolytic measles virus is an effective therapeutic cancer vaccine. Hum Gene Ther 2013;24(7):644-54. doi: 10.1089/hum.2012.205
186. Güven E. Gene Expression Characteristics of Tumor and Adjacent Non-Tumor Tissues of Pancreatic Ductal Adenocarcinoma (PDAC) In-Silico. Iran J Biotechnol 2022;20(1):e3092. doi: 10.30498/ijb.2021.292558.3092 [published Online First: 20220101]
187. Haffejee IE. Child health in South Africa -- past, present and future. Glob Child Health New Rev 1995;3(1):18.
188. Hahm B, Trifilo MJ, Zuniga EI, et al. Viruses evade the immune system through type I interferon-mediated STAT2-dependent, but STAT1-independent, signaling. Immunity 2005;22(2):247-57. doi: 10.1016/j.immuni.2005.01.005

189. Hanauer JDS, Rengstl B, Kleinlützum D, et al. CD30-targeted oncolytic viruses as novel therapeutic approach against classical Hodgkin lymphoma. Oncotarget 2018;9(16):12971-81. doi: 10.18632/oncotarget.24191 [published Online First: 20180112]
190. Hashiguchi T, Fukuda Y, Matsuoka R, et al. Structures of the prefusion form of measles virus fusion protein in complex with inhibitors. Proc Natl Acad Sci U S A 2018;115(10):2496-501. doi: 10.1073/pnas.1718957115 [published Online First: 20180220]
191. Hashiguchi T, Kajikawa M, Maita N, et al. Crystal structure of measles virus hemagglutinin provides insight into effective vaccines. Proc Natl Acad Sci U S A 2007;104(49):19535-40. doi: 10.1073/pnas.0707830104 [published Online First: 20071114]
192. Hashiguchi T, Maenaka K, Yanagi Y. Measles virus hemagglutinin: structural insights into cell entry and measles vaccine. Front Microbiol 2011;2:247. doi: 10.3389/fmicb.2011.00247 [published Online First: 20111216]
193. Hassan ST, Mohamed AF, AbdelAllah NH, et al. Evaluation of MMR live attenuated vaccine oncolytic potential using Ehrlich ascites carcinoma in a murine model. Med Oncol 2022;40(1):6. doi: 10.1007/s12032-022-01866-x [published Online First: 20221029]
194. Hathaway LJ, Obeid OE, Steward MW. Protection against measles virus-induced encephalitis by antibodies from mice immunized intranasally with a synthetic peptide immunogen. Vaccine 1998;16(2-3):135-41. doi: 10.1016/s0264-410x(97)88326-0
195. Heidmeier S, Hanauer JR, Friedrich K, et al. A single amino acid substitution in the measles virus F_2 protein reciprocally modulates membrane fusion activity in pathogenic and oncolytic strains. Virus Res 2014;180:43-8. doi: 10.1016/j.virusres.2013.12.016 [published Online First: 20131222]
196. Hendricks M. Stories of survival. Johns Hopkins Mag 1995;47(1):56-62.
197. Hendrie TA, Peterson PE, Short JJ, et al. Frequency of prenatal loss in a macaque breeding colony. Am J Primatol 1996;40(1):41-53. doi: 10.1002/(sici)1098-2345(1996)40:1<41::Aid-ajp3>3.0.Co;2-0
198. Henry JE. A BRIEF STATISTICAL STUDY OF RECENT EXPERIENCE WITH MEASLES AND WHOOPING COUGH IN MASSACHUSETTS. Am J Public Health (N Y) 1921;11(4):302-6. doi: 10.2105/ajph.11.4.302
199. Herlihy JM, D'Acremont V, Hay Burgess DC, et al. Diagnosis and Treatment of the Febrile Child. In: Black RE, Laxminarayan R, Temmerman M, et al., eds. Reproductive, Maternal, Newborn, and Child Health: Disease Control Priorities, Third Edition (Volume 2). Washington (DC): The International Bank for Reconstruction and Development / The World Bank
© 2016 International Bank for Reconstruction and Development / The World Bank. 2016.
200. Hershey CL, Doocy S, Anderson J, et al. Incidence and risk factors for Malaria, pneumonia and diarrhea in children under 5 in UNHCR refugee camps: A retrospective study. Confl Health 2011;5(1):24. doi: 10.1186/1752-1505-5-24 [published Online First: 20111026]
201. Hill DR. Vaccination in Travelers. Curr Infect Dis Rep 1999;1(5):417-26. doi: 10.1007/s11908-999-0053-z
202. Hime JM, Keymer IF, Baxter CJ. Measles in recently imported colobus monkeys (Colobus guereza). Vet Rec 1975;97(20):392. doi: 10.1136/vr.97.20.392-a
203. Hodes RM, Kloos H. Health and medical care in Ethiopia. N Engl J Med 1988;319(14):918-24. doi: 10.1056/nejm198810063191406
204. Hodzic E, Hasbun R, Granillo A, et al. Steroids for the treatment of viral encephalitis: a systematic literature review and meta-analysis. J Neurol 2023:1-13. doi: 10.1007/s00415-023-11715-0 [published Online First: 20230415]
205. Hollmann C, Werner S, Avota E, et al. Inhibition of Acid Sphingomyelinase Allows for Selective Targeting of CD4+ Conventional versus Foxp3+ Regulatory T Cells. J Immunol 2016;197(8):3130-41. doi: 10.4049/jimmunol.1600691 [published Online First: 20160916]
206. Hornshøj L, Benn CS, Fernandes M, et al. Vaccination coverage and out-of-sequence vaccinations in rural Guinea-Bissau: an observational cohort study. BMJ Open 2012;2(6) doi: 10.1136/bmjopen-2012-001509 [published Online First: 20121119]
207. Hosoya M, Matsuyama S, Baba M, et al. Effects of protease inhibitors on replication of various myxoviruses. Antimicrob Agents Chemother 1992;36(7):1432-6. doi: 10.1128/aac.36.7.1432
208. Hosseinalipour SA, Mohammadbeigi A, Mohebi S, et al. Measles Elimination Successful and Its Relevant Challenges in Iran. Int J Prev Med 2022;13:52. doi: 10.4103/ijpvm.IJPVM_334_20 [published Online First: 20220405]
209. Houweling TA, Kunst AE, Mackenbach JP. Measuring health inequality among children in developing countries: does the choice of the indicator of economic status matter? Int J Equity Health 2003;2(1):8. doi: 10.1186/1475-9276-2-8 [published Online First: 20031009]
210. Hussein SE. The pattern of commoner health problems among basic school children, gezira state, Sudan. J Family Community Med 2005;12(1):27-33.
211. Hussey G. Preventing measles deaths. Child Health Dialogue 1996(3-4):14.
212. Hörner C, Fiedler AH, Bodmer BS, et al. A protective measles virus-derived vaccine inducing long-lasting immune responses against influenza A virus H7N9. NPJ Vaccines 2023;8(1):46. doi: 10.1038/s41541-023-00643-9 [published Online First: 20230324]
213. Haaga JG. How is birthspacing related to infant health? Malays J Reprod Health 1988;6(2):108-20.
214. Iankov ID, Kurokawa C, Viker K, et al. Live Attenuated Measles Virus Vaccine Expressing Helicobacter pylori Heat Shock Protein A. Mol Ther Oncolytics 2020;19:136-48. doi: 10.1016/j.omto.2020.09.006 [published Online First: 20200923]
215. Indwar P, Debnath F, Sinha A. Reporting measles case fatality due to complications from a tertiary care hospital of Kolkata, West Bengal 2011-2013. J Family Med Prim Care 2016;5(4):777-79. doi: 10.4103/2249-4863.201161
216. Institute of Medicine Committee to Study Priorities for Vaccine D. The National Academies Collection: Reports funded by National Institutes of Health. In: Stratton KR, Durch JS, Lawrence RS, eds. Vaccines for the 21st Century: A Tool for Decisionmaking. Washington (DC): National Academies Press (US)
Copyright 2000 by the National Academy of Sciences. All rights reserved. 2000.

217. Isere EE, Fatiregun AA. Measles case-based surveillance and outbreak response in Nigeria; an update for clinicians and public health professionals. Ann Ib Postgrad Med 2014;12(1):15-21.
218. Ismail T, Ahmad S, D'Souza-Ault M, et al. Cloning and expression of the nucleocapsid gene of virulent Kabete O strain of rinderpest virus in baculovirus: use in differential diagnosis between vaccinated and infected animals. Virology 1994;198(1):138-47. doi: 10.1006/viro.1994.1016
219. Jachowicz E, Gębicka M, Plakhtyr D, et al. Incidence of Vaccine-Preventable Childhood Diseases in the European Union and in the European Free Trade Association Countries. Vaccines (Basel) 2021;9(7) doi: 10.3390/vaccines9070796 [published Online First: 20210717]
220. Jacob D, Ruffie C, Dubois M, et al. Whole Pichia pastoris yeast expressing measles virus nucleoprotein as a production and delivery system to multimerize Plasmodium antigens. PLoS One 2014;9(1):e86658. doi: 10.1371/journal.pone.0086658 [published Online First: 20140127]
221. Jaupart P, Dipple L, Dercon S. Has Gavi lived up to its promise? Quasi-experimental evidence on country immunisation rates and child mortality. BMJ Glob Health 2019;4(6):e001789. doi: 10.1136/bmjgh-2019-001789 [published Online First: 20191203]
222. Jeulin H, Grancher N, Kedzierewicz F, et al. In vivo antiviral activity of ribavirin/alpha-cyclodextrin complex: evaluation on experimental measles virus encephalitis in mice. Int J Pharm 2008;357(1-2):148-53. doi: 10.1016/j.ijpharm.2008.01.043 [published Online First: 20080203]
223. Jing Y, Zaias J, Duncan R, et al. In vivo safety, biodistribution and antitumor effects of uPAR retargeted oncolytic measles virus in syngeneic cancer models. Gene Ther 2014;21(3):289-97. doi: 10.1038/gt.2013.84 [published Online First: 20140116]
224. Jones L, Giavedoni L, Saliki JT, et al. Protection of goats against peste des petits ruminants with a vaccinia virus double recombinant expressing the F and H genes of rinderpest virus. Vaccine 1993;11(9):961-4. doi: 10.1016/0264-410x(93)90386-c
225. Jordan PC, Liu C, Raynaud P, et al. Initiation, extension, and termination of RNA synthesis by a paramyxovirus polymerase. PLoS Pathog 2018;14(2):e1006889. doi: 10.1371/journal.ppat.1006889 [published Online First: 20180209]
226. Joshi M, Puvar A, Kumar D, et al. Genomic Variations in SARS-CoV-2 Genomes From Gujarat: Underlying Role of Variants in Disease Epidemiology. Front Genet 2021;12:586569. doi: 10.3389/fgene.2021.586569 [published Online First: 20210319]
227. Kadri SM, Rehman SU, Rehana K, et al. Should Mumps Be Higher Up on the Public Health Agenda in India? A Concern for Global Health Security. Med Sci (Basel) 2018;6(3) doi: 10.3390/medsci6030062 [published Online First: 20180807]
228. Kalinina LI, Stepanova LG, Rozina EE, et al. [Preparation and characteristics of diploid cell strains from the tissues of a bovine embryo]. Vopr Virusol 1979(2):152-6.
229. Kamatsuchi M, Gheorghe A, Balabanova D. The global scale and implications of delivering multiple interventions through integrated child health events. BMJ Glob Health 2019;4(4):e001333. doi: 10.1136/bmjgh-2018-001333 [published Online First: 20190708]
230. Kandadai RM, Yada P, Uppin MS, et al. Fulminant subacute sclerosing panencephalitis presenting with acute ataxia and hemiparesis in a 15-year-old boy. J Clin Neurol 2014;10(4):354-7. doi: 10.3988/jcn.2014.10.4.354 [published Online First: 20141006]
231. Kane H. Immunizations climb, then falter. World Watch 1994;7(3):33-4.
232. Karaoğlu SA, Taş BG, Toprak D. Adult vaccine-related knowledge, attitudes, and behaviors in Turkey. Clin Exp Vaccine Res 2022;11(2):133-40. doi: 10.7774/cevr.2022.11.2.133 [published Online First: 20220531]
233. Kaur T, Singh J, Tong S, et al. Descriptive epidemiology of fatal respiratory outbreaks and detection of a human-related metapneumovirus in wild chimpanzees (Pan troglodytes) at Mahale Mountains National Park, Western Tanzania. Am J Primatol 2008;70(8):755-65. doi: 10.1002/ajp.20565
234. Kelly MG, Murphy S, Elborn JS. Bronchiectasis in secondary care: a comprehensive profile of a neglected disease. Eur J Intern Med 2003;14(8):488-92. doi: 10.1016/j.ejim.2003.10.002
235. Kershaw T, Suttorp V, Simmonds K, et al. Outbreak of measles in a non-immunizing population, Alberta 2013. Can Commun Dis Rep 2014;40(12):243-50. doi: 10.14745/ccdr.v40i12a04 [published Online First: 20140612]
236. Keuzeta JJ, Merlin M, Josse R, et al. [Infant and child morbidity and mortality due to diarrheal disease in central Africa]. Ann IFORD 1988;12(1):69-87.
237. Khan A, Khan S, Ullah I, et al. Evaluation of Immunization Coverage in the Rural Area of Peshawar, Khyber Pakhtunkhwa. Cureus 2019;11(1):e3992. doi: 10.7759/cureus.3992 [published Online First: 20190131]
238. Khan MS. Paid family leave and children health outcomes in OECD countries. Child Youth Serv Rev 2020;116:105259. doi: 10.1016/j.childyouth.2020.105259 [published Online First: 20200718]
239. Khatami M. Cancer; an induced disease of twentieth century! Induction of tolerance, increased entropy and 'Dark Energy': loss of biorhythms (Anabolism v. Catabolism). Clin Transl Med 2018;7(1):20. doi: 10.1186/s40169-018-0193-6 [published Online First: 20180702]
240. Kim D, Martinez-Sobrido L, Choi C, et al. Induction of type I interferon secretion through recombinant Newcastle disease virus expressing measles virus hemagglutinin stimulates antibody secretion in the presence of maternal antibodies. J Virol 2011;85(1):200-7. doi: 10.1128/jvi.01624-10 [published Online First: 20101020]
241. Kim DJ, Yoo HS, Lee H. [Effects of the periodical spread of rinderpest on famine, epidemic, and tiger disasters in the late 17th Century]. Uisahak 2014;23(1):1-56. doi: 10.13081/kjmh.2014.23.1
242. Kim MY, Ma Y, Zhang Y, et al. hsp70-dependent antiviral immunity against cytopathic neuronal infection by vesicular stomatitis virus. J Virol 2013;87(19):10668-78. doi: 10.1128/jvi.00872-13 [published Online First: 20130724]
243. Kim MY, Shu Y, Carsillo T, et al. hsp70 and a novel axis of type I interferon-dependent antiviral immunity in the measles virus-infected brain. J Virol 2013;87(2):998-1009. doi: 10.1128/jvi.02710-12 [published Online First: 20121107]

244. Kleinlützum D, Hanauer JDS, Muik A, et al. Enhancing the Oncolytic Activity of CD133-Targeted Measles Virus: Receptor Extension or Chimerism with Vesicular Stomatitis Virus Are Most Effective. Front Oncol 2017;7:127. doi: 10.3389/fonc.2017.00127 [published Online First: 20170626]
245. Klosko RC, Lynch SE, Cabral DL, et al. Death and Disability Reported with Cases of Vaccine Anaphylaxis Stratified by Administration Setting: An Analysis of the Vaccine Adverse Event Reporting System from 2017 to 2022. Vaccines (Basel) 2023;11(2) doi: 10.3390/vaccines11020276 [published Online First: 20230128]
246. Koch A, Krönert C, Lotti T, et al. Adult Measles - Case Reports of a Highly Contagious Disease. Open Access Maced J Med Sci 2019;7(18):3009-12. doi: 10.3889/oamjms.2019.540 [published Online First: 20190630]
247. Kolla E, Weill A, Desplas D, et al. Does Measles, Mumps, and Rubella (MMR) Vaccination Protect against COVID-19 Outcomes: A Nationwide Cohort Study. Vaccines (Basel) 2022;10(11) doi: 10.3390/vaccines10111938 [published Online First: 20221116]
248. Kondamudi NP, Waymack JR. Measles. StatPearls. Treasure Island (FL): StatPearls Publishing
Copyright © 2023, StatPearls Publishing LLC. 2023.
249. Kouadio IK, Kamigaki T, Oshitani H. Measles outbreaks in displaced populations: a review of transmission, morbidity and mortality associated factors. BMC Int Health Hum Rights 2010;10:5. doi: 10.1186/1472-698x-10-5 [published Online First: 20100319]
250. Krishnamoorthy Y, Kannusamy S, Sarveswaran G, et al. Factors related to vaccine hesitancy during the implementation of Measles-Rubella campaign 2017 in rural Puducherry-A mixed-method study. J Family Med Prim Care 2019;8(12):3962-70. doi: 10.4103/jfmpc.jfmpc_790_19 [published Online First: 20191210]
251. Krumm SA, Yan D, Hovingh ES, et al. An orally available, small-molecule polymerase inhibitor shows efficacy against a lethal morbillivirus infection in a large animal model. Sci Transl Med 2014;6(232):232ra52. doi: 10.1126/scitranslmed.3008517
252. Kuate Defo B. [Causes of infant-child mortality in Yaounde]. Ann IFORD 1988;12(2):65-95.
253. Kuate Defo B. [Causes and determinants of mortality under 2 years of age in subSaharan Africa: application of concurrent risk models]. Cah Que Demogr 1997;26(1):3-39.
254. Kupka R, Nielsen J, Nyhus Dhillon C, et al. Safety and Mortality Benefits of Delivering Vitamin A Supplementation at 6 Months of Age in Sub-Saharan Africa. Food Nutr Bull 2016;37(3):375-86. doi: 10.1177/0379572116646280 [published Online First: 20160707]
255. Kurokawa M, Ochiai H, Nagasaka K, et al. Antiviral traditional medicines against herpes simplex virus (HSV-1), poliovirus, and measles virus in vitro and their therapeutic efficacies for HSV-1 infection in mice. Antiviral Res 1993;22(2-3):175-88. doi: 10.1016/0166-3542(93)90094-y
256. Kurup D, Wirblich C, Lambert R, et al. Measles-based Zika vaccine induces long-term immunity and requires NS1 antibodies to protect the female reproductive tract. NPJ Vaccines 2022;7(1):43. doi: 10.1038/s41541-022-00464-2 [published Online First: 20220419]
257. Kuzyk MA, Burian J, Machander D, et al. An efficacious recombinant subunit vaccine against the salmonid rickettsial pathogen Piscirickettsia salmonis. Vaccine 2001;19(17-19):2337-44. doi: 10.1016/s0264-410x(00)00524-7
258. Lal G, Rajala MS. Combination of Oncolytic Measles Virus Armed With BNiP3, a Pro-apoptotic Gene and Paclitaxel Induces Breast Cancer Cell Death. Front Oncol 2018;8:676. doi: 10.3389/fonc.2018.00676 [published Online First: 20190115]
259. LaMontagne DS, Cernuschi T, Yakubu A, et al. School-Based Delivery of Vaccines to 5- to 19-Year Olds. In: Bundy DAP, Silva ND, Horton S, et al., eds. Child and Adolescent Health and Development. Washington (DC): The International Bank for Reconstruction and Development / The World Bank
© 2017 International Bank for Reconstruction and Development / The World Bank. 2017.
260. Larenas-Linnemann D, Rodríguez-Pérez N, Arias-Cruz A, et al. Enhancing innate immunity against virus in times of COVID-19: Trying to untangle facts from fictions. World Allergy Organ J 2020;13(11):100476. doi: 10.1016/j.waojou.2020.100476 [published Online First: 20201009]
261. Laue T, Demir Z, Debray D, et al. Under-Vaccination in Pediatric Liver Transplant Candidates with Acute and Chronic Liver Disease-A Retrospective Observational Study of the European Reference Network TransplantChild. Children (Basel) 2021;8(8) doi: 10.3390/children8080675 [published Online First: 20210803]
262. Laure B, Jacques F, Mathilde P, et al. A Major Regional Measles Outbreak: Description of Hospitalized Cases in 2017-2018 at Bordeaux University Hospital, France. Open Forum Infect Dis 2020;7(9):ofaa332. doi: 10.1093/ofid/ofaa332 [published Online First: 20200817]
263. Lee JH, Lee K, Lee MH, et al. Wound Healing Effects of Prunus yedoensis Matsumura Bark in Scalded Rats. Evid Based Complement Alternat Med 2017;2017:7812598. doi: 10.1155/2017/7812598 [published Online First: 20170319]
264. Lehrer S, Green S, Rendo A, et al. Measles may be a Risk Factor for Malignant Brain Tumors. Brain Tumor Res Treat 2015;3(2):65-7. doi: 10.14791/btrt.2015.3.2.65 [published Online First: 20151030]
265. Lenters L, Wazny K, Bhutta ZA. Management of Severe and Moderate Acute Malnutrition in Children. In: Black RE, Laxminarayan R, Temmerman M, et al., eds. Reproductive, Maternal, Newborn, and Child Health: Disease Control Priorities, Third Edition (Volume 2). Washington (DC): The International Bank for Reconstruction and Development / The World Bank
© 2016 International Bank for Reconstruction and Development / The World Bank. 2016.
266. Leslie DL, Kobre RA, Richmond BJ, et al. Temporal Association of Certain Neuropsychiatric Disorders Following Vaccination of Children and Adolescents: A Pilot Case-Control Study. Front Psychiatry 2017;8:3. doi: 10.3389/fpsyt.2017.00003 [published Online First: 20170119]
267. Levy BM, Mirkovic RR. An epizootic of measles in a marmoset colony. Lab Anim Sci 1971;21(1):33-9.
268. Lieberherr C, Zhang G, Grafen A, et al. The Plant-Derived Naphthoquinone Droserone Inhibits In Vitro Measles Virus Infection. Planta Med 2017;83(3-04):232-38. doi: 10.1055/s-0042-111825 [published Online First: 20160715]

269. Liess B, Frey HR, Zaghawa A, et al. Morbillivirus infection of seals (Phoca vitulina) during the 1988 epidemic in the Bay of Heligoland. I. Mode, frequency and significance of cultural virus isolation and neutralizing antibody detection. Zentralbl Veterinarmed B 1989;36(8):601-8. doi: 10.1111/j.1439-0450.1989.tb00651.x
270. Lin LT, Richardson CD. The Host Cell Receptors for Measles Virus and Their Interaction with the Viral Hemagglutinin (H) Protein. Viruses 2016;8(9) doi: 10.3390/v8090250 [published Online First: 20160920]
271. Lin WH, Griffin DE, Rota PA, et al. Successful respiratory immunization with dry powder live-attenuated measles virus vaccine in rhesus macaques. Proc Natl Acad Sci U S A 2011;108(7):2987-92. doi: 10.1073/pnas.1017334108 [published Online First: 20110131]
272. Lin WW, Moran E, Adams RJ, et al. A durable protective immune response to wild-type measles virus infection of macaques is due to viral replication and spread in lymphoid tissues. Sci Transl Med 2020;12(537) doi: 10.1126/scitranslmed.aax7799
273. Lin WW, Tsay AJ, Lalime EN, et al. Primary differentiated respiratory epithelial cells respond to apical measles virus infection by shedding multinucleated giant cells. Proc Natl Acad Sci U S A 2021;118(11) doi: 10.1073/pnas.2013264118
274. Lin Z, Huang W, Xie Z, et al. Expression, Clinical Significance, Immune Infiltration, and Regulation Network of miR-3940-5p in Lung Adenocarcinoma Based on Bioinformatic Analysis and Experimental Validation. Int J Gen Med 2022;15:6451-64. doi: 10.2147/ijgm.S375761 [published Online First: 20220806]
275. Litwińska B, Bucholc B, Sadowski W, et al. [Antiviral activity of commercial immunoglobulin preparations]. Med Dosw Mikrobiol 1990;42(1-2):89-94.
276. Liu L, Li M, Cummings S, et al. Deriving causes of child mortality by re-analyzing national verbal autopsy data applying a standardized computer algorithm in Uganda, Rwanda and Ghana. J Glob Health 2015;5(1):010414. doi: 10.7189/jogh.05.010414
277. Liu L, Li Q, Lee RA, et al. Trends in causes of death among children under 5 in Bangladesh, 1993-2004: an exercise applying a standardized computer algorithm to assign causes of death using verbal autopsy data. Popul Health Metr 2011;9:43. doi: 10.1186/1478-7954-9-43 [published Online First: 20110805]
278. Lo MK, Jordan PC, Stevens S, et al. Susceptibility of paramyxoviruses and filoviruses to inhibition by 2'-monofluoro- and 2'-difluoro-4'-azidocytidine analogs. Antiviral Res 2018;153:101-13. doi: 10.1016/j.antiviral.2018.03.009 [published Online First: 20180327]
279. Lobl TJ, Renis HE, Epand RM, et al. Peptides as potential virus inhibitors. Synthesis and bioassay of five respiratory syncytial virus peptide analogs with antimeasles activity. Int J Pept Protein Res 1988;32(5):326-30.
280. Lorin C, Segal L, Mols J, et al. Toxicology, biodistribution and shedding profile of a recombinant measles vaccine vector expressing HIV-1 antigens, in cynomolgus macaques. Naunyn Schmiedebergs Arch Pharmacol 2012;385(12):1211-25. doi: 10.1007/s00210-012-0793-4 [published Online First: 20120916]
281. Low C, Thoon KC, Lin R, et al. Possible nosocomial transmission of measles in unvaccinated children in a Singapore public hospital. Western Pac Surveill Response J 2012;3(4):7-11. doi: 10.5365/wpsar.2012.3.4.008 [published Online First: 20121030]
282. Ludlow M, de Vries RD, Lemon K, et al. Infection of lymphoid tissues in the macaque upper respiratory tract contributes to the emergence of transmissible measles virus. J Gen Virol 2013;94(Pt 9):1933-44. doi: 10.1099/vir.0.054650-0 [published Online First: 20130619]
283. Madhu K, Chowdary S, Masthi R. Breast feeding practices and newborn care in rural areas: a descriptive cross-sectional study. Indian J Community Med 2009;34(3):243-6. doi: 10.4103/0970-0218.55292
284. Magno H, Golomb B. Measuring the Benefits of Mass Vaccination Programs in the United States. Vaccines (Basel) 2020;8(4) doi: 10.3390/vaccines8040561 [published Online First: 20200929]
285. Măgureanu E, Feller H. [Contributions to the study of the historical evolution of some infectious diseases in Rumania. II. Measles (1898-1964)]. Arch Roum Pathol Exp Microbiol 1965;24(4):959-78.
286. Majumder M, Cusick MM, Rose S. Data Source Concordance for Infectious Disease Epidemiology. medRxiv 2022 doi: 10.1101/2022.06.02.22275926 [published Online First: 20220603]
287. Makhortova NR, Askovich P, Patterson CE, et al. Neurokinin-1 enables measles virus trans-synaptic spread in neurons. Virology 2007;362(1):235-44. doi: 10.1016/j.virol.2007.02.033 [published Online First: 20070416]
288. Makhtar ST, Tan SW, Nasruddin NA, et al. Development of TaqMan-based real-time RT-PCR assay based on N gene for the quantitative detection of feline morbillivirus. BMC Vet Res 2021;17(1):128. doi: 10.1186/s12917-021-02837-6 [published Online First: 20210323]
289. Maldonado YA. Lessons From a House on Fire-From Smallpox to Polio. J Infect Dis 2023;227(9):1025-27. doi: 10.1093/infdis/jiad017
290. Malik B, Sharma FJ, Bhardwaj AK, et al. Sub acute sclerosing pan encephalitis despite adequate vaccination. Australas Med J 2012;5(7):359-61. doi: 10.4066/amj.2012.1262 [published Online First: 20120731]
291. Malik JA, Agrewala JN. Future perspectives of emerging novel drug targets and immunotherapies to control drug addiction. Int Immunopharmacol 2023;119:110210. doi: 10.1016/j.intimp.2023.110210 [published Online First: 20230424]
292. Mancini S, Coldiron ME, Ronsse A, et al. Description of a large measles epidemic in Democratic Republic of Congo, 2010-2013. Confl Health 2014;8:9. doi: 10.1186/1752-1505-8-9 [published Online First: 20140703]
293. Mangla A, Agarwal N. Clinical Practice Issues In American Indians and Alaska Natives. StatPearls. Treasure Island (FL): StatPearls Publishing
Copyright © 2023, StatPearls Publishing LLC. 2023.
294. Maple PAC. Population (Antibody) Testing for COVID-19-Technical Challenges, Application and Relevance, an English Perspective. Vaccines (Basel) 2021;9(6) doi: 10.3390/vaccines9060550 [published Online First: 20210524]
295. Marie JC, Kehren J, Trescol-Biémont MC, et al. Mechanism of measles virus-induced suppression of inflammatory immune responses. Immunity 2001;14(1):69-79. doi: 10.1016/s1074-7613(01)00090-5

296. Markushin SG, Litvin AA. [Antiviral activity of panavir in experimental Measles virus infection in cell cultures]. Antibiot Khimioter 2009;54(1-2):14-6.
297. Matsuo N, Tomono K, Matsuse H, et al. Diagnosis of Measles Pneumonia from Bronchoalveolar Lavage Fluid by Reverse-Transcriptase Polymerase Chain Reaction: Case Report. J Infect Chemother 1997;3(4):212-15. doi: 10.1007/bf02490038 [published Online First: 20140405]
298. Mbkikusita-lewanika I. The state of the art of education for child survival and development in Kenya. BERC Bull 1987(15):12-5.
299. Mbodj FG. [Malaria and diarrhea, principal causes of infant deaths]. Pop Sahel 1989(10):10-1.
300. McLagan D. Roskens, Sullivan lead presidential mission to Africa. Front Lines 1991:3-5.
301. Medlin CA, Chowdhury M, Jamison DT, et al. Improving the Health of Populations: Lessons of Experience. In: Jamison DT, Breman JG, Measham AR, et al., eds. Disease Control Priorities in Developing Countries. Washington (DC)
New York: The International Bank for Reconstruction and Development / The World Bank
Oxford University Press
Copyright © 2006, The International Bank for Reconstruction and Development/The World Bank Group. 2006.
302. Mehlman MJ, Lederman MM. Compulsory Immunization Protects Against Infection: What Law and Society Can Do. Pathog Immun 2020;5(1):1-7. doi: 10.20411/pai.v5i1.344 [published Online First: 20200120]
303. Meireles BC, Goldschmidt B, Resende FC, et al. Neotropical primate nursery in a squirrel monkey breeding unit in Brazil. Lab Anim 2016;50(1):67-72. doi: 10.1177/0023677215570991 [published Online First: 20150204]
304. Merler S, Ajelli M. Deciphering the relative weights of demographic transition and vaccination in the decrease of measles incidence in Italy. Proc Biol Sci 2014;281(1777):20132676. doi: 10.1098/rspb.2013.2676 [published Online First: 20140108]
305. Miller MA, Sentz JT. Vaccine-Preventable Diseases. In: Jamison DT, Feachem RG, Makgoba MW, et al., eds. Disease and Mortality in Sub-Saharan Africa. Washington (DC): The International Bank for Reconstruction and Development / The World Bank
Copyright © 2006, The International Bank for Reconstruction and Development/The World Bank. 2006.
306. Minor PD. The perilous path to a better measles vaccine. CVI Forum 1992(2):8-10.
307. Mishra A, Mishra S, Lahariya C, et al. Practical observations from an epidemiological investigation of a measles outbreak in a district of India. Indian J Community Med 2009;34(2):117-21. doi: 10.4103/0970-0218.51234
308. Moghadam M, Afsarkazerooni P, Ebrahimi M, et al. Measles outbreak in South of iran, where vaccine coverage was high: a case-series study. Iran J Public Health 2014;43(3):375-80.
309. Mohammadbeigi A, Mokhtari M, Zahraei SM, et al. Survival Analysis for Predictive Factors of Delay Vaccination in Iranian Children. Int J Prev Med 2015;6:119. doi: 10.4103/2008-7802.170868 [published Online First: 20151201]
310. Moon SM, Choi H, Kang HK, et al. Impacts of Asthma in Patients With Bronchiectasis: Findings From the KMBARC Registry. Allergy Asthma Immunol Res 2023;15(1):83-93. doi: 10.4168/aair.2023.15.1.83
311. Morgan OW. Following in the footsteps of smallpox: can we achieve the global eradication of measles? BMC Int Health Hum Rights 2004;4(1):1. doi: 10.1186/1472-698x-4-1 [published Online First: 20040317]
312. Morkuniene R, Zvirbliene A, Dalgediene I, et al. Antibodies bound to Aβ oligomers potentiate the neurotoxicity of Aβ by activating microglia. J Neurochem 2013;126(5):604-15. doi: 10.1111/jnc.12332 [published Online First: 20130627]
313. Morris SE, Yates AJ, de Swart RL, et al. Modeling the measles paradox reveals the importance of cellular immunity in regulating viral clearance. PLoS Pathog 2018;14(12):e1007493. doi: 10.1371/journal.ppat.1007493 [published Online First: 20181228]
314. Morris SE, Zelner JL, Fauquier DA, et al. Partially observed epidemics in wildlife hosts: modelling an outbreak of dolphin morbillivirus in the northwestern Atlantic, June 2013-2014. J R Soc Interface 2015;12(112) doi: 10.1098/rsif.2015.0676
315. Muench F, Krusche M, Sander LE, et al. Macrophage activation syndrome in a patient with adult-onset Still's disease following first COVID-19 vaccination with BNT162b2. BMC Rheumatol 2021;5(1):60. doi: 10.1186/s41927-021-00237-9 [published Online First: 20211228]
316. Mukherjee S. A new thrust and dynamism. India. Integration 1992(33):41-3.
317. Muluneh F, Wubetu M, Abate A. Missed Opportunity for Routine Immunization and Its Associated Factors in Gozamen District Health Centers, Northwestern Ethiopia. Glob Pediatr Health 2020;7:2333794x20981306. doi: 10.1177/2333794x20981306 [published Online First: 20201220]
318. Muñoz-Alía M, Nace RA, Balakrishnan B, et al. Surface-modified measles vaccines encoding oligomeric, fusion-stabilized SARS-CoV-2 spike glycoproteins bypass measles seropositivity, boosting neutralizing antibody responses to omicron and historical variants. bioRxiv 2022 doi: 10.1101/2022.12.16.520799 [published Online First: 20221216]
319. Munro R, Ross H, Cornwell C, et al. Disease conditions affecting common seals (Phoca vitulina) around the Scottish mainland, September-November 1988. Sci Total Environ 1992;115(1-2):67-82. doi: 10.1016/0048-9697(92)90033-o
320. Mutua MK, Kimani-Murage E, Ngomi N, et al. Fully immunized child: coverage, timing and sequencing of routine immunization in an urban poor settlement in Nairobi, Kenya. Trop Med Health 2016;44:13. doi: 10.1186/s41182-016-0013-x [published Online First: 20160516]
321. Mutunga SN. Some cultural constraints which affect child survival and development in Kenya with reference to Kwale district. BERC Bull 1986(14):16-20.
322. Myint NW, Kaewkungwal J, Singhasivanon P, et al. Are there any changes in burden and management of communicable diseases in areas affected by Cyclone Nargis? Confl Health 2011;5(1):9. doi: 10.1186/1752-1505-5-9 [published Online First: 20110628]
323. Mysore V, Cullere X, Settles ML, et al. Protective heterologous T cell immunity in COVID-19 induced by MMR and Tdap vaccine antigens. bioRxiv 2021 doi: 10.1101/2021.05.03.441323 [published Online First: 20210504]

324. Nagalo BM, Breton CA, Zhou Y, et al. Oncolytic Virus with Attributes of Vesicular Stomatitis Virus and Measles Virus in Hepatobiliary and Pancreatic Cancers. Mol Ther Oncolytics 2020;18:546-55. doi: 10.1016/j.omto.2020.08.007 [published Online First: 20200819]
325. Nakatsu Y, Takeda M, Ohno S, et al. Measles virus circumvents the host interferon response by different actions of the C and V proteins. J Virol 2008;82(17):8296-306. doi: 10.1128/jvi.00108-08 [published Online First: 20080618]
326. Nana HM, Ngane RA, Kuiate JR, et al. Acute and sub-acute toxicity of the methanolic extract of Pteleopsis hylodendron stem bark. J Ethnopharmacol 2011;137(1):70-6. doi: 10.1016/j.jep.2011.04.032 [published Online First: 20110530]
327. Ndungu JM, Krumm SA, Yan D, et al. Non-nucleoside inhibitors of the measles virus RNA-dependent RNA polymerase: synthesis, structure-activity relationships, and pharmacokinetics. J Med Chem 2012;55(9):4220-30. doi: 10.1021/jm201699w [published Online First: 20120420]
328. Newport MJ. The genetic regulation of infant immune responses to vaccination. Front Immunol 2015;6:18. doi: 10.3389/fimmu.2015.00018 [published Online First: 20150202]
329. Nguyen SN, Vu LT, Vu QV, et al. Clinical Epidemiology Characteristics and Etiology of Febrile Neutropenia in Children: Analysis of 421 Cases. Hematol Rep 2022;14(3):245-52. doi: 10.3390/hematolrep14030034 [published Online First: 20220801]
330. Nieburg P, Berry AM, Steketee RW, et al. Limitations of anthropometry during acute food shortages: high mortality can mask refugees' deteriorating nutritional status. Disasters 1988;12(3):253-8. doi: 10.1111/j.1467-7717.1988.tb00674.x
331. Nielsen S, Fisker AB, da Silva I, et al. Effect of early two-dose measles vaccination on childhood mortality and modification by maternal measles antibody in Guinea-Bissau, West Africa: A single-centre open-label randomised controlled trial. EClinicalMedicine 2022;49:101467. doi: 10.1016/j.eclinm.2022.101467 [published Online First: 20220527]
332. Nielsen S, Khalek MA, Benn CS, et al. National immunisation campaigns with oral polio vaccine may reduce all-cause mortality: Analysis of 2004-2019 demographic surveillance data in rural Bangladesh. EClinicalMedicine 2021;36:100886. doi: 10.1016/j.eclinm.2021.100886 [published Online First: 20210524]
333. Nielsen S, Sujan HM, Benn CS, et al. Oral Polio Vaccine Campaigns May Reduce the Risk of Death from Respiratory Infections. Vaccines (Basel) 2021;9(10) doi: 10.3390/vaccines9101133 [published Online First: 20211004]
334. Ntilivamunda A. [Program to combat communicable diseases in children]. Imbonezamuryango 1985(3):50-2.
335. Ntilivamunda A. [Expanded Programme on Immunization: status and perspectives]. Imbonezamuryango 1988(13):29-34.
336. Obeid OE, Partidos CD, Howard CR, et al. Protection against morbillivirus-induced encephalitis by immunization with a rationally designed synthetic peptide vaccine containing B- and T-cell epitopes from the fusion protein of measles virus. J Virol 1995;69(3):1420-8. doi: 10.1128/jvi.69.3.1420-1428.1995
337. Obeid OE, Stanley CM, Steward MW. Immunological analysis of the protective responses to the chimeric synthetic peptide representing T- and B-cell epitopes from the fusion protein of measles virus. Virus Res 1996;42(1-2):173-80. doi: 10.1016/0168-1702(96)01311-1
338. Obeid OE, Steward MW. The potential of immunization with synthetic peptides to overcome the immunosuppressive effect of maternal anti-measles virus antibodies in young mice. Immunology 1994;82(1):16-21.
339. Ogbu TJ, Scales SE, de Almeida MM, et al. Predictors of exceeding emergency under-five mortality thresholds using small-scale survey data from humanitarian settings (1999 - 2020): considerations for measles vaccination, malnutrition, and displacement status. Arch Public Health 2022;80(1):160. doi: 10.1186/s13690-022-00916-0 [published Online First: 20220628]
340. Ogimi C, Qu P, Boeckh M, et al. Association between live childhood vaccines and COVID-19 outcomes: a national-level analysis. medRxiv 2020 doi: 10.1101/2020.10.17.20214510 [published Online First: 20201020]
341. Okello D, Yang S, Komakech R, et al. An in vitro Propagation of Aspilia africana (Pers.) C. D. Adams, and Evaluation of Its Anatomy and Physiology of Acclimatized Plants. Front Plant Sci 2021;12:704896. doi: 10.3389/fpls.2021.704896 [published Online First: 20210729]
342. Olaitan AE, Ella EE, Ameh JB. Comparative seroprevalence of measles virus immunoglobulin M antibodies in children aged 0-8 months and a control population aged 9-23 months presenting with measles-like symptoms in selected hospitals in Kaduna State. Int J Gen Med 2015;8:101-8. doi: 10.2147/ijgm.S79423 [published Online First: 20150310]
343. Olshansky SJ, Hayflick L. The Role of the WI-38 Cell Strain in Saving Lives and Reducing Morbidity. AIMS Public Health 2017;4(2):127-38. doi: 10.3934/publichealth.2017.2.127 [published Online First: 20170302]
344. Olsson T, Maehlen J, Löve A, et al. Induction of class I and class II transplantation antigens in rat brain during fatal and non-fatal measles virus infection. J Neuroimmunol 1987;16(2):215-24. doi: 10.1016/0165-5728(87)90076-2
345. Olszewska W, Steward MW. The molecular basis of the antigenic cross-reactivity between measles and cowpea mosaic viruses. Virology 2003;310(1):183-9. doi: 10.1016/s0042-6822(03)00118-1
346. Onah S, Osuorah DI, Ebenebe J, et al. Infant feeding practices and maternal socio-demographic factors that influence practice of exclusive breastfeeding among mothers in Nnewi South-East Nigeria: a cross-sectional and analytical study. Int Breastfeed J 2014;9:6. doi: 10.1186/1746-4358-9-6 [published Online First: 20140520]
347. Onoja AB, Hamid KM, Adeniji JA, et al. Implication of vaccination on measles reduction and elimination in Nigeria. Afr J Med Med Sci 2014;43(Suppl 1):73-78.
348. Oral HB, Akdis CA. Antibody-based therapies in infectious diseases. Methods Mol Med 2000;40:157-78. doi: 10.1385/1-59259-076-4:157
349. Oshikoya KA, Senbanjo IO. Pathophysiological changes that affect drug disposition in protein-energy malnourished children. Nutr Metab (Lond) 2009;6:50. doi: 10.1186/1743-7075-6-50 [published Online First: 20091201]
350. Otczyk DC, Cripps AW. Delivering vaccines for the prevention of pneumonia - programmatic and financial issues. Pneumonia (Nathan) 2013;2:16-25. doi: 10.15172/pneu.2013.2/244 [published Online First: 20130214]

351. Ottolini M, Cirks B, Madden KB, et al. Pediatric Infectious Diseases Encountered During Wartime-Part 1: Experiences and Lessons Learned From Armed Conflict in the Modern Era. Curr Infect Dis Rep 2021;23(12):27. doi: 10.1007/s11908-021-00770-1 [published Online First: 20211209]
352. Pacenti M, Maione N, Lavezzo E, et al. Measles Virus Infection and Immunity in a Suboptimal Vaccination Coverage Setting. Vaccines (Basel) 2019;7(4) doi: 10.3390/vaccines7040199 [published Online First: 20191128]
353. Padmanabhan BS. Child survival and development. Iccw j 1993;2(2-3):39-40.
354. Pan CH, Nair N, Adams RJ, et al. Dose-dependent protection against or exacerbation of disease by a polylactide glycolide microparticle-adsorbed, alphavirus-based measles virus DNA vaccine in rhesus macaques. Clin Vaccine Immunol 2008;15(4):697-706. doi: 10.1128/cvi.00045-08 [published Online First: 20080220]
355. Pan J, Wang Y, Cao L, et al. Impact of immunization programs on 11 childhood vaccine-preventable diseases in China: 1950-2018. Innovation (Camb) 2021;2(2):100113. doi: 10.1016/j.xinn.2021.100113 [published Online First: 20210505]
356. Park GYS, Tishkowski K. Paramyxovirus. StatPearls. Treasure Island (FL): StatPearls Publishing
Copyright © 2023, StatPearls Publishing LLC. 2023.
357. Park SH, Lee DH, Jin JY, et al. Measles outbreaks in the Kyeongin area of the Republic of Korea, 2013-2014: A single-center experience in a country of measles elimination. Asian Pac J Trop Med 2017;10(1):69-74. doi: 10.1016/j.apjtm.2016.12.003 [published Online First: 20161227]
358. Parkins MD, McNeil SA, Laupland KB. Routine immunization of adults in Canada: Review of the epidemiology of vaccine-preventable diseases and current recommendations for primary prevention. Can J Infect Dis Med Microbiol 2009;20(3):e81-90. doi: 10.1155/2009/474035
359. Partap U, Hill DR. The Maoist insurgency (1996-2006) and child health indicators in Nepal. Int Health 2012;4(2):135-42. doi: 10.1016/j.inhe.2011.12.004
360. Partidos CD, Vohra P, Steward MW. Induction of measles virus-specific cytotoxic T-cell responses after intranasal immunization with synthetic peptides. Immunology 1996;87(2):179-85. doi: 10.1046/j.1365-2567.1996.462527.x
361. Pasetti MF, Barry EM, Losonsky G, et al. Attenuated Salmonella enterica serovar Typhi and Shigella flexneri 2a strains mucosally deliver DNA vaccines encoding measles virus hemagglutinin, inducing specific immune responses and protection in cotton rats. J Virol 2003;77(9):5209-17. doi: 10.1128/jvi.77.9.5209-5217.2003
362. Peacock GV. Agents for passive immunization against canine distemper. J Am Vet Med Assoc 1966;149(5):633-9.
363. Peart Akindele NA, Katamoni LD, Brockhurst J, et al. Effect of remdesivir post-exposure prophylaxis and treatment on pathogenesis of measles in rhesus macaques. Sci Rep 2023;13(1):6463. doi: 10.1038/s41598-023-33572-7 [published Online First: 20230420]
364. Pease DF, Kratzke RA. Oncolytic Viral Therapy for Mesothelioma. Front Oncol 2017;7:179. doi: 10.3389/fonc.2017.00179 [published Online First: 20170824]
365. Pergam SA. Infection Prevention in Transplantation. Curr Infect Dis Rep 2016;18(2):7. doi: 10.1007/s11908-015-0513-6
366. Persha AJ. Early identification and prevention of mental retardation. ICCW News Bull 1992;40(1):9-12.
367. Petrack EM. Health care in Nicaragua: a social and historical perspective. N Y State J Med 1984;84(10):523-5.
368. Pfäfflin F, Flick H, Vogtmann M. [Rare viral infections of the lungs]. Pneumologe (Berl) 2020;17(5):311-21. doi: 10.1007/s10405-020-00337-2 [published Online First: 20200720]
369. Phan TT, Enlow PT, Wong MK, et al. Medical factors associated with caregiver intention to vaccinate their children against COVID-19. Vaccine X 2022;10:100144. doi: 10.1016/j.jvacx.2022.100144 [published Online First: 20220131]
370. Piccaluga PP, Malerba G, Navari M, et al. Cross-Immunization Against Respiratory Coronaviruses May Protect Children From SARS-CoV2: More Than a Simple Hypothesis? Front Pediatr 2020;8:595539. doi: 10.3389/fped.2020.595539 [published Online First: 20210118]
371. Polack FP, Lee SH, Permar S, et al. Successful DNA immunization against measles: neutralizing antibody against either the hemagglutinin or fusion glycoprotein protects rhesus macaques without evidence of atypical measles. Nat Med 2000;6(7):776-81. doi: 10.1038/77506
372. Polack FP, Lydy SL, Lee SH, et al. Poor immune responses of newborn rhesus macaques to measles virus DNA vaccines expressing the hemagglutinin and fusion glycoproteins. Clin Vaccine Immunol 2013;20(2):205-10. doi: 10.1128/cvi.00394-12 [published Online First: 20121212]
373. Polonsky JA, Ronsse A, Ciglenecki I, et al. High levels of mortality, malnutrition, and measles, among recently-displaced Somali refugees in Dagahaley camp, Dadaab refugee camp complex, Kenya, 2011. Confl Health 2013;7(1):1. doi: 10.1186/1752-1505-7-1 [published Online First: 20130122]
374. Premenko-Lanier M, Rota PA, Rhodes G, et al. DNA vaccination of infants in the presence of maternal antibody: a measles model in the primate. Virology 2003;307(1):67-75. doi: 10.1016/s0042-6822(02)00036-3
375. Premenko-Lanier M, Rota PA, Rhodes GH, et al. Protection against challenge with measles virus (MV) in infant macaques by an MV DNA vaccine administered in the presence of neutralizing antibody. J Infect Dis 2004;189(11):2064-71. doi: 10.1086/420792 [published Online First: 20040511]
376. Provost A, Maurice Y, Borredon C. [Rinderpest protection of bovines by measles virus. II. Vaccination of calves born from cows vaccinated with the MB 113 strain]. Rev Elev Med Vet Pays Trop 1971;24(2):167-72.
377. Quamruzzaman A, Lange M. Female political representation and child health: Evidence from a multilevel analysis. Soc Sci Med 2016 doi: 10.1016/j.socscimed.2016.10.025 [published Online First: 20161024]
378. Rafat C, Klouche K, Ricard JD, et al. Severe Measles Infection: The Spectrum of Disease in 36 Critically Ill Adult Patients. Medicine (Baltimore) 2013;92(5):257-72. doi: 10.1097/MD.0b013e3182a713c2
379. Raimbault AM. Voluntary workers and health improvement. Child Trop 1972;84:19-28.

380. Rajaraman S, Canjuga D, Ghosh M, et al. Measles Virus-Based Treatments Trigger a Pro-inflammatory Cascade and a Distinctive Immunopeptidome in Glioblastoma. Mol Ther Oncolytics 2019;12:147-61. doi: 10.1016/j.omto.2018.12.010 [published Online First: 20181231]
381. Ramaroson SR, Rakotomanga JD. [Causes of mortality in a rural area in the Faritany of Toamasina in 1986]. Ravintsara 1992(4):31-48.
382. Rammohan KW, McFarlin DE, McFarland HF. Chronic measles encephalitis in mice. J Infect Dis 1980;142(4):608-13. doi: 10.1093/infdis/142.4.608
383. Ravenholt A. Malnutrition in the Philippines. Am Univ Field Staff Rep Asia 1982(20):3-12.
384. Regassa N, Bird Y, Moraros J. Preference in the use of full childhood immunizations in Ethiopia: the role of maternal health services. Patient Prefer Adherence 2019;13:91-99. doi: 10.2147/ppa.S189844 [published Online First: 20190108]
385. Reinert P. [Infections in malnourished infants and children]. Dev Sante 1993(103):4-6.
386. Remfry J. A measles epizootic with 5 deaths in newly-imported rhesus monkeys (Macaca mulatta). Lab Anim 1976;10(1):49-57. doi: 10.1258/002367776780948925
387. Richard SA, Kampo S, Esquijarosa Hechavarria M. Elucidating the pivotal role of convalescent plasma therapy in critically ill COVID-19 patients: A review. Hematol Rep 2020;12(3):8630. doi: 10.4081/hr.2020.8630 [published Online First: 20201202]
388. Rima BK, Curran MD, Kennedy S. Phocine distemper virus, the agent responsible for the 1988 mass mortality of seals. Sci Total Environ 1992;115(1-2):45-55. doi: 10.1016/0048-9697(92)90031-m
389. Rincón-León HA, Navarro-Fuentes KR. [Measles: a millennial itch in the COVID-19 era]. Rev Med Inst Mex Seguro Soc 2020;58(6):644-47. doi: 10.24875/rmimss.M20000095
390. Ristori C, Boccardo H, Miranda M, et al. [VACCINATION AGAINST MEASLES WITH LIVE VIRUS FROM THE EDMONSTON STRAIN-NATIONAL EXPERIENCE]. Rev Chil Pediatr 1963;34:656-63.
391. Robinson D. The integrated management of childhood illness. Afr Health 1996;18(6):20-1.
392. Robinson E, Crispino V, Ouabo A, et al. Mortality and health survey, Walikale, Democratic Republic of the Congo, 2017: an example of the use of survey data for humanitarian program planning. Confl Health 2019;13:56. doi: 10.1186/s13031-019-0232-y [published Online First: 20191121]
393. Robinzon S, Dafa-Berger A, Dyer MD, et al. Impaired cholesterol biosynthesis in a neuronal cell line persistently infected with measles virus. J Virol 2009;83(11):5495-504. doi: 10.1128/jvi.01880-08 [published Online First: 20090318]
394. Rocke Z, Belyayeva M. Subacute Sclerosing Panencephalitis. StatPearls. Treasure Island (FL): StatPearls Publishing Copyright © 2023, StatPearls Publishing LLC. 2023.
395. Rodeheffer C, von Messling V, Milot S, et al. Disease manifestations of canine distemper virus infection in ferrets are modulated by vitamin A status. J Nutr 2007;137(8):1916-22. doi: 10.1093/jn/137.8.1916
396. Rodrigues CMC. Challenges of Empirical Antibiotic Therapy for Community-Acquired Pneumonia in Children. Curr Ther Res Clin Exp 2017;84:e7-e11. doi: 10.1016/j.curtheres.2017.01.002 [published Online First: 20170116]
397. Roggero PF, Calistri A, Palù G. On the intrinsic nature of viral pathogenesis: The assumption of a Darwinian paradigm to describe COVID-19 pandemic. Comput Struct Biotechnol J 2022;20:5870-72. doi: 10.1016/j.csbj.2022.10.037 [published Online First: 20221028]
398. Roĭtman MP. [Methods of measuring economic effectiveness in public health and some results of their use in the USSR]. Sov Zdravookhr 1975(6):8-13.
399. Roudi N. Survey report: Jordan. Popul Today 1991;19(12):4.
400. Rull M, Masson S, Peyraud N, et al. The new WHO decision-making framework on vaccine use in acute humanitarian emergencies: MSF experience in Minkaman, South Sudan. Confl Health 2018;12:11. doi: 10.1186/s13031-018-0147-z [published Online First: 20180326]
401. Sadkowska-Todys M, Zieliński A, Czarkowski M. Infectious diseases in Poland in 2015. Przegl Epidemiol 2017;71(3):295-309.
402. Sagami S, Kobayashi T, Hibi T. Prevention of Infectious Diseases due to Immunosuppression and Vaccinations in Asian Patients with Inflammatory Bowel Disease. Inflamm Intest Dis 2018;3(1):1-10. doi: 10.1159/000489643 [published Online First: 20180717]
403. Sahiledengle B, Agho K. Determinants of Childhood Diarrhea in Households with Improved Water, Sanitation, and Hygiene (WASH) in Ethiopia: Evidence from a Repeated Cross-Sectional Study. Environ Health Insights 2021;15:11786302211025180. doi: 10.1177/11786302211025180 [published Online First: 20210621]
404. Şahin K, Bayhan G, Alan B. Bulging fontanel during a measles infection. Germs 2020;10(4):385-87. doi: 10.18683/germs.2020.1231 [published Online First: 20201228]
405. Sakae H, Kohase M, Kurata T, et al. Isolation of a measles virus variant: protection of newborn mice from measles encephalitis by 24 h prior intracerebral inoculation with the variant. Arch Virol 1997;142(10):1937-52. doi: 10.1007/s007050050212
406. Salman Y, Shaeen SK, Khan HA, et al. The effect of child malnourishment on measles spread amidst the COVID-19 pandemic in Afghanistan. Ann Med Surg (Lond) 2022;78:103798. doi: 10.1016/j.amsu.2022.103798 [published Online First: 20220518]
407. Santagati NA, Bousquet E, Garozzo A, et al. Synthesis and anti-measles virus activity of new isoquinolin-4-one derivatives. Farmaco 2003;58(12):1217-25. doi: 10.1016/j.farmac.2003.07.003
408. Santos IJ, Leite GO, Costa JG, et al. Topical Anti-Inflammatory Activity of Oil from Tropidurus hispidus (Spix, 1825). Evid Based Complement Alternat Med 2015;2015:140247. doi: 10.1155/2015/140247 [published Online First: 20151118]
409. Saxena D. Measles is down but not out. Indian Med Trib 1994;2(4):1, 4.

410. Schmitz KS, Eblé PL, van Gennip RGP, et al. Pathogenesis of wild-type- and vaccine-based recombinant peste des petits ruminants virus (PPRV) expressing EGFP in experimentally infected domestic goats. J Gen Virol 2023;104(2) doi: 10.1099/jgv.0.001828
411. Schumacher HP, Albrecht P, Clark RG, et al. Intracerebral inoculation of rhesus monkeys with a strain of measles virus isolated from a case of subacute sclerosing panencephalitis. Infect Immun 1971;4(4):419-24. doi: 10.1128/iai.4.4.419-424.1971
412. Seal AJ, Mohamed HA, Stokes-Walter R, et al. Use of an adapted participatory learning and action cycle to increase knowledge and uptake of child vaccination in internally displaced persons camps (IVACS): A cluster-randomised controlled trial. Vaccine 2023 doi: 10.1016/j.vaccine.2023.02.016 [published Online First: 20230309]
413. Sell S, Guest I, McKinstry KK, et al. Intraepithelial T-cell cytotoxicity, induced bronchus-associated lymphoid tissue, and proliferation of pneumocytes in experimental mouse models of influenza. Viral Immunol 2014;27(10):484-96. doi: 10.1089/vim.2014.0077
414. Sell S, McKinstry KK, Strutt TM. Mouse Models Reveal Role of T-Cytotoxic and T-Reg Cells in Immune Response to Influenza: Implications for Vaccine Design. Viruses 2019;11(1) doi: 10.3390/v11010052 [published Online First: 20190111]
415. Sellin CI, Jégou JF, Renneson J, et al. Interplay between virus-specific effector response and Foxp3 regulatory T cells in measles virus immunopathogenesis. PLoS One 2009;4(3):e4948. doi: 10.1371/journal.pone.0004948 [published Online First: 20090325]
416. Semenov BF. Vaccinoprophylaxis on the Eve of Centuries. Russ J Immunol 1999;4(4):346-51.
417. Seo CY, Rashid M, Harris T, et al. Assessing safety of Ontario's publicly funded MMR and MMRV immunization programs, 2012 to 2016. Paediatr Child Health 2020;25(6):358-64. doi: 10.1093/pch/pxz037 [published Online First: 20190408]
418. Sesay T, Denisiuk O, Shringarpure KK, et al. Paediatric care in relation to the 2014-2015 Ebola outbreak and general reporting of deaths in Sierra Leone. Public Health Action 2017;7(Suppl 1):S34-s39. doi: 10.5588/pha.16.0088
419. Sesay T, Denisiuk O, Zachariah R. Paediatric morbidity and mortality in Sierra Leone. Have things changed after the 2014/2015 Ebola outbreak? F1000Res 2019;8:796. doi: 10.12688/f1000research.18552.2 [published Online First: 20190606]
420. Sethi GR. Environment and acute respiratory infections. ICCW News Bull 1992;40(3-4):27-9.
421. Sgouris JT. Studies on immune serum globulin (IgG) and its modification for intravenous administration. Prog Immunobiol Stand 1970;4:104-13.
422. Shane A, Hiebert J, Sherrard L, et al. Measles surveillance in Canada: Trends for 2013. Can Commun Dis Rep 2014;40(12):219-32. doi: 10.14745/ccdr.v40i12a01 [published Online First: 20140612]
423. Sharma R, Gaur A. Adverse events associated with Measles and Rubella vaccination campaign 2019 in India. Clin Exp Vaccine Res 2021;10(1):44-46. doi: 10.7774/cevr.2021.10.1.44 [published Online First: 20210131]
424. Sharma S, Agarwal S. Intralesional Immunotherapy with Measles Mumps Rubella Vaccine for the Treatment of Anogenital Warts: An Open-label Study. J Clin Aesthet Dermatol 2020;13(8):40-44. [published Online First: 20200801]
425. Shatrov, II, Mastiukova Iu N, Ignat'eva GV, et al. [Achievements in the control of measles mortality in the RSFSR during the years of Soviet power]. Zh Mikrobiol Epidemiol Immunobiol 1967;44(11):60-6.
426. Shears P, Lusty T. Communicable disease epidemiology following migration: studies from the African famine. Int Migr Rev 1987;21(3):783-95.
427. Sherrard L, Hiebert J, Squires S. Measles surveillance in Canada: Trends for 2014. Can Commun Dis Rep 2015;41(7):157-68. doi: 10.14745/ccdr.v41i07a01 [published Online First: 20150702]
428. Shimizu Y, Ohishi K, Suzuki R, et al. Amino acid sequence variations of signaling lymphocyte activation molecule and mortality caused by morbillivirus infection in cetaceans. Microbiol Immunol 2013;57(9):624-32. doi: 10.1111/1348-0421.12078
429. Shrivastava J, Narang M, Gomber S. Measles, mumps and rubella vaccine and heterologous immunity: a way out of the COVID-19 crisis? Sudan J Paediatr 2022;22(1):10-18. doi: 10.24911/sjp.106-1621869672
430. Shtykova EV, Petoukhov MV, Dadinova LA, et al. Solution Structure, Self-Assembly, and Membrane Interactions of the Matrix Protein from Newcastle Disease Virus at Neutral and Acidic pH. J Virol 2019;93(6) doi: 10.1128/jvi.01450-18 [published Online First: 20190305]
431. Siegrist CA, Barrios C, Martinez X, et al. Influence of maternal antibodies on vaccine responses: inhibition of antibody but not T cell responses allows successful early prime-boost strategies in mice. Eur J Immunol 1998;28(12):4138-48. doi: 10.1002/(sici)1521-4141(199812)28:12<4138::Aid-immu4138>3.0.Co;2-l
432. Siegrist CA, Córdova M, Brandt C, et al. Determinants of infant responses to vaccines in presence of maternal antibodies. Vaccine 1998;16(14-15):1409-14. doi: 10.1016/s0264-410x(98)00100-5
433. Sijtsma SR, West CE, Rombout JH, et al. The interaction between vitamin A status and Newcastle disease virus infection in chickens. J Nutr 1989;119(6):932-9. doi: 10.1093/jn/119.6.932
434. Simon B, Nomellini J, Chiou P, et al. Recombinant vaccines against infectious hematopoietic necrosis virus: production by the Caulobacter crescentus S-layer protein secretion system and evaluation in laboratory trials. Dis Aquat Organ 2001;44(1):17-27. doi: 10.3354/dao044017
435. Singh HM, Leber MF, Bossow S, et al. MicroRNA-sensitive oncolytic measles virus for chemovirotherapy of pancreatic cancer. Mol Ther Oncolytics 2021;21:340-55. doi: 10.1016/j.omto.2021.04.015 [published Online First: 20210505]
436. Singh S, Kishore D, Singh RK. Potential for Further Mismanagement of Fever During COVID-19 Pandemic: Possible Causes and Impacts. Front Med (Lausanne) 2022;9:751929. doi: 10.3389/fmed.2022.751929 [published Online First: 20220302]
437. Singh V. Tuberculosis in children: some issues. Health Millions 1995;21(1):27-8.
438. Sipahi S, Ozceker D, Gokcay G, et al. A Case of Anaphylaxis to Measles Vaccination in an Infant with Cow's Milk Allergy. Pediatr Allergy Immunol Pulmonol 2019;32(3):135-38. doi: 10.1089/ped.2019.0993 [published Online First: 20190917]
439. Slifka MK, Homann D, Tishon A, et al. Measles virus infection results in suppression of both innate and adaptive immune responses to secondary bacterial infection. J Clin Invest 2003;111(6):805-10. doi: 10.1172/jci13603

440. Socol Y, Shaki YY. Vaccinations: Mandatory or Voluntary? Risk-Benefit Analysis. Dose Response 2020;18(2):1559325820920116. doi: 10.1177/1559325820920116 [published Online First: 20200415]
441. Sojka PA, Ploog CL, Garner MM, et al. Acute human orthopneumovirus infection in a captive white-handed gibbon. J Vet Diagn Invest 2020;32(3):450-53. doi: 10.1177/1040638720910521 [published Online First: 20200313]
442. Soto S, Alba A, Ganges L, et al. Post-epizootic chronic dolphin morbillivirus infection in Mediterranean striped dolphins Stenella coeruleoalba. Dis Aquat Organ 2011;96(3):187-94. doi: 10.3354/dao02387
443. Station E, Guran M. The smoke that kills. Yanomami. Links 1992;9(2):11-2.
444. Statler VA, Fox T, Ardura MI. Spotting a potential threat: Measles among pediatric solid organ transplantation recipients. Pediatr Transplant 2023:e14502. doi: 10.1111/petr.14502 [published Online First: 20230315]
445. Stewart G, Chantry A, Lawson M. The Use of Oncolytic Viruses in the Treatment of Multiple Myeloma. Cancers (Basel) 2021;13(22) doi: 10.3390/cancers13225687 [published Online First: 20211113]
446. Sulistyawati S, Sumiana S. Measles Cluster Detection Using Ordinal Scan Statistic Model. Mater Sociomed 2018;30(4):282-86. doi: 10.5455/msm.2018.30.282-286
447. Sun J, Wei Y, Rauf A, et al. Methyltransferase-defective avian metapneumovirus vaccines provide complete protection against challenge with the homologous Colorado strain and the heterologous Minnesota strain. J Virol 2014;88(21):12348-63. doi: 10.1128/jvi.01095-14 [published Online First: 20140813]
448. Suvanbekov A, Kitarova G, Reyer JA, et al. Progress toward measles elimination in kyrgyzstan. Nagoya J Med Sci 2015;77(1-2):179-88.
449. Svitek N, von Messling V. Early cytokine mRNA expression profiles predict Morbillivirus disease outcome in ferrets. Virology 2007;362(2):404-10. doi: 10.1016/j.virol.2007.01.002 [published Online First: 20070207]
450. Swedish Council on Health Technology A. SBU Systematic Review Summaries. Vaccines to Children: Protective Effect and Adverse Events: A Systematic Review. Stockholm: Swedish Council on Health Technology Assessment (SBU)
Copyright © 2009 by the Swedish Council on Health Technology Assessment. 2009.
451. Sørup S, Stensballe LG, Krause TG, et al. Oral Polio Vaccination and Hospital Admissions With Non-Polio Infections in Denmark: Nationwide Retrospective Cohort Study. Open Forum Infect Dis 2016;3(1):ofv204. doi: 10.1093/ofid/ofv204 [published Online First: 20151217]
452. Tabak F, Murtezaoglu A, Tabak O, et al. Clinical features and etiology of adult patients with Fever and rash. Ann Dermatol 2012;24(4):420-5. doi: 10.5021/ad.2012.24.4.420 [published Online First: 20121108]
453. Tabutin D. [African population growth: status and prospects]. Tiers Monde 1991;32(125):159-73.
454. Takeda M, Ohno S, Seki F, et al. Long untranslated regions of the measles virus M and F genes control virus replication and cytopathogenicity. J Virol 2005;79(22):14346-54. doi: 10.1128/jvi.79.22.14346-14354.2005
455. Takemoto R, Suzuki T, Hashiguchi T, et al. Short-Stalk Isoforms of CADM1 and CADM2 Trigger Neuropathogenic Measles Virus-Mediated Membrane Fusion by Interacting with the Viral Hemagglutinin. J Virol 2022;96(3):e0194921. doi: 10.1128/jvi.01949-21 [published Online First: 20211117]
456. Tam L. What is child survival? Child-focused strategy forms key element of PLAN's health policy. Sante Salud 1993(1):3-4.
457. Tamura H, Kuraoka S, Hidaka Y, et al. A Case of Nephrotic Syndrome that Resolved with Influenza B Infection. Case Rep Nephrol Dial 2021;11(1):103-09. doi: 10.1159/000515062 [published Online First: 20210421]
458. Tandon BN. Health: the basic right of every child. ICCW News Bull 1989;37(3-4):9-12.
459. Tannous LK, Barlow G, Metcalfe NH. A short clinical review of vaccination against measles. JRSM Open 2014;5(4):2054270414523408. doi: 10.1177/2054270414523408 [published Online First: 20140312]
460. Tarabichi Y, Frees A, Honeywell S, et al. The Cosmos Collaborative: A Vendor-Facilitated Electronic Health Record Data Aggregation Platform. ACI open 2021;5(1):e36-e46. doi: 10.1055/s-0041-1731004
461. Tempfer C. [Vaccinations for pregnant women and women attempting to become pregnant]. Gynakol Endokrinol 2022;20(2):93-100. doi: 10.1007/s10304-022-00439-5 [published Online First: 20220201]
462. Terrero C, Demorizi JA, Feris JM, et al. [Incidence of measles with complications]. Arch Domin Pediatr 1992;28(2-3):31-4.
463. Theisen LL, Muller CP. EPs® 7630 (Umckaloabo®), an extract from Pelargonium sidoides roots, exerts anti-influenza virus activity in vitro and in vivo. Antiviral Res 2012;94(2):147-56. doi: 10.1016/j.antiviral.2012.03.006 [published Online First: 20120328]
464. Thompson S, Meyer JC, Burnett RJ, et al. Mitigating Vaccine Hesitancy and Building Trust to Prevent Future Measles Outbreaks in England. Vaccines (Basel) 2023;11(2) doi: 10.3390/vaccines11020288 [published Online First: 20230128]
465. Tom CM, Younesi S, Meer E, et al. Survival of iPSC-derived grafts within the striatum of immunodeficient mice: Importance of developmental stage of both transplant and host recipient. Exp Neurol 2017;297:118-28. doi: 10.1016/j.expneurol.2017.07.018 [published Online First: 20170729]
466. Tomás CC, Oliveira E, Sousa D, et al. Proceedings of the 3rd IPLeiria's International Health Congress : Leiria, Portugal. 6-7 May 2016. BMC Health Serv Res 2016;16 Suppl 3(Suppl 3):200. doi: 10.1186/s12913-016-1423-5 [published Online First: 20160706]
467. Tonelli D, Sacco Pde C, Calegari D, et al. Anesthesia in Guillain-Barré pediatric patient after measles vaccination. Case report. Rev Bras Anestesiol 2005;55(6):665-8. doi: 10.1590/s0034-70942005000600010
468. Torney HL, Bordt DE, Theodore H. Canine distemper immunization. Persistence of measles antibodies in dogs vaccinated with measles virus and the effect of passively-transferred measles antibodies on vaccination of puppies with measles virus. Vet Med Small Anim Clin 1967;62(11):1065-9.
469. Tsang JJ, Atkins HL. The ex vivo purge of cancer cells using oncolytic viruses: recent advances and clinical implications. Oncolytic Virother 2015;4:13-23. doi: 10.2147/ov.S45525 [published Online First: 20150123]

470. Tsegaye G, Gezahagn Y, Bedada S, et al. Epidemiology of Measles in Bale Zone, Southeast Ethiopia: Analysis of Surveillance Data from 2013 to 2019. Risk Manag Healthc Policy 2021;14:4093-103. doi: 10.2147/rmhp.S325173 [published Online First: 20211001]
471. Tugbo LP. NID: ceasefire for the future. NCP Bull 1993:4-5.
472. Turaiche M, Feciche B, Gluhovschi A, et al. Biological Profile and Clinical Features as Determinants for Prolonged Hospitalization in Adult Patients with Measles: A Monocentric Study in Western Romania. Pathogens 2022;11(9) doi: 10.3390/pathogens11091018 [published Online First: 20220907]
473. Ulak M, Chandyo RK, Mellander L, et al. Infant feeding practices in Bhaktapur, Nepal: a cross-sectional, health facility based survey. Int Breastfeed J 2012;7(1):1. doi: 10.1186/1746-4358-7-1 [published Online First: 20120110]
474. Ulfat LR. Universal immunization, a chance for every child. Yojana 1987;31(10):26-8.
475. Unasova TN, Binyatova AS, Phadeykina OV, et al. ANALYSIS OF THE QUALITY OF NATIONAL VACCINE AGAINST RUBELLA. Vopr Virusol 2018;63(2):90-96. doi: 10.18821/0507-4088-2018-63-2-90-96 [published Online First: 20180420]
476. Ungerechts G, Springfeld C, Frenzke ME, et al. An immunocompetent murine model for oncolysis with an armed and targeted measles virus. Mol Ther 2007;15(11):1991-7. doi: 10.1038/sj.mt.6300291 [published Online First: 20070821]
477. Veinalde R, Pidelaserra-Martí G, Moulin C, et al. Oncolytic measles vaccines encoding PD-1 and PD-L1 checkpoint blocking antibodies to increase tumor-specific T cell memory. Mol Ther Oncolytics 2022;24:43-58. doi: 10.1016/j.omto.2021.11.020 [published Online First: 20211129]
478. Veinalde R, Pidelaserra-Martí G, Moulin C, et al. Virotherapy combined with anti-PD-1 transiently reshapes the tumor immune environment and induces anti-tumor immunity in a preclinical PDAC model. Front Immunol 2022;13:1096162. doi: 10.3389/fimmu.2022.1096162 [published Online First: 20230116]
479. Verrier F, de Lauzanne A, Diouf JN, et al. Vaccination Coverage and Risk Factors Associated With Incomplete Vaccination Among Children in Cambodia, Madagascar, and Senegal. Open Forum Infect Dis 2023;10(4):ofad136. doi: 10.1093/ofid/ofad136 [published Online First: 20230310]
480. Wang L, Zhu L, Zhu H. Efficacy of varicella (VZV) vaccination: an update for the clinician. Ther Adv Vaccines 2016;4(1-2):20-31. doi: 10.1177/2051013616655980 [published Online First: 20160101]
481. Wang R, Jing W, Liu M, et al. Trends of the Global, Regional, and National Incidence of Measles, Vaccine Coverage, and Risk Factors in 204 Countries From 1990 to 2019. Front Med (Lausanne) 2021;8:798031. doi: 10.3389/fmed.2021.798031 [published Online First: 20220120]
482. Watari E, Shinya E, Kurane S, et al. Effects of cyclosporin A on cell fusion in a monkey kidney cell line persistently infected with measles virus. Intervirology 2001;44(4):209-14. doi: 10.1159/000050048
483. Weger-Lucarelli J, Carrau L, Levi LI, et al. Host nutritional status affects alphavirus virulence, transmission, and evolution. PLoS Pathog 2019;15(11):e1008089. doi: 10.1371/journal.ppat.1008089 [published Online First: 20191111]
484. Weidinger G, Czub S, Neumeister C, et al. Role of CD4(+) and CD8(+) T cells in the prevention of measles virus-induced encephalitis in mice. J Gen Virol 2000;81(Pt 11):2707-13. doi: 10.1099/0022-1317-81-11-2707
485. Welaga P, Hodgson A, Debpuur C, et al. Measles Vaccination Supports Millennium Development Goal 4: Increasing Coverage and Increasing Child Survival in Northern Ghana, 1996-2012. Front Public Health 2018;6:28. doi: 10.3389/fpubh.2018.00028 [published Online First: 20180212]
486. Westerfeld N, Pluschke G, Zurbriggen R. Optimized Malaria-antigens delivered by immunostimulating reconstituted influenza virosomes. Wien Klin Wochenschr 2006;118(19-20 Suppl 3):50-7. doi: 10.1007/s00508-006-0684-0
487. White LK, Yoon JJ, Lee JK, et al. Nonnucleoside inhibitor of measles virus RNA-dependent RNA polymerase complex activity. Antimicrob Agents Chemother 2007;51(7):2293-303. doi: 10.1128/aac.00289-07 [published Online First: 20070430]
488. Williams-thomas G. Disease and inequalities in infant and child mortality in rural Kenya. Ann IFORD 1988;12(2):23-33.
489. Wilson JH, Hermann-Dekkers WM, Leemans-Dessy S, et al. Experiements with an inactivated hepatitis leptospirosis vaccine in vaccination programmes for dogs. Vet Rec 1977;100(26):552-4. doi: 10.1136/vr.100.26.552
490. Wohlsein P, Müller G, Haas L, et al. Antigenic characterization of phocine distemper virus causing mass mortality in 2002 and its relationship to other morbilliviruses. Arch Virol 2007;152(8):1559-64. doi: 10.1007/s00705-007-0970-9 [published Online First: 20070426]
491. Wolf RF, Eberle R, White GL. Generation of a specific-pathogen-free baboon colony. J Am Assoc Lab Anim Sci 2010;49(6):814-20.
492. Wooley RE, Gilbert JP, Whitehead WK, et al. Survival of viruses in fermented edible waste material. Am J Vet Res 1981;42(1):87-90.
493. Wu XM, Liao YW, Wang HY, et al. Integrin alphavbeta6 is involved in measles protein-induced airway immune suppression. Cytokine 2012;59(1):59-64. doi: 10.1016/j.cyto.2012.04.005 [published Online First: 20120509]
494. Wutzler P, Knuf M, Liese J. Varicella: efficacy of two-dose vaccination in childhood. Dtsch Arztebl Int 2008;105(33):567-72. doi: 10.3238/arztebl.2008.0567 [published Online First: 20080815]
495. Xu L, Li N, Zhang L, et al. Febrile Seizures and Measles-Containing Vaccines in China: A Self-Controlled Case Series Study. Vaccines (Basel) 2021;9(10) doi: 10.3390/vaccines9101073 [published Online First: 20210924]
496. Yang W. Transmission dynamics of and insights from the 2018-2019 measles outbreak in New York City: A modeling study. Sci Adv 2020;6(22):eaaz4037. doi: 10.1126/sciadv.aaz4037 [published Online First: 20200527]
497. Yee JL, McChesney MB, Christe KL. Multicenter Safety and Immunogenicity Trial of an Attenuated Measles Vaccine for NHP. Comp Med 2015;65(5):448-54.

498. Yoneda M, Georges-Courbot MC, Ikeda F, et al. Recombinant measles virus vaccine expressing the Nipah virus glycoprotein protects against lethal Nipah virus challenge. PLoS One 2013;8(3):e58414. doi: 10.1371/journal.pone.0058414 [published Online First: 20130314]
499. Zhang CD, Wang YL, Zhou DM, et al. A recombinant Chinese measles virus vaccine strain rMV-Hu191 inhibits human colorectal cancer growth through inducing autophagy and apoptosis regulating by PI3K/AKT pathway. Transl Oncol 2021;14(7):101091. doi: 10.1016/j.tranon.2021.101091 [published Online First: 20210410]
500. Zhang Y, Gabere M, Taylor MA, et al. Repurposing live attenuated trivalent MMR vaccine as cost-effective cancer immunotherapy. Front Oncol 2022;12:1042250. doi: 10.3389/fonc.2022.1042250 [published Online First: 20221109]
501. Zhao F, Guo J, Chen H. Studies on the relationship between changes in dietary patterns and health status. Asia Pac J Clin Nutr 1995;4(3):294-7.
502. Zhao J, Ren Y. Multiple Receptors Involved in Invasion and Neuropathogenicity of Canine Distemper Virus: A Review. Viruses 2022;14(7) doi: 10.3390/v14071520 [published Online First: 20220712]
503. Zhao S, Tang X, Liang X, et al. Modelling the Measles Outbreak at Hong Kong International Airport in 2019: A Data-Driven Analysis on the Effects of Timely Reporting and Public Awareness. Infect Drug Resist 2020;13:1851-61. doi: 10.2147/idr.S258035 [published Online First: 20200617]
504. Zhu YD, Fennelly G, Miller C, et al. Recombinant bacille Calmette-Guérin expressing the measles virus nucleoprotein protects infant rhesus macaques from measles virus pneumonia. J Infect Dis 1997;176(6):1445-53. doi: 10.1086/514140
505. Zi W. [Monetary incentives for a more effective immunization program]. Foro Mund Salud 1990;11(2):173-8.
506. Zibolenová J, Hudečková H, Chladná Z, et al. Quantification of waning immunity after measles vaccination - evidence from a seroprevalence study. Am J Epidemiol 2023 doi: 10.1093/aje/kwad065 [published Online First: 20230317]
507. Zlotnik I, Grant DP. Further observations on subacute sclerosing encephalitis in adult hamsters: the effects of intranasal infections with Langat virus, measles virus and SSPE-measles virus. Br J Exp Pathol 1976;57(1):49-66.
508. Aaby P, Benn C, Nielsen J, et al. Testing the hypothesis that diphtheria-tetanus-pertussis vaccine has negative non-specific and sex-differential effects on child survival in high-mortality countries. BMJ Open 2012;2(3) doi: 10.1136/bmjopen-2011-000707 [published Online First: 20120522]
509. Aaby P, Martins CL, Garly ML, et al. The optimal age of measles immunisation in low-income countries: a secondary analysis of the assumptions underlying the current policy. BMJ Open 2012;2(4) doi: 10.1136/bmjopen-2011-000761 [published Online First: 20120719]
510. Aaby P, Mogensen SW, Rodrigues A, et al. Evidence of Increase in Mortality After the Introduction of Diphtheria-Tetanus-Pertussis Vaccine to Children Aged 6-35 Months in Guinea-Bissau: A Time for Reflection? Front Public Health 2018;6:79. doi: 10.3389/fpubh.2018.00079 [published Online First: 20180319]
511. Aaby P, Nielsen S, Fisker AB, et al. Stopping Oral Polio Vaccine (OPV) After Defeating Poliomyelitis in Low- and Middle-Income Countries: Harmful Unintended Consequences? Review of the Nonspecific Effects of OPV. Open Forum Infect Dis 2022;9(8):ofac340. doi: 10.1093/ofid/ofac340 [published Online First: 20220727]
512. Aaron TS, Fooksman DR. Dynamic organization of the bone marrow plasma cell niche. Febs j 2022;289(14):4228-39. doi: 10.1111/febs.16385 [published Online First: 20220212]

Citations excluded due to language barrier

1. Prato R, Martinelli D, Calabria S, et al. [Inclusion of vaccination into clinical pathways for COPD and asthma: current challenges and future perspectives in Italy]. Epidemiol Prev 2023;47(1-2):In press. doi: 10.19191/ep23.1-2.A521.015
2. Herrero-Diez MT, Catalá-López F. [Vaccination coverage, beliefs, and attitudes in transplanted children and adolescents: a mixed-methods systematic review.]. Rev Esp Salud Publica 2023;97 [published Online First: 20230330]
3. Tempfer C. [Vaccinations for pregnant women and women attempting to become pregnant]. Gynakol Endokrinol 2022;20(2):93-100. doi: 10.1007/s10304-022-00439-5 [published Online First: 20220201]
4. Jablonowski L, Gardemann J, Smollich M, et al. [Opinion Survey of Paediatricians on Vaccination Status of Refugee Children - Challenges of a Medical Connection to the Regular Outpatient Care System]. Gesundheitswesen 2022;84(7):575-80. doi: 10.1055/a-1585-1679 [published Online First: 20211102]
5. Chekhlyaeva TS, Erokhov DV, Andrievskaya IY, et al. [Genetic diversity of the mumps viruses (Paramyxoviridae: Orthorubulavirus: Mumps orthorubulavirus): an overview]. Vopr Virusol 2022;67(2):95-106. doi: 10.36233/0507-4088-98 [published Online First: 20220505]
6. Xia M, Meng G, Dong J. [Synergistic Effect of NF-κB Signaling Pathway Inhibitor and Oncolytic Measles Virus Vaccine Strain against Lung Cancer and Underlying Mechanisms]. Zhongguo Fei Ai Za Zhi 2021;24(4):245-53. doi: 10.3779/j.issn.1009-3419.2021.102.14 [published Online First: 20210329]
7. Mernyk A, Yaroshenko O, Inshyn M, et al. [VACCINATION: HUMAN RIGHT OR DUTY]. Georgian Med News 2021(315):135-40.
8. Limia Sánchez A, Olmedo Lucerón C. [2(nd) Seroprevalence Study in Spain, 2017-2018]. Rev Esp Salud Publica 2021;95 [published Online First: 20210318]
9. Rincón-León HA, Navarro-Fuentes KR. [Measles: a millennial itch in the COVID-19 era]. Rev Med Inst Mex Seguro Soc 2020;58(6):644-47. doi: 10.24875/rmimss.M20000095

10. Pfäfflin F, Flick H, Vogtmann M. [Rare viral infections of the lungs]. Pneumologe (Berl) 2020;17(5):311-21. doi: 10.1007/s10405-020-00337-2 [published Online First: 20200720]
11. Jorquera LA, Ugarte CD, Avilés LC, et al. [Aseptic meningitis due to mumps vaccine. Case report and review of the literature]. Rev Chilena Infectol 2020;37(6):769-74. doi: 10.4067/s0716-10182020000600769
12. Billon-Denis E, Tournier JN. [COVID-19 and vaccination: a global disruption]. Med Sci (Paris) 2020;36(11):1034-37. doi: 10.1051/medsci/2020203 [published Online First: 20201105]
13. Yang WZ. [Dramatic achievements in infectious disease prevention and treatment in China during the past 70 years]. Zhonghua Liu Xing Bing Xue Za Zhi 2019;40(12):1493-98. doi: 10.3760/cma.j.issn.0254-6450.2019.12.001
14. Xiao JX, Lin ZQ, Wu RH, et al. [Analysis on the characteristics of suspected vaccine-related deaths in Fujian Province, 2012-2017]. Zhonghua Yu Fang Yi Xue Za Zhi 2019;53(7):719-23. doi: 10.3760/cma.j.issn.0253-9624.2019.07.012
15. Fadic RR, Repetto DG. [Measles: Historical background and current situation]. Rev Chil Pediatr 2019;90(3):253-59. doi: 10.32641/rchped.v90i3.1231
16. Domínguez A, Astray J, Castilla J, et al. [False beliefs about vaccines]. Aten Primaria 2019;51(1):40-46. doi: 10.1016/j.aprim.2018.05.004 [published Online First: 20180924]
17. Ali D, Gorur Y, Bosquée L, et al. [Hypoxemic measles pneumonitis in an immunocompetent adult]. Rev Med Liege 2019;74(10):499-502.
18. Yan R, He B, Yao FY, et al. [Investigation of a measles outbreak caused by genotype D8 virus in Pinghu city of Zhejiang province, 2017]. Zhonghua Liu Xing Bing Xue Za Zhi 2018;39(3):333-36. doi: 10.3760/cma.j.issn.0254-6450.2018.03.016
19. Tulen AD, van Wijhe M, Korthals Altes H, et al. [Quantifying the impact of mass vaccination programmes on notified cases in the Netherlands]. Ned Tijdschr Geneeskd 2018;162 [published Online First: 20180920]
20. Karathana M, Krackhardt B, Schade M, et al. [School Entrance Examination for Lateral Entrants - What Can and What Should They be Able to Do? A Discussion Contribution Based on the Data from the Health Authority Frankfurt am Main 2006-2016]. Gesundheitswesen 2018;80(4):317-24. doi: 10.1055/s-0043-121887 [published Online First: 20171215]
21. Devecioğlu E, Gökçay G, Boran P, et al. [Prevalence of maternal measles, rubella, mumps and varicella antibodies in the first six months of life]. Mikrobiyol Bul 2018;52(3):324-27. doi: 10.5578/mb.67169
22. Alikova ZR, Anaeva LA, Fidarova KK, et al. [The Priority Directions of Children Health Support in Conditions of the Region During the Great Patriotic War]. Probl Sotsialnoi Gig Zdravookhranenniiai Istor Med 2018;26(2):124-28. doi: 10.1016/0869-866x-2018-26-2-124-128
23. Villani A, Bozzola E, Vittucci AC, et al. [Vaccination in childhood: how to protect the "unprotectable"?]. Ig Sanita Pubbl 2017;73(5):443-52.
24. Kristian Thorlund JE, Jørn Wetterslev, Jesper Brok, Georgina Imberger, and Christian Gluud User Manual for Trial Sequential Analysis (TSA). 2017.
25. Hebert A, Louis O, Hankenne L, et al. [Epidemic of measles in the Verviers area (Belgium) : management and precautions at the reception in the emergency department]. Rev Med Liege 2017;72(9):406-09.
26. De Waure C, Sisti LG, Poscia A, et al. [The new National Immunization Program 2017-2019 and the Essential Care Levels: what is going to change?]. Ig Sanita Pubbl 2017;73(5):405-18.
27. Baudon JJ. [The advent of a newborn specialty: 19th century pediatrics]. Presse Med 2017;46(4):438-48. doi: 10.1016/j.lpm.2017.01.021 [published Online First: 20170406]
28. Adamo G, Sturabotti G, De Soccio P, et al. [The elimination of measles and rubella in Italy]. Ig Sanita Pubbl 2017;73(5):429-42.
29. Yeşilbaş O, Şevketoğlu E, Kıhtır HS, et al. [A case of bronchiolitis obliterans secondary to human metapneumovirus bronchiolitis]. Mikrobiyol Bul 2016;50(4):606-12. doi: 10.5578/mb.32195
30. Niederer-Loher A. [Vaccinations in pregnancy – Don't miss the opportunity!]. Ther Umsch 2016;73(5):269-73. doi: 10.1024/0040-5930/a000791
31. Martinez L, Tugaut B, Raineri F, et al. [The commitment of French general practitioners to vaccination: the DIVA study (Determinants of Vaccination Intentions)]. Sante Publique 2016;28(1):19-32.
32. Lu L, Cao YM, Yang QY, et al. [Dynamic maternal measles antibody level in infants: a longitudinal study]. Zhonghua Liu Xing Bing Xue Za Zhi 2016;37(5):663-7. doi: 10.3760/cma.j.issn.0254-6450.2016.05.015
33. Lin H, Yang J, Liu TX. [Changing trend of four immune-related diseases in Ningxia]. Zhonghua Liu Xing Bing Xue Za Zhi 2016;37(8):1127-30. doi: 10.3760/cma.j.issn.0254-6450.2016.08.015
34. Kühne A, Gilsdorf A. [Infectious disease outbreaks in centralized homes for asylum seekers in Germany from 2004-2014]. Bundesgesundheitsblatt Gesundheitsforschung Gesundheitsschutz 2016;59(5):570-7. doi: 10.1007/s00103-016-2332-9
35. Karakeçili F, Akın H, Çıkman A, et al. [Measles outbreak in the adult age group: evaluation of 28 cases]. Mikrobiyol Bul 2016;50(1):112-21. doi: 10.5578/mb.10692
36. Aleshkin VA, Tikhonova NT, Gerasimova AG, et al. [PROBLEMS TOWARDS MEASLES ELIMINATION IN RUSSIAN FEDERATION]. Zh Mikrobiol Epidemiol Immunobiol 2016(5):29-34.
37. Wang L, Zeng L, Ren X, et al. [Analysis of morbidity and mortality characteristics of the notifiable diseases reported in 2013 in China]. Zhonghua Liu Xing Bing Xue Za Zhi 2015;36(3):194-8.
38. Shmit'ko AD, Kostinov MP, Bocharova, II, et al. [Transplacental IgG against measles virus in umbilical blood of neonates from various regions of Russian Federation]. Zh Mikrobiol Epidemiol Immunobiol 2015(1):52-7.
39. Pei L, Wen GF, Song WL, et al. [Risk factors for the development of acute respiratory distress syndrome in children with measles]. Zhongguo Dang Dai Er Ke Za Zhi 2015;17(3):245-8.
40. Ma R, Lu L, Zhang Z, et al. [Changes of epidemiological characteristics of measles in Beijing before and after supplementary immunization campaigns of measles vaccine in 2010]. Zhonghua Yu Fang Yi Xue Za Zhi 2015;49(12):1036-41.

41. Han S, Ye Y, Cao L, et al. [Analysis on clinical and epidemioloical characteristics of measles in hospitalized children in Shanghai in 2012]. Zhonghua Er Ke Za Zhi 2015;53(8):605-9.
42. Delpiano L, Astroza L, Toro J. [Measles: the disease, epidemiology, history and vaccination programs in Chile]. Rev Chilena Infectol 2015;32(4):417-29. doi: 10.4067/s0716-10182015000500008
43. Caseris M, Burdet C, Lepeule R, et al. [An update on measles]. Rev Med Interne 2015;36(5):339-45. doi: 10.1016/j.revmed.2014.10.362 [published Online First: 20150108]
44. Boushab BM, Savadogo M, Sow MS, et al. [Epidemiological, clinical, and prognostic study of the measles in the Aioun regional hospital in Mauritania]. Med Sante Trop 2015;25(2):180-3. doi: 10.1684/mst.2015.0447
45. Begaydarova R, Starikov YG, Devdariani Kh G, et al. [The current knowledge of clinical manifestations of measles]. Georgian Med News 2015(239):63-9.
46. Ramírez-Tirado LA, Tirado-Gómez LL, López-Cervantes M. [Inequality in primary care interventions in maternal and child health care in Mexico]. Rev Panam Salud Publica 2014;35(4):235-41.
47. Onishchenko GG, Ezhlova EB, Mel'nikova AA. [Actual problems of vaccine prophylaxis in the Russian Federation]. Zh Mikrobiol Epidemiol Immunobiol 2014(1):9-19.
48. Metin O, Tanir G, Oz FN, et al. [Evaluation of 44 pediatric measles cases detected in Ankara, Turkey during 2012-2013 epidemic and molecular characterization of the viruses obtained from two cases]. Mikrobiyol Bul 2014;48(2):259-70. doi: 10.5578/mb.7024
49. Martínez-Ramírez M, González-Praetorius A, Ory-Manchón F, et al. [Re-emergence of measles in the province of Guadalajara, Spain. Is it time to establish new strategies for its elimination?]. Enferm Infecc Microbiol Clin 2014;32(8):486-90. doi: 10.1016/j.eimc.2013.08.005 [published Online First: 20131107]
50. Kim DJ, Yoo HS, Lee H. [Effects of the periodical spread of rinderpest on famine, epidemic, and tiger disasters in the late 17th Century]. Uisahak 2014;23(1):1-56. doi: 10.13081/kjmh.2014.23.1
51. Gunnarsdottir S, Briem H, Gottfredsson M. [Extent and impact of the measles epidemics of 1846 and 1882 in Iceland]. Laeknabladid 2014;100(4):211-6. doi: 10.17992/lbl.2014.04.539
52. Garzuly F, Schneider F, Iványi JL, et al. [Changing times - changing diseases. Review of the neuropathological autopsy documentations at the Markusovszky University Teaching Hospital (1964-2014)]. Orv Hetil 2014;155(43):1722-8. doi: 10.1556/oh.2014.29996
53. Donas A, Marty-Nussbaumer A, Roost HP, et al. [Measles epidemic in a highly developed country: low mortality, high morbidity and extensive costs]. Klin Padiatr 2014;226(1):13-8. doi: 10.1055/s-0033-1363254 [published Online First: 20140116]
54. Wu XY, Luo ZX, Fu Z, et al. [Clinical analysis of 28 cases of bronchiolitis obliterans]. Zhongguo Dang Dai Er Ke Za Zhi 2013;15(10):845-9.
55. Wichmann O, Ultsch B. [Effectiveness, population-level effects, and heath economics of measles and rubella vaccination]. Bundesgesundheitsblatt Gesundheitsforschung Gesundheitsschutz 2013;56(9):1260-9. doi: 10.1007/s00103-013-1801-7
56. Sun XD, Li CS, Tang X, et al. [Genetic characterization analysis on the first imported measles virus of genotype D8 in Chinese mainland]. Bing Du Xue Bao 2013;29(6):583-8.
57. Siedler A, Hecht J, Rieck T, et al. [Varicella vaccination in Germany. A provisional appraisal in the context of MMR vaccination]. Bundesgesundheitsblatt Gesundheitsforschung Gesundheitsschutz 2013;56(9):1313-20. doi: 10.1007/s00103-013-1789-z
58. Sáenz-González MC, Hernández-García I. [Immunization practices for workers. Update recommendations]. Rev Esp Quimioter 2013;26(4):287-97.
59. Moreno-Pérez D, Álvarez García FJ, Arístegui Fernández J, et al. [Immunisation schedule of the Spanish Association of Paediatrics: 2013 recommendations]. An Pediatr (Barc) 2013;78(1):59.e1-27. doi: 10.1016/j.anpedi.2012.10.002 [published Online First: 20121124]
60. Fukuhara H, Chen S, Takeda S, et al. [Entry mechanism of morbillivirus family]. Yakugaku Zasshi 2013;133(5):549-59. doi: 10.1248/yakushi.13-00001-5
61. Braun-Fahrländer C. [The role of the microbial environment for the development of childhood asthma and allergies]. Ther Umsch 2013;70(12):714-9. doi: 10.1024/0040-5930/a000469
62. Battistella M, Carlino C, Dugo V, et al. [Vaccines and autism: a myth to debunk?]. Ig Sanita Pubbl 2013;69(5):585-96.
63. Tuppin P, Blotière PO. [Hospitalization rates for immigrant-related illness among individuals with low income and full health insurance coverage in France, 2009]. Bull Soc Pathol Exot 2012;105(2):79-85. doi: 10.1007/s13149-012-0213-5
64. Quiroga EF. [Death rate by malnutrition in children under the age of five, Colombia]. Biomedica 2012;32(4):499-509. doi: 10.1590/s0120-41572012000400005
65. [Monitoring of implementation of international programs of poliomyelitis eradication and measles and rubella elimination in the Republic of Belarus]. Zh Mikrobiol Epidemiol Immunobiol 2012(1):21-30.
66. Tiembré I, Benié J, Coulibaly A, et al. [Impact of armed conflict on the health care system of a sanitary district in Cote d'Ivoire]. Med Trop (Mars) 2011;71(3):249-52.
67. Scrivener Y, Marcil T, Lipsker D, et al. [Measles: a follicular disease]. Ann Dermatol Venereol 2011;138(2):111-5. doi: 10.1016/j.annder.2011.01.002 [published Online First: 20110203]
68. Onishchenko GG. [Topical problems of the modern epidemiology (data for presentation at the all-Russian scientific conference "The problems of modern epidemiology. Perspective means and methods for the laboratory diagnosis and prevention of urgent infections")]. Gig Sanit 2011(4):4-13.
69. Omba Kalonda JC. [Socioeconomic impact of armed conflict on the health of women and children in the Democratic Republic of the Congo]. Med Trop (Mars) 2011;71(2):192-6.

70. Nishiwaki T, Sawa N, Kikui S. [Suspected measles encephalitis caused by modified measles that was difficult to diagnose: a case report]. Brain Nerve 2011;63(1):75-8.
71. Monsel G, Rapp C, Duong TA, et al. [Measles in adults: an emerging disease not sparing medical staff]. Ann Dermatol Venereol 2011;138(2):107-10. doi: 10.1016/j.annder.2010.12.015 [published Online First: 20110201]
72. Martins S, Rocha G, Silva G, et al. [Blueberry muffin baby. A rare presentation of congenital cytomegalovirus infection]. Acta Med Port 2011;24 Suppl 3:703-8. [published Online First: 20111231]
73. Biron C, Beaudoux O, Ponge A, et al. [Measles in the Nantes Teaching Hospital during the 2008-2009 epidemic]. Med Mal Infect 2011;41(8):415-23. doi: 10.1016/j.medmal.2010.09.002 [published Online First: 20110623]
74. Belov AB, Ogarkov PI. [Current issues of epidemiology and vaccine prophylaxis of "children" airborne infections in the armies (Navy)]. Voen Med Zh 2011;332(4):28-33.
75. Anselem O, Tsatsaris V, Lopez E, et al. [Measles and pregnancy]. Presse Med 2011;40(11):1001-7. doi: 10.1016/j.lpm.2011.07.002 [published Online First: 20110831]
76. Zieliński A, Czarkowski MP. [Infectious diseases in Poland in 2008]. Przegl Epidemiol 2010;64(2):151-8.
77. Viesca-Treviño C. [Epidemics and diseases during the Independence period in Mexico]. Rev Med Inst Mex Seguro Soc 2010;48(1):47-54.
78. Monfort L, Muñoz D, Trenchs V, et al. [Measles outbreak in Barcelona. Clinical and epidemiological characteristics]. Enferm Infecc Microbiol Clin 2010;28(2):82-6. doi: 10.1016/j.eimc.2009.05.006 [published Online First: 20090806]
79. Lang PO, Michel JP. [Vaccination: a programme for the whole life]. Rev Med Suisse 2010;6(270):2118-24.
80. Ion-Nedelcu N, Iordăchescu C, Mihailovici R, et al. [Effect of systematic vaccination on the epidemiology of urlian parotiditis in the population of Bucharest]. Bacteriol Virusol Parazitol Epidemiol 2010;55(1):35-9.
81. Campagna M, Bacis M, Belotti L, et al. [Exanthemic diseases (measles, chickenpox, rubella and parotitis). Focus on screening and health surveillance of health workers: results and perspectives of a multicenter working group]. G Ital Med Lav Ergon 2010;32(3):298-303.
82. Bégué P. [Consequences of opposition to vaccination in France and Europe. How to maintain effective vaccine coverage in 2010?]. Bull Acad Natl Med 2010;194(4-5):719-32; discussion 32.
83. Tomita T, Saeki Y, Shigita E, et al. [Immunization of pharmacy students in Japan]. Yakugaku Zasshi 2009;129(4):451-7. doi: 10.1248/yakushi.129.451
84. Stock I. [Measles]. Med Monatsschr Pharm 2009;32(4):118-26; quiz 27-8.
85. Shchavkhalov RN. [The prevention of infectious diseases in the framework of the national project "Health"]. Probl Sotsialnoi Gig Zdravookhranenniiai Istor Med 2009(6):14-7.
86. Pinquier D, Adde-Michela C, Ploin D, et al. [Vaccination rate of premature infants at 6 and 24 months of age: a pilot study]. Arch Pediatr 2009;16(12):1533-9. doi: 10.1016/j.arcped.2009.09.009 [published Online First: 20091024]
87. Mijiti MT, Sun H, Zhang Y. [Analysis on clinical characteristics of adult measles inpatients in Urumqi Municipal]. Zhongguo Yi Miao He Mian Yi 2009;15(6):516-7.
88. Markushin SG, Litvin AA. [Antiviral activity of panavir in experimental Measles virus infection in cell cultures]. Antibiot Khimioter 2009;54(1-2):14-6.
89. Ekra KD, Attoh-Touré H, Bénié BV, et al. [Five years of cholera surveillance in Ivory Coast during social and political crisis, 2001 to 2005]. Bull Soc Pathol Exot 2009;102(2):107-9.
90. Cao GQ, Yang Y, Zhang ZG. [Effect of measles control at different stages in Binzhou Municipal]. Zhongguo Yi Miao He Mian Yi 2009;15(5):426-8, 72.
91. Bo F, Cui AL, Guo XB. [Analysis epidemiological and pathogen characters of mumps in China from 2004 to 2006]. Zhongguo Yi Miao He Mian Yi 2009;15(2):115-8.
92. Yé D, Kouéta F, Dao L, et al. [Pediatric management of sickle cell disease: experience at the Charles de Gaulle University Children's Hospital in Ouagadougou (Burkina Faso)]. Sante 2008;18(2):71-5. doi: 10.1684/san.2008.0113
93. Xu GZ, Ma R, Xu HJ, et al. [Levels of transition on maternal transferred measles antibody in infants in 3 cities in China]. Zhonghua Liu Xing Bing Xue Za Zhi 2008;29(11):1074-7.
94. Valleron AJ. [Mortality and morbidity worldwide, now and tomorrow: what is known?]. C R Biol 2008;331(12):991-1006. doi: 10.1016/j.crvi.2008.09.002 [published Online First: 20081001]
95. Stefanoff P, Rogalska J. [Rubella in Poland in 2006]. Przegl Epidemiol 2008;62(2):229-34.
96. Onishchenko GG. [The surveillance of nonspecific prevention of infectious diseases and the tasks of its improvement]. Gig Sanit 2008(1):4-10.
97. Gabutti G, Azzari C. [Priorix Tetra: a new combined vaccine against measles, rubella, mumps and varicella]. Minerva Pediatr 2008;60(4):429-41.
98. Dias-da-Costa JS, Borba LG, Pinho MN, et al. [Quality of primary care as measured by preventable hospitalizations in the South of Brazil]. Cad Saude Publica 2008;24(7):1699-707. doi: 10.1590/s0102-311x2008000700024
99. Berner W. [History of the control of acute infectious diseases in Poland after the World War I--until the year 1924 (including big cities)]. Przegl Epidemiol 2008;62(4):849-59.
100. Tsutsumi H. [Transplacentally acquired immunoglobulin G antibodies in newborns and infants]. Nihon Rinsho 2007;65 Suppl 2 Pt. 1:627-31.
101. Stefanoff P, Czarkowski MP, Kondej B. [Measles in Poland in 2005]. Przegl Epidemiol 2007;61(2):189-93.
102. Sepúlveda J, Bustreo F, Tapia R, et al. [Improvement of child survival in Mexico: the diagonal approach]. Salud Publica Mex 2007;49 Suppl 1:S110-25.
103. Seguljev Z, Petrović V, Cosić G, et al. [Effects of the immunization program in Vojvodina]. Med Pregl 2007;60(11-12):553-7. doi: 10.2298/mpns0712553s

104. Saliou P. [Vaccination and development in sub-Saharian Africa]. Bull Acad Natl Med 2007;191(8):1589-99; discussion 99.
105. Saliou P. [Eradication of infectious diseases by vaccination]. Med Trop (Mars) 2007;67(4):321-7.
106. Porru S, Campagna M, Arici C, et al. [Susceptibility to varicella-zoster, measles, rosacea and mumps among health care workers in a Northern Italy hospital]. G Ital Med Lav Ergon 2007;29(3 Suppl):407-9.
107. Oliveira JM, Rondó PH. [Evidence of the impact of vitamin A supplementation on maternal and child health]. Cad Saude Publica 2007;23(11):2565-75. doi: 10.1590/s0102-311x2007001100004
108. Nereida V, Gotera J. [Towards the elimination of measles in Venezuela]. Invest Clin 2007;48(2):135-7.
109. Makówka A, Gut W, Litwińska B. [Measles elimination programme on the world and Poland]. Przegl Epidemiol 2007;61(1):135-42.
110. Leclerc C. [New technologies for vaccine development]. Med Sci (Paris) 2007;23(4):386-90. doi: 10.1051/medsci/2007234386
111. Kriz B, Fabiánová K, Maixnerová M, et al. [Pertussis: a reemerging infection?]. Epidemiol Mikrobiol Imunol 2007;56(2):51-65.
112. Kamtsiuris P, Atzpodien K, Ellert U, et al. [Prevalence of somatic diseases in German children and adolescents. Results of the German Health Interview and Examination Survey for Children and Adolescents (KiGGS)]. Bundesgesundheitsblatt Gesundheitsforschung Gesundheitsschutz 2007;50(5-6):686-700. doi: 10.1007/s00103-007-0230-x
113. Ion-Nedelcu N, Iordachescu C, Moculescu C, et al. [Nature and severity of the complications in the measles cases hospitalized during the epidemics in 2005-2006 in Bucharest]. Bacteriol Virusol Parazitol Epidemiol 2007;52(3-4):149-56.
114. Enders M, Biber M, Exler S. [Measles, mumps and rubella virus infection in pregnancy. Possible adverse effects on pregnant women, pregnancy outcome and the fetus]. Bundesgesundheitsblatt Gesundheitsforschung Gesundheitsschutz 2007;50(11):1393-8. doi: 10.1007/s00103-007-0195-9
115. Djadou KE, Douti K, Koffi S, et al. [Morbidity in a pediatric department in Tsevie's hospital (Togo): 11 years later (1995-2006)]. Arch Pediatr 2007;14(8):1034-5. doi: 10.1016/j.arcped.2007.05.009 [published Online First: 20070621]
116. Czarkowski MP, Stefanoff P. [Rubella in Poland in 2005]. Przegl Epidemiol 2007;61(2):199-206.
117. Berner W. [Sanitary conditions, medical care and epidemiology situation of infectious diseases in Lvov in the period of Galicia autonomy (from the years 60ties/70ties of 19th century to the year 1914)]. Przegl Epidemiol 2007;61(4):815-25.
118. Stefanoff P. [Mumps in Poland in 2004]. Przegl Epidemiol 2006;60(3):389-92.
119. Luque C, Cisternas FA, Araya M. [Changes in the patterns of disease after the epidemiological transition in health in Chile, 1950-2003]. Rev Med Chil 2006;134(6):703-12. doi: 10.4067/s0034-98872006000600005 [published Online First: 20060814]
120. Hohendahl J, Peters N, Hüttermann U, et al. [Measles and mumps antibody concentrations in newborns and their mothers--follow up first year of life]. Klin Padiatr 2006;218(4):213-20. doi: 10.1055/s-2005-836810
121. Diawara A, Sangho H, Sango H, et al. [Morbidity and mortality of infectious diseases determined mass vaccination in children under 5 ans in Bamako District]. Mali Med 2006;21(4):8-11.
122. Czarkowski MP, Stefanoff P. [Rubella in Poland in 2004]. Przegl Epidemiol 2006;60(3):393-400.
123. Czarkowski MP, Kondej B, Paweł S. [Measles in Poland in 2004]. Przegl Epidemiol 2006;60(3):383-8.
124. Berger M, Schärer M, Kolyvanos Naumann U, et al. [Rubeola]. Praxis (Bern 1994) 2006;95(29-30):1111-9. doi: 10.1024/0369-8394.95.29.1111
125. Bégué P. [Varicella and zoster vaccines]. Virologie (Montrouge) 2006;10(6):407-14. doi: 10.1684/vir.2011.8640
126. Alessandri C, Calvani M, Jr. [Egg allergy]. Minerva Pediatr 2006;58(2):167-82.
127. Zingg W. [Does vaccination cause disease?]. Ther Umsch 2005;62(10):665-74. doi: 10.1024/0040-5930.62.10.665
128. Pogorzelska M, Ołdak E, Sulik A. [Mumps--still actual epidemiological problem in Poland]. Przegl Epidemiol 2005;59(4):841-9.
129. Oomura M, Nishi K, Terai T, et al. [A case of primary HIV infection presenting as meningoencephalitis]. Rinsho Shinkeigaku 2005;45(10):754-7.
130. Lindlbauer-Eisenach U. [120 fatal brain complications. "Measles is not a harmless pediatric illness"]. MMW Fortschr Med 2005;147(16):13.
131. Lim HS. [Changing patterns of communicable diseases in Korea]. J Prev Med Public Health 2005;38(2):117-24.
132. Furukawa M. [Factor analysis of attributive determinants for life expectancy and infant mortality rate with recipient country data in consideration of socioeconomic environment]. Nihon Eiseigaku Zasshi 2005;60(3):335-44. doi: 10.1265/jjh.60.335
133. Bakuzanashvili Kh N, Dzhavakhadze MV. [Structural and ultrastructural changes in thrombocytes of patients with measles before admittance to the hospital and after discharge]. Georgian Med News 2005(123):28-30.
134. Zverev VV, Iuminova NV. [Issues related to rubella, measles and epidemic parotiditis in the Russian Federation]. Vopr Virusol 2004;49(3):8-11.
135. Zieliński A, Czarkowski MP. [Infectious diseases in Poland in 2002]. Przegl Epidemiol 2004;58(1):9-19.
136. Sauerbrei A, Prager J, Bischoff A, et al. [Antibodies against vaccine-preventable diseases in pregnant women and their offspring. Measles, mumps, rubella, poliomyelitis, and varicella]. Bundesgesundheitsblatt Gesundheitsforschung Gesundheitsschutz 2004;47(1):10-5. doi: 10.1007/s00103-003-0689-z
137. Rümke HC, Visser HK. [Childhood vaccinations anno 2004. I. Effectiveness and acceptance of the Dutch National Vaccination Programme]. Ned Tijdschr Geneeskd 2004;148(8):356-63.
138. Rümke HC, Visser HK. [Childhood vaccinations anno 2004. II. The real and presumed side effects of vaccination]. Ned Tijdschr Geneeskd 2004;148(8):364-71.
139. Onishchenko GG. [On the epidemiological situation in quarantine, natural focal and other infections on the territory of the Southern Federal District]. Zh Mikrobiol Epidemiol Immunobiol 2004(3):23-30.
140. Arrivé E, Perez F, Pierre LM. [The integrated management of childhood illness: Haiti's example]. Sante 2004;14(3):137-42.

141. Zieliński A, Czarkowski MP. [Infectious diseases in Poland in 2001]. Przegl Epidemiol 2003;57(1):9-17.
142. Uloha AI, Lialikau SA. [The relationship between insulin-dependent diabetes mellitus and acute infections in children]. Endokrynol Diabetol Chor Przemiany Materii Wieku Rozw 2003;9(2):73-6.
143. Tomoda A, Nomura K, Shiraishi S, et al. [Trial of intraventricular ribavirin and interferon-alpha combination therapy for subacute sclerosing panencephalitis (SSPE) in Japan]. No To Hattatsu 2003;35(4):321-6.
144. Takayama N, Suganuma A. [Clinical investigation on adult inpatients contracted measles; comparing with pediatric measles inpatients]. Kansenshogaku Zasshi 2003;77(10):815-21. doi: 10.11150/kansenshogakuzasshi1970.77.815
145. Takayama N. [Change in the age-distribution of measles patients admitted to our hospital from 1981 to 2002]. Kansenshogaku Zasshi 2003;77(7):488-92. doi: 10.11150/kansenshogakuzasshi1970.77.488
146. Stampi S, Matteini P, Zanetti F, et al. [Immigration and health: observational study concerning the foreign children attending the Bologna community pediatric service]. Ann Ig 2003;15(3):261-70.
147. Orne-Gliemann J, Perez F, Leroy V, et al. [A decade of child health research in developing countries]. Sante 2003;13(2):69-75.
148. Mabiala-Babela JR, Samba-Louaka C, Mouko A, et al. [Morbidity in a pediatric department (University Hospital of Brazzaville): 12 years later (1989-2001)]. Arch Pediatr 2003;10(7):650-2. doi: 10.1016/s0929-693x(03)00290-2
149. Lassaletta Atienza A, Cebrero García M, Martino Alba R, et al. [Subacute sclerosing panencephalitis: a still existing disease]. An Pediatr (Barc) 2003;58(6):594-8. doi: 10.1016/s1695-4033(03)78127-2
150. Kreidl P, Morosetti G. [Must we expect an epidemic of measles in the near future in Southern Tyrol?]. Wien Klin Wochenschr 2003;115 Suppl 3:55-60.
151. Jedrychowski W, Maugeri U, Jedrychowska-Bianchi I. [Susceptibility to respiratory infections in school children after measles. Results of epidemiological study in Krakow]. Przegl Epidemiol 2003;57(3):421-9.
152. Janaszek-Seydlitz W, Bucholc B, Wysokińska T, et al. [Prevalence of antibodies and vaccination against measles, mumps and rubella in Polish population]. Przegl Epidemiol 2003;57(2):281-8.
153. Eseverri JL, Ranea S, Marin A. [Adverse reactions to vaccines]. Allergol Immunopathol (Madr) 2003;31(3):125-38. doi: 10.1016/s0301-0546(03)79278-7
154. del Buono MB, Vaschetti P, Iannicelli J, et al. [Age-related loss of maternal antibodies against measles in children in La Plata]. Rev Argent Microbiol 2003;35(2):102-5.
155. Avila Castañón L, Pérez López J, Rosas Vargas MA, et al. [The response to PPD and its relation to allergic diseases in children vaccinated at birth with BCG]. Rev Alerg Mex 2003;50(2):48-53.
156. [New goal for vaccination programs in the Region of the Americas: to eliminate rubella and congenital rubella syndrome. 2003]. Rev Panam Salud Publica 2003;14(5):359-63.
157. Tsutsumi H. [Viral infection]. Nihon Rinsho 2002;60(3):585-90.
158. Terada K, Niizuma T, Ogita S, et al. [Alterations in epidemics and vaccination for measles during a 20 year period and a strategy for elimination in Kurashiki City, Japan]. Kansenshogaku Zasshi 2002;76(3):180-4. doi: 10.11150/kansenshogakuzasshi1970.76.180
159. Orie NG. [The relationship between asthma and chronic obstructive and restrictive pulmonary diseases; lessons from the past century]. Ned Tijdschr Geneeskd 2002;146(32):1504-8.
160. Mazurek J, Czarkowski MP. [Infectious diseases in Poland in 2000]. Przegl Epidemiol 2002;56(2):217-25.
161. Lucena R, Gomes I, Nunes L, et al. [Clinical and laboratory features of aseptic meningitis associated with measles-mumps-rubella vaccine]. Rev Panam Salud Publica 2002;12(4):258-61. doi: 10.1590/s1020-49892002001000006
162. Genton B. [Vaccines against poverty]. Rev Med Suisse Romande 2002;122(12):581-4.
163. Faraj H, Schakal A, Haddad E, et al. [Bilateral retinitis: early manifestation of subacute sclerosing panencephalitis. A case report]. J Fr Ophtalmol 2002;25(9):944-8.
164. Castillo-Solórzano C, de Quadros CA. [Accelerated rubella control and the prevention of congenital rubella syndrome]. Rev Panam Salud Publica 2002;11(4):273-6. doi: 10.1590/s1020-49892002000400017
165. [Vaccination deficits in Germany. Still fatalities caused by measles]. MMW Fortschr Med 2002;144(14):54.
166. Wang L, Sun L, Jiang Z, et al. [The study of countermeasures on measles control in infants]. Zhonghua Liu Xing Bing Xue Za Zhi 2001;22(1):49-50.
167. van den Hof S, van den Kerkhof JH, ten Ham PB, et al. [Measles epidemic in the Netherlands, 1999-2000]. Ned Tijdschr Geneeskd 2001;145(52):2529-33.
168. Terada K, Niizuma T, Ogita S, et al. [Outbreak of measles in a hospital and measures taken against hospital infection--evidence of cost and benefits]. Kansenshogaku Zasshi 2001;75(6):480-4. doi: 10.11150/kansenshogakuzasshi1970.75.480
169. Tall F, Ki-Zerbo G, Ouedraogo I, et al. [Noma in children in a hospital environment in Bobo-Dioulasso: epidemiologic, clinical and management aspects]. Odontostomatol Trop 2001;24(96):21-5.
170. Mihălţan F, Ulmeanu R. [Vaccines--their role and implications in respiratory pathology]. Pneumologia 2001;50(3):167-70.
171. Marck KW, Spijkervet FK. [Noma: 'the face of poverty']. Ned Tijdschr Tandheelkd 2001;108(12):496-9.
172. Lytkina IN, Filatov NN, Mironova VF, et al. [Measles: prospects of vaccine prophylaxis and decrease of morbidity in Moscow]. Zh Mikrobiol Epidemiol Immunobiol 2001(1):25-8.
173. Lehrnbecher T. [Intravenous immunoglobulins in the prevention of infection in children with hematologic-oncologic diseases]. Klin Padiatr 2001;213 Suppl 1:A103-5. doi: 10.1055/s-2001-17506
174. Kissani N, Ouazzani R, Belaidi H, et al. [Epileptic seizures and epilepsy in subacute sclerosing panencephalitis (report of 30 cases]. Neurophysiol Clin 2001;31(6):398-405. doi: 10.1016/s0987-7053(01)00278-7
175. Dubus JC, Bosdure E, Mates M, et al. [Virus and respiratory allergy in children]. Allerg Immunol (Paris) 2001;33(2):78-81.

176. Yang S, Zhou F. [Analysis of clinical epidemiology for 208 patients of measles and some lessons learned]. Zhonghua Liu Xing Bing Xue Za Zhi 2000;21(5):352-4.
177. Valenzuela MT, O'Ryan M. [Achievements and challenges of the expanded immunization program in the Americas]. Rev Med Chil 2000;128(8):911-22.
178. Skachkov MV. [Specific features of the epidemic process of measles and epidemic parotitis in territories with ecologically unfavorable conditions]. Zh Mikrobiol Epidemiol Immunobiol 2000(4 Suppl):124-6.
179. Rinka H, Ujino H, Miyachi T, et al. [Studies on surfactant replacement therapy in pediatric measles pneumonitis]. Masui 2000;49(4):396-403.
180. Naruszewicz-Lesiuk D. [Measles in 1998]. Przegl Epidemiol 2000;54(1-2):35-44.
181. Desgrandchamps D, Schaad UB, Glaus J, et al. [Seroprevalence of IgG antibodies against measles, mumps and rubella in Swiss children during the first 16 months of life]. Schweiz Med Wochenschr 2000;130(41):1479-86.
182. de Meer K, Roord JJ. [Favorable effects of vitamin A in measles infection]. Ned Tijdschr Geneeskd 2000;144(6):287-8.
183. Cabezas C, Ramos F, Vega M, et al. [IMPACT OF THE IMMUNIZATION PROGRAM INTEGRATED TO THE EXPANDED IMMUNIZATION PROGRAM(EPI) IN HUANTA,1994-1997]. Rev Gastroenterol Peru 2000;20(3):201-12.
184. Bolanda D, Talani P, Nzaba P, et al. [Survey of measles vaccine coverage in Brazzaville]. Bull Soc Pathol Exot 2000;93(2):123-4.
185. Naruszewicz-Lesiuk D. [Measles in 1997]. Przegl Epidemiol 1999;53(1-2):13-22.
186. Malvy D. [Micronutrients and tropical viral infections: one aspect of pathogenic complexity in tropical medicine]. Med Trop (Mars) 1999;59(4 Pt 2):442-8.
187. Kawasaki Y, Hosoya M, Katayose M, et al. [The efficacy of oral vitamin A supplementation for measles and respiratory syncytial virus (RSV) infection]. Kansenshogaku Zasshi 1999;73(2):104-9. doi: 10.11150/kansenshogakuzasshi1970.73.104
188. Isa MB, González M, Martínez LC, et al. [Measles outbreak in the province of Córdoba, Argentina, in 1998]. Rev Argent Microbiol 1999;31(2):90-5.
189. Gutiérrez G, Reyes H, Fernández S, et al. [Impact of health services, sanitation and literacy in the mortality of children under 5 years of age]]. Salud Publica Mex 1999;41(5):368-75.
190. da Silva AA, Gomes UA, Tonial SR, et al. [Vaccination coverage and risk factors associated to non-vaccination in a urban area of northeastern Brazil, 1994]. Rev Saude Publica 1999;33(2):147-56. doi: 10.1590/s0034-89101999000200006
191. Traoré L, Banou AA, Sacko D, et al. [Strategies to control vitamin A deficiency]. Sante 1998;8(2):158-62.
192. Peñuelas Ruiz JA, Manrique Blázquez RR, Diestro Contreras A, et al. [An evaluation of the surveillance system of mandatory disease reporting]. Aten Primaria 1998;22(2):85-91.
193. Naruszewicz Lesiuk D. [Measles in 1996]. Przegl Epidemiol 1998;52(1-2):13-21.
194. Mihalache D, Luca V, Luca C, et al. [The clinico-evolutionary considerations of 492 cases of measles hospitalized in the Infectious Disease Clinic Iaşi in 1997]. Rev Med Chir Soc Med Nat Iasi 1998;102(3-4):114-6.
195. Klimkiewicz A, Müller-Schulz M, Gerigk C, et al. [Fatal course of measles infection in a patient with a low-grade malignant non-Hodgkin lymphoma]. Dtsch Med Wochenschr 1998;123(30):901-4. doi: 10.1055/s-2007-1024096
196. Janaszek W. [The impact of immunization against measles on epidemiology of measles in Poland]. Przegl Epidemiol 1998;52(4):413-25.
197. Iushchuk ND, Frolov VM, Ershova IB. [Association of HLA antigens, classes I and II, with measles morbidity]. Zh Mikrobiol Epidemiol Immunobiol 1998(3):55-7.
198. Inui N, Chida K, Suda T, et al. [Pulmonary cryptococcosis exhibiting diffuse multiple nodular shadows]. Nihon Kokyuki Gakkai Zasshi 1998;36(12):1038-42.
199. Gałazka A, Kraigher A, Robertson SE. [Wide-spread inflammation of the parotid glands (mumps): an underestimated disease. II. Development, use, efficacy and safety of mumps vaccines]. Przegl Epidemiol 1998;52(4):401-12.
200. Zhang LZ, Yan SH, Meng XK. [Discussion on the elimination of measles based on the 45-year-incidence data in Inner Mongolia]. Zhonghua Liu Xing Bing Xue Za Zhi 1997;18(5):263-6.
201. Wölfle J, Schmidt J, Kreft B, et al. [Subacute sclerosing panencephalitis (SSPE) as differential diagnosis in severe personality changes and ataxia--case report and literature review]. Klin Padiatr 1997;209(3):111-5. doi: 10.1055/s-2008-1043938
202. Shinjo H, Ueki A, Miwa C, et al. [A case of chronic encephalitis due to double infection with herpes simplex and measles viruses]. No To Shinkei 1997;49(11):1021-6.
203. Romanenko NA, Sergiev VP, Chernyshenko AI, et al. [New approaches to the eradication of enterobiasis in children]. Med Parazitol (Mosk) 1997(1):3-5.
204. Onishchenko GG. [Illness rate with viral infections in the Russian Federation]. Vopr Virusol 1997;42(4):148-52.
205. Milewska D, Kulczycki J, Sobczyk W. [Computerized analysis of the database of SSPE patients treated at the 1st Neurological Clinic of the Institute of Psychiatry and Neurology in Warsaw in 1978-1995]. Neurol Neurochir Pol 1997;31(3):475-91.
206. Matter L. [Vaccinations: the necessary and the desirable]. Schweiz Med Wochenschr 1997;127(10):377-81.
207. Luthi JC, Kessler W, Boelaert M. [A survey on vaccine efficacy in the city of Bongor (Chad) and its operational consequences for the vaccination program]. Bull World Health Organ 1997;75(5):427-33.
208. Kuate Defo B. [Causes and determinants of mortality under 2 years of age in subSaharan Africa: application of concurrent risk models]. Cah Que Demogr 1997;26(1):3-39.
209. Ion-Nedelcu N, Ciufecu C. [The measles epidemic in 1996-1997 in Romania: an analysis of surveillance data. The options for the control of measles epidemic contingencies in a highly vaccinated population]. Bacteriol Virusol Parazitol Epidemiol 1997;42(4):249-56.
210. Gendrel D. [Measles and rubella]. Rev Prat 1997;47(13):1434-7.

211. Bronzwaer SL, de Groot CJ. [Risk factors for a complicated disease course in children with measles admitted to a Philippine university hospital]. Ned Tijdschr Geneeskd 1997;141(51):2492-5.
212. Villagrana-Zesati R, Figueroa-Damián R, Santamaría-Corona H, et al. [Measles and pregnancy. Evolution and management]. Ginecol Obstet Mex 1996;64:459-62.
213. van der Meer H, Kimpen JL. [Insufficient vaccination status of children with a chronic disease]. Ned Tijdschr Geneeskd 1996;140(27):1402-6.
214. Rykushin Iu P. [The strategy for vaccinal prophylaxis and the elimination of measles in the developed countries]. Zh Mikrobiol Epidemiol Immunobiol 1996(5):44-8.
215. Vitte S, Lawani R, Bouat C, et al. [Xerophthalmia: current data]. Med Trop (Mars) 1995;55(4 Pt 2):434-8.
216. Takechi S, Nishio S, Yokoyama M, et al. [Clean intermittent catheterization in neurogenic bladder patients with vesicoureteral reflux]. Nihon Hinyokika Gakkai Zasshi 1995;86(10):1520-4. doi: 10.5980/jpnjurol1989.86.1520
217. Schlumberger M, Sawadogo I, Sanou C, et al. [Description and survey of a measles epidemic in Burkina Faso]. Sante 1995;5(1):31-5.
218. Matter HC, Cloetta J, Zimmermann H. [The Sentinella reporting system in Switzerland exemplified by pertussis monitoring from 1991 to 1993. Sentinel Work Group]. Praxis (Bern 1994) 1995;84(23):690-7.
219. Liu BY, Feng ZX, Xu AQ. [A study on the level of antibody against measles through maternal-fetal transfer and the immuno-response to measles vaccine among 4 to 7 month olds]. Zhonghua Liu Xing Bing Xue Za Zhi 1995;16(5):263-5.
220. Kyei-Faried S, Hermans M. [Primary health care in Ghana: no pay no cure?]. Ned Tijdschr Geneeskd 1995;139(45):2321-5.
221. Kunika N, Yasaki S, Oshima J, et al. [Serial changes of MRI and SPECT findings in a case of adult-onset SSPE]. Rinsho Shinkeigaku 1995;35(11):1214-20.
222. González-Ferrer S, Pineda-Del Villar L, Brito-Brito J, et al. [Hereditary diseases and congenital malformations at the Unit of Medical Genetics of the University of Zulia. Years: 1983-1992]. Invest Clin 1995;36(2):47-60.
223. Eono P, Migliani R, Philippe B, et al. [Burundi: humanitarian mission (January-April 1994)]. Med Trop (Mars) 1995;55(2):172-7.
224. Bouvier-Colle MH. [Sex-specific differences in mortality after high-titre measles vaccination]. Rev Epidemiol Sante Publique 1995;43(1):97.
225. Barennes H, Banos MT, Garba D. [Malnutrition and health status of children under five years old in the suburban zone of Niamey, Niger]. Med Trop (Mars) 1995;55(2):139-42.
226. van der Zwan CW, Plantinga AD, Rümke HC, et al. [Measles in The Netherlands; epidemiology and the effect of vaccination]. Ned Tijdschr Geneeskd 1994;138(48):2390-5.
227. Krawinkel MB. [Child health in our world]. Z Ernahrungswiss 1994;33(4):248-57. doi: 10.1007/bf01614430
228. Horibe K, Katayama I, Miyajima Y, et al. [Outcome of treatment protocol 8704T for childhood T cell leukemia and lymphoma]. Rinsho Ketsueki 1994;35(7):657-64.
229. Hayashi Y, Taku K, Nitta E, et al. [A case of subacute sclerosing panencephalitis treated with intraventricular interferon--the side effects of interferon-alpha to the central nervous system]. Rinsho Shinkeigaku 1994;34(1):80-2.
230. Guarnaccia S, Muraro MA, Aparicio C, et al. [The inhalational therapy of respiratory pathology]. Pediatr Med Chir 1994;16(5):423-7.
231. Frenette L. [Vaccination in Togo]. Can Nurse 1994;90(5):43-5.
232. Cutts FT, Dabis F. [Measles control in developing countries]. Sante 1994;4(3):163-71.
233. Cazacu E, Bălan A, Dinică V. [The evolution of measles morbidity and mortality in Romania after the introduction of antimeasles vaccination]. Bacteriol Virusol Parazitol Epidemiol 1994;39(1-2):35-44.
234. Casanova-Cardiel LJ, Hermida-Escobedo C. [Measles in the young adult. Clinical features of 201 cases]. Rev Invest Clin 1994;46(2):93-8.
235. Bezuglova MS, Rusakova EV, Drynov ID, et al. [The use of the results of a statistical analysis for the goal-directed correction of epidemic-control measures with respect to measles]. Zh Mikrobiol Epidemiol Immunobiol 1994(2):45-9.
236. Wagner S, Jung H, Nau F, et al. [Relevance of infectious diseases in a pediatric practice]. Klin Padiatr 1993;205(1):14-7. doi: 10.1055/s-2007-1025190
237. Rykushin Iu P. [An epidemiological assessment of the protection against measles by population age groups in the Russian Federation]. Zh Mikrobiol Epidemiol Immunobiol 1993(4):56-62.
238. Reinert P. [Infections in malnourished infants and children]. Dev Sante 1993(103):4-6.
239. Obengui P, Nkoua JL, Moyen G, et al. [Value of a mass vaccination campaign: measles cases in the hospital milieu in Brazzaville (Congo)]. Bull Soc Pathol Exot 1993;86(4):243-4.
240. Mimra J, Farník J. [The epidemic process in infections before and after active immunization. I. Comparison of long-term monitoring of the disease with a simple mathematical model]. Cesk Epidemiol Mikrobiol Imunol 1993;42(1):3-6.
241. Kobayashi A, Yoda Y, Yang JY, et al. [Transient pure red cell aplasia in an adult with acute parvovirus B19 infection: observation of PVB19 DNA by polymerase chain reaction, viral antibody and erythroid cells in the bone marrow]. Rinsho Ketsueki 1993;34(4):484-9.
242. Grilli G, Cimini D, Morbidelli L, et al. [Transmission of measles during four outbreaks in a partially vaccinated population]. Minerva Pediatr 1993;45(1-2):21-8.
243. Albalá C, Vio F, Robledo A, et al. [The epidemiological transition in Chile]. Rev Med Chil 1993;121(12):1446-55.
244. Yang G. [Cause analysis of measles incidence in National Diseases Surveillance System in 1990]. Zhonghua Liu Xing Bing Xue Za Zhi 1992;13(2):79-81.
245. Terrero C, Demorizi JA, Feris JM, et al. [Incidence of measles with complications]. Arch Domin Pediatr 1992;28(2-3):31-4.

246. Sejda J, Prívorová A, Benes C. [The present epidemiologic status and prognosis in measles]. Cesk Epidemiol Mikrobiol Imunol 1992;41(4):210-4.
247. Ramaroson SR, Rakotomanga JD. [Causes of mortality in a rural area in the Faritany of Toamasina in 1986]. Ravintsara 1992(4):31-48.
248. Nasuhara Y, Yamazaki K, Takaoka K, et al. [A case of Swyer-James syndrome with bilateral lesions]. Nihon Kyobu Shikkan Gakkai Zasshi 1992;30(3):495-9.
249. Naruszewicz-Lesiuk D. [Measles--1990]. Przegl Epidemiol 1992;46(1-2):43-50.
250. Morales Suárez-Varela MM, Cotanda Gutiérrez P, Asensi Botet F, et al. [Epidemiologic study of 245 cases of children hospitalized with measles]. Aten Primaria 1992;10(5):778-80.
251. Magos-López C, Sánchez-Villarreal F, Gutiérrez G, et al. [The National Serum Bank]. Salud Publica Mex 1992;34(2):136-47.
252. Kourouma K, Konde MK, Diallo MP, et al. [Vaccination against measles at 6 months of age]. Ann Pediatr (Paris) 1992;39(9):566-71.
253. Davis R. [Measles control in the 1990s: perspectives of prevention and eradication with special reference to Africa]. Med Trop (Mars) 1992;52(3):285-9.
254. Bocket L, Delforge F, Wattré P, et al. [Value of interferon alpha determination in the diagnosis of meningoencephalitis presumed to be of viral origin]. Rev Med Interne 1992;13(1):27-31. doi: 10.1016/s0248-8663(05)80007-4
255. [The living and health conditions of Palestinian children]. Riv Inferm 1992;11(4):247-52.
256. [Vitamin A in the treatment of measles]. Arch Latinoam Nutr 1992;42(2):211-4.
257. Tabutin D. [African population growth: status and prospects]. Tiers Monde 1991;32(125):159-73.
258. Strauss J, Mrázová M, Macátová I, et al. [Loss of maternal measles antibodies acquired by vaccination against measles]. Cesk Epidemiol Mikrobiol Imunol 1991;40(3):137-43.
259. Rusakova EV, Bezuglova MS, Vasil'eva VI, et al. [The validation of the expediency of a review of the times for revaccinating children against measles]. Zh Mikrobiol Epidemiol Immunobiol 1991(8):51-4.
260. Rey JL, Lhuillier M, Dem M, et al. [The transmission of maternal measles antibodies to newborn infants in the Ivory Coast]. Med Trop (Mars) 1991;51(2):215-8.
261. Perović S, Peić K, Lazar F, et al. [Rubeola in the population of Subotica 1978-1989]. Med Pregl 1991;44(3-4):139-41.
262. Naruszewicz-Lesiuk D. [Measles--1989]. Przegl Epidemiol 1991;45(1-2):25-30.
263. Matsumoto I, Yoshida S, Kawana R. [Virological surveillance of acute respiratory tract illnesses of children in Morioka, Japan. II. Rhinovirus infection]. Kansenshogaku Zasshi 1991;65(10):1286-96. doi: 10.11150/kansenshogakuzasshi1970.65.1286
264. Gürtler L, Jilg W, Kraus HK. [Possible long-term prognosis in epidemiologically significant virus infections]. Versicherungsmedizin 1991;43(2):44-8.
265. Gendrel D, Kombila M, Richard-Lenoble D. [Placental transfer of antibodies in Africa and protection of the newborn]. Bull Soc Pathol Exot 1991;84(5 Pt 5):441-7.
266. Cervenka J, Kosecká G. [Retrospective epidemiologic analysis of the occurrence of measles in Slovakia]. Cesk Epidemiol Mikrobiol Imunol 1991;40(3):144-57.
267. Cazes MH. [Demographic and evolutionary dynamics of an isolate: the Dogon of Boni]. Etudes Mali 1991(44):57-64.
268. Bezuglova MS, Rusakova EV, Frank KD, et al. [The use of computers and mathematical methods for optimizing epidemic control measures with respect to measles infection]. Zh Mikrobiol Epidemiol Immunobiol 1991(9):44-7.
269. Zi W. [Monetary incentives for a more effective immunization program]. Foro Mund Salud 1990;11(2):173-8.
270. Velázquez-Monroy OJ, Alvarez-Lucas CH, Lezana-Fernández MA, et al. [Epidemiologic overview of measles in Mexico: current situation and perspectives]. Bol Med Hosp Infant Mex 1990;47(7):462-73.
271. Van der Veken J. [Measles surveillance in Belgium 1982-1989]. Acta Clin Belg 1990;45(6):379-85.
272. Sobaniec-Lotowska M, Sobaniec W, Sulkowski S, et al. [Clinico-morphologic analysis of subacute sclerosing panencephalitis in patients up to the 19th year of life]. Pol Tyg Lek 1990;45(27-28):549-52.
273. Sliusar LI, Sokhin AA, Radomskaia FS, et al. [The results of multiyear observations on the duration of the maintenance of immunity in those vaccinated and revaccinated against and recovered from measles]. Zh Mikrobiol Epidemiol Immunobiol 1990(8):66-70.
274. Shakhanina IL, Ivlieva OM, Narkevich MI. [Infectious disease mortality in the USSR]. Zh Mikrobiol Epidemiol Immunobiol 1990(8):53-7.
275. Serra I, Romero MI. [Measles in Chile]. Rev Med Chil 1990;118(2):214-24.
276. Sada-Díaz E, Pasquel-García P, Narváez O, et al. [Pneumonia caused by measles: description of a clinical case of giant cell pneumonia in a non-immunosuppressed adolescent patient]. Bol Med Hosp Infant Mex 1990;47(7):524-7.
277. Navarrete-Navarro S, Avila-Figueroa C, Ruiz-Gutiérrez E, et al. [Nosocomial measles: a proposal for its control in hospitals]. Bol Med Hosp Infant Mex 1990;47(7):495-9.
278. Naruszewicz-Lesiuk D. [Measles--1988]. Przegl Epidemiol 1990;44(1-2):25-32.
279. N'Guessan Diplo L, Rey JL, Soro B, et al. [Infant mortality and its causes in a sub-district of the Ivory Coast]. Med Trop (Mars) 1990;50(4):429-32.
280. Longini IM, Jr., Haber MJ, Halloran ME. [Direct and indirect effects of vaccines: an annotation on the estimation of the vaccine efficacy from outbreaks caused by acute infection agents such as measles]. Bol Med Hosp Infant Mex 1990;47(7):516-9.
281. Litwińska B, Bucholc B, Sadowski W, et al. [Antiviral activity of commercial immunoglobulin preparations]. Med Dosw Mikrobiol 1990;42(1-2):89-94.
282. Lasch EE. [Child health in a developing population with changing patterns of belief (Gaza 1973-1983)]. Harefuah 1990;118(9):522-5.

283. Kostinov MP, Gervazieva VB, Balabolkin, II. [Immunologic reactivity of children with allergic diseases during the measles vaccinal process]. Zh Mikrobiol Epidemiol Immunobiol 1990(3):81-5.
284. Ion-Nedelcu N, Mihăileanu C, Muşceleanu M, et al. [An estimate of the index of collective measles immunity among the pupils in 8-year elementary education in 1988-1989 in sector III of the city of Bucharest]. Bacteriol Virusol Parazitol Epidemiol 1990;35(2):119-26.
285. Iakovleva LS, Bogomolova NN. [A mixed infection of lymphoid cell lines by the human T-lymphotropic retrovirus type I (HTLV-I) and the measles vaccine virus]. Vopr Virusol 1990;35(3):209-11.
286. Hane AA, Badiane M, Wade A, et al. [Pleuropneumopathies in children in an infectious pathology department in Dakar. Epidemiological, clinical and radiological features. Apropos of 150 cases]. Dakar Med 1990;35(2):133-40.
287. Furby A, Vallée L, Rousseaux M, et al. [Prolonged remission in subacute sclerosing panencephalitis: 2 cases]. Rev Neurol (Paris) 1990;146(3):191-5.
288. Fernández de Castro J, Kumate J. [Vaccination against measles. The situation in Mexico and America. Advances in the method of aerosol immunization]. Bol Med Hosp Infant Mex 1990;47(7):449-61.
289. Díaz-Ortega JL, Valdespino-Gómez JL, Zárate-Aquino ML, et al. [Immuno-epidemiologic considerations on the prevention of measles in Mexico]. Bol Med Hosp Infant Mex 1990;47(7):474-81.
290. Clements CJ, Kane M, Hu DJ, et al. [Hepatitis B vaccine: a new force against pandemic diseases]. Foro Mund Salud 1990;11(2):165-8.
291. Avila-Figueroa C, Navarrete-Navarro S, Santos JI. [Guidelines for vaccination against measles in children]. Bol Med Hosp Infant Mex 1990;47(7):528-33.
292. Avila-Figueroa C, Navarrete-Navarro S, Martínez-Aguilar M, et al. [Complications in children with measles]. Bol Med Hosp Infant Mex 1990;47(7):520-3.
293. Ania Lafuente BJ, Jiménez Mesa C, Gago García C, et al. [Detection of epidemics from the Compulsory Disease Report. The case of measles in Las Palmas]. Rev Sanid Hig Publica (Madr) 1990;64(9-10):561-9.
294. Amaro Labrador J, Gato Chamizo I, Venero Hernández ML. [Mumps and rubella morbidity]. Rev Cubana Enferm 1990;6(1):118-27.
295. [Network of researchers on the health aspects of reproduction]. Vie Sante 1990(4):18-21.
296. Tische A, Gerike E, Strauss J, et al. [Immunoglobulin specific and conventional methods in the serodiagnosis of measles]. Z Gesamte Hyg 1989;35(6):367-9.
297. Suganuma M, Sayama Y, Nakamura M. [Trypan blue staining capacities of the culture cells (2)--The mechanism of staining]. Rinsho Byori 1989;37(12):1361-6.
298. Sahuguède P, Roisin A, Sanou I, et al. [An epidemic of measles in Burkina Faso: 714 cases hospitalized in the hospital at Bobo-Dioulasso. A study of risk factors]. Ann Pediatr (Paris) 1989;36(4):244-51.
299. Rozov AA, Makhlina VS, Makarovskaia NV. [Child mortality in the developing countries of Africa]. Sov Zdravookhr 1989(3):58-63.
300. Ranchov GK. [Quantitative research on the cyclic fluctuations of the epidemic process in a number of infectious diseases in Bulgaria]. Zh Mikrobiol Epidemiol Immunobiol 1989(9):59-62.
301. Mbodj FG. [Malaria and diarrhea, principal causes of infant deaths]. Pop Sahel 1989(10):10-1.
302. Kostinov MP, Balabolkin, II, Igna'teva GV, et al. [Characteristics of the clinical course of the post-vaccination period and the development of anti-measles immunity in children with allergic diseases]. Pediatriia 1989(9):33-8.
303. Goulet V, Papasoglou S. [Evaluation of coverage under the national plan for measles and rubella vaccination based on a sample of schools]. Ann Pediatr (Paris) 1989;36(1):43-8.
304. Fargues P. [The month of birth: a factor of inequality before death]. Pop Sahel 1989(10):20-4.
305. Egbagbe S. [Child mortality in the countries of Western Africa]. Sov Zdravookhr 1989(7):51-5.
306. Delacollette C, Van der Stuyft P, Molima K, et al. [Study of general mortality and of mortality related to malaria in the mountains of Kivu, Zaire]. Rev Epidemiol Sante Publique 1989;37(2):161-6.
307. Col JY. [Delivery in France of patients born in Black Africa]. Rev Fr Gynecol Obstet 1989;84(5):425-34.
308. Cárdenas Ayala VM, Ruiz Matus C, Cabrera Coello L, et al. [Estimate of the cost/benefit ratio of measles vaccination]. Salud Publica Mex 1989;31(6):735-44.
309. Campanella N, Tarantini F. [Health care organization and health in a region of Zaire]. Ann Ig 1989;1(6):1389-417.
310. Bogomolova NN, Burgasova MP, Chaplygina NM, et al. [Experimental measles infection in rodents]. Vopr Virusol 1989;34(5):590-5.
311. Baya B. [High infant mortality rates that can be substantially lowered]. Pop Sahel 1989(10):6-9.
312. Ntilivamunda A. [Expanded Programme on Immunization: status and perspectives]. Imbonezamuryango 1988(13):29-34.
313. Loras-Duclaux I, David L, Peyramond D, et al. [Epidemiological study and cost evaluation of measles in Lyons hospitals over a 5-year period]. Pediatrie 1988;43(5):451-4.
314. Kuate Defo B. [Causes of infant-child mortality in Yaounde]. Ann IFORD 1988;12(2):65-95.
315. Kotte W, Kotte S. [Measles infections at a Mozambique provincial hospital]. Kinderarztl Prax 1988;56(7):347-51.
316. Keuzeta JJ, Merlin M, Josse R, et al. [Infant and child morbidity and mortality due to diarrheal disease in central Africa]. Ann IFORD 1988;12(1):69-87.
317. Gendrel D, Richard-Lenoble D, Blot P, et al. [Transfer of measles immunoglobulins and antibodies from mother to child in Africa and Europe]. Presse Med 1988;17(32):1633-6.
318. Gaigbe Togbe V. [Seasonality and cause of infant deaths in Yaounde]. Ann IFORD 1988;12(2):97-126.
319. Deshevoĭ SE, Rykushin Iu P, Ovsiannikova IV, et al. [Epidemiologic effectiveness of mass revaccination against measles]. Zh Mikrobiol Epidemiol Immunobiol 1988(8):69-72.

320. Benguigui Y. [Magnitude and control of acute respiratory infections in children]. Salud Publica Mex 1988;30(3):362-9.
321. Vázquez Fernández E, López Rois F, Vázquez Carrete JA, et al. [Morbidity, vaccine coverage and immunity against measles, mumps and rubella in a Gallician population from 2 to 5 years old]. An Esp Pediatr 1987;27(1):27-31.
322. Tardieu M. [Acute viral meningitis and encephalitis in children]. Pediatrie 1987;42(9):675-80.
323. Reinert P, Bernaudin F, Lobut JB, et al. [The role of IgE in recurrent ORL infections in children]. Pathol Biol (Paris) 1987;35(10 Pt 2):1446-9.
324. De Ory Manchón F, Fernández Rodríguez-Patiño MV, Echevarría Mayo JM, et al. [Antibodies against measles, rubella and parotitis in the first 2 years of life]. Rev Sanid Hig Publica (Madr) 1987;61(3-4):289-300.
325. [Public health programs have greatly reduced infant mortality in Costa Rica]. Perspect Int Planif Fam 1987(Spec No):33-4.
326. [Immunization: an important aspect often neglected]. Rumah Tangga Kesehatan 1987(8):10-1.
327. Verrotti A, Chiarelli F, Ricci F, et al. [Epidemiology of measles and its complications in Italy. Personal experience with a hospital case load]. Pediatr Med Chir 1986;8(2):209-13.
328. Venediktova N, Kolpakova VE, Kozlova SN, et al. [Immune structure of the pediatric population in relation to the mumps virus and the morbidity of serous meningitis of mumps etiology in Sverdlovsk]. Zh Mikrobiol Epidemiol Immunobiol 1986(11):31-4.
329. Sokhin AA, Sliusar LI, Ignatov SA, et al. [Effectiveness of measles immunoprophylaxis and the criteria for assessing its possible elimination]. Zh Mikrobiol Epidemiol Immunobiol 1986(11):24-30.
330. Sandow D, Nuhn P, Deutschmann K, et al. [Antiviral action of lysolecithin analogs against human pathogenic viruses]. Pharmazie 1986;41(6):404-6.
331. Roussel L. [Complications of measles in adult Africans. Apropos of 95 cases]. Med Trop (Mars) 1986;46(4):359-64.
332. Moiraghi Ruggenini A, Zotti C, Pedronetto A, et al. [Hospitalization in Turin for measles and related complications 1973-1983: medical and economic evaluation in relation to the opportunity for vaccination intervention]. Boll Ist Sieroter Milan 1986;65(6):502-11.
333. Litvinov SK, Lobanov AV, Peregudov AN. [The cluster method in conducting epidemiological research]. Zh Mikrobiol Epidemiol Immunobiol 1986(11):78-84.
334. Guiscafré-Gallardo H. [Measles: persistence of a problem]. Bol Med Hosp Infant Mex 1986;43(9):523-5.
335. Fassin D. [The good mother: rural and urban practices related to measles in Haalpulareen women in Senegal]. Soc Sci Med 1986;23(11):1121-9. doi: 10.1016/0277-9536(86)90329-1
336. Duflo B, Balique H, Ranque P, et al. [Estimation of the impact of the principal diseases in rural Mali]. Rev Epidemiol Sante Publique 1986;34(6):405-18.
337. Bolotovskiĭ VM, Gelikman BG, Mikheev IV, et al. [Measles morbidity among children inoculated against this infection]. Zh Mikrobiol Epidemiol Immunobiol 1986(3):38-43.
338. Xü FG. [Measles & rubella hemagglutination inhibition antibodies in women of child-bearing age and the time of decline of maternally acquired antibodies among neonates and infants]. Zhonghua Liu Xing Bing Xue Za Zhi 1985;6(1):9-11.
339. Valdespino Gómez JL, Zárate Aquino ML, Domínguez Brito E, et al. [Antimeasles passive natural immunity in infants in Mexico City]. Salud Publica Mex 1985;27(6):524-31.
340. Ntilivamunda A. [Program to combat communicable diseases in children]. Imbonezamuryango 1985(3):50-2.
341. Litvinov SK, Henderson RH, Galazka AM, et al. [The Expanded Programme on Immunization: the results of its realization, problems and outlook]. Zh Mikrobiol Epidemiol Immunobiol 1985(2):114-20.
342. Le Gonidec G. [The expanded WHO vaccination program]. Ann Inst Pasteur Immunol (1985) 1985;136d(2):167-74.
343. Lafarge H, Levy E, Rey M. [Evaluation of vaccination policies. The case of measles]. Rev Epidemiol Sante Publique 1985;33(3):182-93.
344. Gateff C. [Measles throughout the world]. Med Trop (Mars) 1985;45(1):19-25.
345. Fel'dman EV, Kapustik LA, Belous TI, et al. [Effectiveness of the selective revaccination of schoolchildren against measles]. Zh Mikrobiol Epidemiol Immunobiol 1985(6):30-4.
346. Deshevoĭ SE, Rykushin Iu P, Malykhina RM. [Causes of the periodic changes in measles morbidity with vaccine prevention]. Tr Inst Im Pastera 1985;62:34-40.
347. Boutin JP, Debonne JM. [Report on 5 years of expanded program of vaccination in an experimental zone in Ivory Coast]. Med Trop (Mars) 1985;45(3):287-94.
348. Sergiev VP, Dzagurov SG. [Current problems of immunoprophylaxis of infectious diseases and standardization of medical immunobiological preparations]. Zh Mikrobiol Epidemiol Immunobiol 1984(7):29-33.
349. Rodríguez Borrego MJ, Valverde Fernández MA, García Gil MC, et al. [Epidemiological study of measles in Spain from 1949 to 1980, with special reference to Andalusia]. Rev Sanid Hig Publica (Madr) 1984;58(7-8):645-73.
350. Possoli S. [Multivariate analysis technics for the evaluation of health conditions of the municipalities of Rio Grande do Sul, Brazil]. Rev Saude Publica 1984;18(4):288-300. doi: 10.1590/s0034-89101984000400004
351. Janoşi L, Lucasciuc D, Szabo A. [Evolution of measles morbidity during the use of a vaccination program]. Viata Med Rev Inf Prof Stiint Cadrelor Medii Sanit 1984;32(11):241-2.
352. Filipowicz A, Motta E, Guz I, et al. [Humoral immunological reactivity in subacute sclerosing panencephalitis]. Neurol Neurochir Pol 1984;18(4):329-32.
353. Echevarría JM, Sainz C, Montón JL, et al. [Anti-measles antibodies in the first 2 years of life]. An Esp Pediatr 1984;20(9):847-53.
354. Carme B, Guillo du Bodan H, Molez JF, et al. [Retrospective study on the mortality of children under 5 in a rural district of the region of Brazzaville (People's Republic of Congo). I. Rate and causes of mortality]. Bull Soc Pathol Exot Filiales 1984;77(1):104-14.

355. Bolotovskiĭ VM, Gelikman BG, Slavnitskaia IV, et al. [Immunostructure of school-age children inoculated against measles]. Zh Mikrobiol Epidemiol Immunobiol 1984(2):83-7.
356. Bolomovskiĭ VM, Gelikman BG, Titova NS, et al. [Immunological effectiveness of a booster inoculation against measles in children remaining seronegative after vaccination]. Zh Mikrobiol Epidemiol Immunobiol 1984(7):100-6.
357. Zakharova TR, Zargar'iants AI, Titova NS, et al. [Anti-measles immunity in mothers vaccinated with live measles vaccine and in their newborn infants]. Pediatriia 1983(2):29-31.
358. Rey M. [Urbanization and directly transmitted viral infections]. Bull Soc Pathol Exot Filiales 1983;76(3):256-62.
359. Naruszewicz-Lesiuk D. [Measles - 1981]. Przegl Epidemiol 1983;37(1):21-9.
360. Lombardero Rico E. [Measles: morbidity and effect of the La Espina (Salas) vaccine]. Med Clin (Barc) 1983;81(3):95-8.
361. Deshevoĭ SE, Makarova NT, Petrova VN, et al. [The role of vaccinated individuals in measles morbidity during the vaccination period]. Tr Inst Im Pastera 1983;61:158-64.
362. Campello C. [Epidemiology of measles and of its complications]. Ann Sclavo 1983;24(3):260-72.
363. Artemov VG, Zamotin BA, Kondrakhina OK. [Hereditary blood factors and infectious diseases in children in the 1st 7 years of life]. Zh Mikrobiol Epidemiol Immunobiol 1983(4):89-92.
364. [Preliminary report on long-term disease surveillance in Liaoning Province]. Zhonghua Liu Xing Bing Xue Za Zhi 1983;4(3):178-80.
365. Vasil'eva NA, Kashuba VN. [Economic effectiveness of using a live measles vaccine in an agricultural area of the Ukrainian SSR]. Zh Mikrobiol Epidemiol Immunobiol 1982(9):57-60.
366. Rodríguez Véliz AM. [The role of health education in preventive medicine strategy of the Mexican Institute of Social Security]. Hygie 1982;1(3-4):35-42.
367. Pyda E. [Incidence of chronic and recurrent bronchitis and asthma among schoolchildren as related to the degree of atmospheric air pollution and other external factors]. Pneumonol Pol 1982;50(7-8):381-6.
368. Naruszewicz-Lesiuk D. [Measles - 1980]. Przegl Epidemiol 1982;36(1-2):33-40.
369. Măgureanu E, Dobrescu A, Busuioc C, et al. [Evolutive peculiarities of streptococcal infections in the year 1980, in the city of Bucharest]. Rev Ig Bacteriol Virusol Parazitol Epidemiol Pneumoftiziol Bacteriol Virusol Parazitol Epidemiol 1982;27(1):17-22.
370. Gehanno P, Astier P, Fernandez G, et al. [Bacteria seen in acute otitis media in children hospitalised for infectious diseases]. Ann Otolaryngol Chir Cervicofac 1982;99(6):253-5.
371. Charieras JL, Plassart H. [Double-blind study of 59 severe cases of measles treated by isoprinosine (author's transl)]. Med Trop (Mars) 1982;42(3):315-8.
372. Cazacu E, Stefanov I, Roman V, et al. [Characteristics of the epidemiological process of measles in the period of carrying out the vaccination program]. Rev Ig Bacteriol Virusol Parazitol Epidemiol Pneumoftiziol Bacteriol Virusol Parazitol Epidemiol 1982;27(3):167-77.
373. Busse E, Helmholz M. [The influence of L-dopa and of thymus fraction on the survival rate of whole-body-irradiated mice]. Arch Geschwulstforsch 1982;52(4):253-7.
374. Bolotovskiĭ VM, Gelikman BG, Bakhur EA, et al. [Method of detecting persons needing reinoculations against measles]. Zh Mikrobiol Epidemiol Immunobiol 1982(6):19-23.
375. Rodhain F. [World epidemiological situation: viral disease incidence (author's transl)]. Bull Soc Pathol Exot Filiales 1981;74(6):630-5.
376. Omanga U, Mafama N, Valliante K, et al. [Epidemiological and etiologic aspects of children acute gastro-enteritis in Kinshasa (author's transl)]. Med Trop (Mars) 1981;41(6):619-24.
377. Gallais H, Taleb A, Kadio A, et al. [Measles in Abidjan. Apropos of 500 cases]. Bull Soc Pathol Exot Filiales 1981;74(3):283-92.
378. Bergamini F, Breschi F, Ferrante P, et al. [Subacute sclerosing panencephalitis (SSPE); clinical and epidemiological observations of 56 cases studied from 1956 to 1978]. Minerva Med 1981;72(1):21-32.
379. Tkacheva MN, Lobanova EA, Atakian RV, et al. [Measles abroad]. Zh Mikrobiol Epidemiol Immunobiol 1980(11):11-8.
380. Tang DL. [New methods for measurement and comparison of survival rates of neoplasms and immunization results (author's transl)]. Zhonghua Yu Fang Yi Xue Za Zhi 1980;14(3):150-5.
381. Naruszewicz-Lesiuk D. [Measles]. Przegl Epidemiol 1980;34(1):21-7.
382. Gubéran E. [Mortality trends in Switzerland. 2. Infectious diseases 1876-1977]. Schweiz Med Wochenschr 1980;110(15):574-83.
383. Zardi O. [Measles: notes on epidemiology and prevention, including the military community]. Riv Med Aeronaut Spaz 1979;42(1-2):124-60.
384. Wiedermann G. [Modern trends in vaccination policy: evaluation of benefits, risks and cost (author's transl)]. Wien Klin Wochenschr 1979;91(5):143-50.
385. Tsinzerling AV, Kalashnikova EP. [Intrauterine infections in the perinatal period]. Arkh Patol 1979;41(10):49-54.
386. Tournier G, Labrune B, Badoual J. [Pediatrics in 1979]. Rev Prat 1979;29(17):1363-4, 67-8, 71-2 passim.
387. Rykushin Iu P, Malykhina RM. [Reasons for epidemic outbreaks of measles against a background of stable low morbidity]. Tr Inst Im Pastera 1979;53:26-32.
388. Rougemont A, Arnaud MD, Balique H, et al. [An example of the application of factorial analysis of correspondences to infant mortality and its prevention in a rural area of West Africa]. Soz Praventivmed 1979;24(2-3):143-8. doi: 10.1007/bf02094149
389. Plesník V, Vrzáková A, Bindas B, et al. [Investigation of the effectiveness of vaccination against measles in the north Moravian region (author's transl)]. Cesk Epidemiol Mikrobiol Imunol 1979;28(2):65-73.

390. Kibumbe M. [The importance of early consultation during measles in malnourished children]. Ann Soc Belg Med Trop 1979;59(4):427-30.
391. Kalinina LI, Stepanova LG, Rozina EE, et al. [Preparation and characteristics of diploid cell strains from the tissues of a bovine embryo]. Vopr Virusol 1979(2):152-6.
392. Ivanova LM, Gimpelevich SD. [Measles morbidity in the RSFSR in a period of mass immunization]. Tr Inst Im Pastera 1979;53:7-12.
393. Grosser P. [Certain results of infectious disease prevention in the German Democratic Republic]. Zh Mikrobiol Epidemiol Immunobiol 1979(11):62-7.
394. Fara GM. [Epidemiology of measles (author's transl)]. Ann Sclavo 1979;21 Suppl 1:384-93.
395. Eggers HJ. [Evolution of viral diseases (author's transl)]. MMW Munch Med Wochenschr 1979;121(44):1455-60.
396. Donchev D, Ranchov G, Gacheva N. [Dynamic changes in the epidemiological patterns of measles in the People's Republic of Bulgaria after mass vaccination and revaccination]. Vopr Virusol 1979(4):417-22.
397. Bolotovskiĭ VM, Gelikman BG, Auzinia AV, et al. [Effect of active immunization on the periodicity and seasonality of measles morbidity]. Zh Mikrobiol Epidemiol Immunobiol 1979(10):29-33.
398. Ambrosch F. [Epidemiology of measles and mumps. 2nd part: mumps (author's transl)]. Padiatr Padol 1979;14(4):341-5.
399. Ambrosch F. [Epidemiology of measles and mumps. 1st part: measles (author's transl)]. Padiatr Padol 1979;14(4):333-40.
400. Vasil'ev LV. [Reasons for the decrease in viral hepatitis morbidity in Orekhovo-Zueve]. Zh Mikrobiol Epidemiol Immunobiol 1978(3):135-9.
401. Nurakhunov NN. [Measles morbidity against a background of mass vaccinal prophylaxis in Kara-Su District, Osh Province]. Zdravookhr Kirg 1978(1):27-31.
402. Naruszewicz-Lesiuk D. [Measles in Poland]. Przegl Epidemiol 1978;32(1):47-50.
403. Grassi J, Salinas V. [Measles in Paraguay: experience in the Hospital for Infectious and Tropical Diseases of Asunción]. Bol Oficina Sanit Panam 1978;85(3):210-9.
404. Furlan PM, Riccio A, Quattrocolo G, et al. [An atypical case of subacute sclerosing panencephalitis (SSPE), (with special reference to psychiatric and psychological evaluations)]. Minerva Med 1978;69(55):3795-802.
405. Erdös L. [The effect of vaccination against measles on the susceptibility to communicable diseases]. Orv Hetil 1978;119(53):3259-60.
406. Corey G, Rodríguez H. [Measles 1970--1976: various aspects of the last 2 epidemic outbreaks]. Rev Chil Pediatr 1978;49(1-6):229-35.
407. Bächlin A, Berger-Hernandez R, Just M. [Vaccination and morbidity in Basel school-children (measles, mumps, rubella, chickenpox, cytomegalic inclusion disease)]. Soz Praventivmed 1978;23(4):253-6. doi: 10.1007/bf02075140
408. Anańko J, Vieth J, Bochyński K. [Measles encephalitis in children. Report of 15 cases treated at the Infectious Diseases Department in Radom]. Przegl Epidemiol 1978;32(2):253-5.
409. [Reported cases of communicable reportable diseases in Costa Rica, week no. 52, ending December 30, 1978]. Sem Epidemiol 1978;6(52):1-7.
410. Zargar'iants AI, Zetilova LP, Bolotovskii VM, et al. [Necessity of serologic confirmation of the clinical diagnosis of measles under conditions of massive vaccination]. Zh Mikrobiol Epidemiol Immunobiol 1977(6):41-4.
411. Zargar'iants AI, Zemilova LP, Bolotovskií VM, et al. [Epidemiological and immunological study of the foci of measles infection]. Zh Mikrobiol Epidemiol Immunobiol 1977(9):16-20.
412. Strokatova GM, Terekhov SN, Filosofova TG, et al. [Successes in the control of childhood droplet infections in the UkrSSR during the 60 years of Soviet government]. Zh Mikrobiol Epidemiol Immunobiol 1977(3):8-14.
413. Squeri L, Ioli A. [A few viral infections in the recent epidemiological evolution (author's transl)]. Ann Sclavo 1977;19(3):313-46.
414. Sejda J, Losová M. [Vaccination Against measles in the CSR--a six year's balance (author's transl)]. Cesk Epidemiol Mikrobiol Imunol 1977;26(3):139-46.
415. Naruszewicz-Lesiuk D. [Measles]. Przegl Epidemiol 1977;31(1):85-90.
416. Lomberg B, Kotrelova NV. [Epidemiologic analysis of measles morbidity against a background of mass immunization]. Zh Mikrobiol Epidemiol Immunobiol 1977(10):135-6.
417. Kozlova NA, Sobolevskaia AA. [Basic patterns in the epidemic process of measles under conditions of massive immunization]. Zh Mikrobiol Epidemiol Immunobiol 1977(7):62-7.
418. Huber HC. [German measles vaccination]. Fortschr Med 1977;95(33):2007-8.
419. Grzegorek R, Kirbis U, Richter J. [Results of mass-longitudinal screening in Görlitz. 1. Morbidity of important infectious diseases]. Arztl Jugendkd 1977;68(4):233-7.
420. Colette C, Assus M, Attal B, et al. [Non-specific fetal infections: possibilities and value of early diagnosis]. J Gynecol Obstet Biol Reprod (Paris) 1977;6(5):673-82.
421. Calderón E, Martin Sosa S, Milovanovic MV, et al. [Immunity against measles in mother-child binomials]. Bol Med Hosp Infant Mex 1977;34(1):1-12.
422. Borgoño JM, Infante A, Vicent P, et al. [The effect of nation wide vaccination programs upon the morbidity and mortality of some infectious diseases (author's transl)]. Rev Med Chil 1977;105(5):335-40.
423. Askerov VF, Piriev GG. [Clinico-immunological reactions to live measles vaccine of 6-16 strain in children suffering from exudative diathesis and bronchial asthma]. Pediatriia 1977(1):32-3.
424. Van Poperinghe M, Audebert M, Grumbach Y, et al. [Spontaneous subcutaneous mediastinal emphysema in children]. Ann Pediatr (Paris) 1976;23(3):191-7.

425. Rossipal E, Dutz W, Kohout E, et al. [Considerations regarding the influence of intrauterine and early postnatal diseases and nutritional deficiencies on immunity and disease epidemiology (author's transl)]. Immun Infekt 1976;4(5):229-35.
426. Naruszewicz-Lesiuk D. [Measles]. Przegl Epidemiol 1976;30(1):101-3.
427. Mitschke H. [Virological investigations in peripheral idiopathic facial pareses (author's transl)]. Wien Klin Wochenschr 1976;88(20):664-7.
428. Marti Cartaya JL, Alvarez Calero A, Martin Fernández D, et al. [Epidemiology of congenital heart diseases in the island of Tenerife]. Rev Esp Cardiol 1976;29(6):545-52.
429. Huber EG, Rannon L, Galffy G. [Long-term-follow-up-study after measles-vaccination (author's transl)]. Padiatr Padol 1976;11(1):72-6.
430. Hennessen W. [Developments in vaccines for prophylactic use (author's transl)]. Zentralbl Bakteriol Orig B 1976;163(1-4):55-62.
431. Gavrilă I, Soloviev M, Cîrstina D, et al. [Current aspects of infectious mononucleosis]. Rev Ig Bacteriol Virusol Parazitol Epidemiol Pneumoftiziol Bacteriol Virusol Parazitol Epidemiol 1976;21(4):207-12.
432. Roĭtman MP. [Methods of measuring economic effectiveness in public health and some results of their use in the USSR]. Sov Zdravookhr 1975(6):8-13.
433. Naruszewicz-Lesiuk D. [Measles]. Przegl Epidemiol 1975;29(1):103-5.
434. Joppich G. [Changes in pediatrics (author's transl)]. Monatsschr Kinderheilkd (1902) 1975;123(6):513-7.
435. Cendrowski W. [Cellular immunity in subacute sclerosing panencephalitis and multiple sclerosis]. Neurol Neurochir Pol 1975;9(1):119-25.
436. Kostrzewski JM, Michalowska A. [Clinical picture of measles complications]. Przegl Epidemiol 1974;28(3):309-13.
437. Allwood Paredes J. [The impact of measles in Central America]. Bol Oficina Sanit Panam 1974;76(6):503-11.
438. Topciu V, Moldovan E, Plavoşin L, et al. [Use of serological examinations in the epidemiological study of measles]. Stud Cercet Virusol 1973;24(6):453-9.
439. Terekhov SN. [Achievements in the control of measles in the UkrSSR]. Zh Mikrobiol Epidemiol Immunobiol 1973;50(1):10-3.
440. Stoian C, Gulea E, Doicescu I, et al. [Antidiphtheria immunity levels following various infectious diseases]. Arch Roum Pathol Exp Microbiol 1973;32(2):219-25.
441. Popescu-Pretor I, Răducanu S, Anghel A, et al. [Morbidity of measles in vaccinated subjects in the year 1969 in the district of Constanta]. Stud Cercet Virusol 1973;24(4):305-10.
442. Moulias R, Goust JM, Reinert P, et al. [Transfer factor of cellular immunity. Preliminary therapeutic trials during specific immunologic deficiencies of an antigen in human clinical practice]. Nouv Presse Med 1973;2(20):1341-4.
443. Moulias R, Goust JM, Reinert P, et al. [Transfer factor of cellular immunity. Preliminary therapeutic trials during specific immunologic deficiencies of an antigen in human clinical practice]. Nouv Presse Med 1973;2(20):1341-4.
444. Măgureanu E, Feller H, Cristea A, et al. [Use of mathematical models in the interpretation of data concerning the evolution of measles morbidity in Rumania]. Rev Roum Virol (1972) 1973;10(4):301-17.
445. Măgureanu E, Feller H. [Data on the epidemiology of measles in Rumania]. Stud Cercet Virusol 1973;24(4):261-79.
446. Hlouskovà Z, Nosàlovà J. [Respiratory diseases and their development in children after measles pneumonia]. Cesk Pediatr 1973;28(4):158-60.
447. Chudnaia LM, Shekhter AB, Fastovskaia BN, et al. [Epidemiological and serological study of the causes of measles morbidity in children inoculated against this infection]. Zh Mikrobiol Epidemiol Immunobiol 1973;50(9):7-10.
448. Cajal N, Cepleanu M, Sorodoc Y, et al. [Current status of specific prevention of measles]. Stud Cercet Virusol 1973;24(4):249-59.
449. Buşilă VT, Vasilescu I, Fitărău A, et al. [Sero-immunological studies of measles in infants]. Stud Cercet Virusol 1973;24(4):281-4.
450. Tarasov VI, Gladkikh VK. [Measles morbidity among adults]. Voen Med Zh 1972;7:58-9.
451. Naruszewicz-Lesiuk D. [Measles as a problem of social medicine in Poland]. Przegl Epidemiol 1972;26(2):165-72.
452. Heidel G, Olbricht H, Ely K, et al. [Influence of measles vaccination using the live measles virus vaccine SSW-L 16 on the complication rate in measles]. Z Arztl Fortbild (Jena) 1972;66(3):171-4.
453. Enders G. [Measles vaccination]. Zentralbl Bakteriol Orig A 1972;220(1):273-83.
454. Berthaux P, Moulias R, Goust JM, et al. [Specific immunotherapy using cell-mediated immune transfer factor. Preliminary results with semi-purified transfer factor]. Ann Med Interne (Paris) 1972;123(12):1069-72.
455. Tisseuil J. [Is the notion of premunition an obsolete one?]. Bull Soc Pathol Exot Filiales 1971;64(4):429-33.
456. Siegert R. [Indications for possibilities of preventive vaccination against measles and rubella]. Internist (Berl) 1971;12(8):309-12.
457. Schmidt H, Köhler FC. [Analysis of morbidity in children's day nurseries]. Z Arztl Fortbild (Jena) 1971;65(4):224-8.
458. Provost A, Maurice Y, Borredon C. [Rinderpest protection of bovines by measles virus. II. Vaccination of calves born from cows vaccinated with the MB 113 strain]. Rev Elev Med Vet Pays Trop 1971;24(2):167-72.
459. Havlík J, Kottová A. [Course of measles in a contemporary epidemic in the area of the infectious disease clinic of the Bulovka-hospital in Prague]. Cesk Pediatr 1971;26(7):361.
460. Dietzsch HJ, Leupold W, Rogner G. [Occurrence and importance of complications in measles]. Z Arztl Fortbild (Jena) 1971;65(3):138-40.
461. Borgoño JM, Greiber R. [Measles. 5 years' experience with the vaccination program in Chile]. Rev Med Chil 1971;99(7):500-6.

462. Strauss J. [Infestation of the population with the rubeola virus in selected areas of Czechoslovakia]. Cas Lek Cesk 1970;109(49):1133-6.
463. Shakhanina IL, Argutina TP, Sumarokov AA. [The social-economic significance of infectious diseases. I. Characteristics of the role of infectious morbidity during temporary disability]. Br J Soc Clin Psychol 1970;9(4):61-7.
464. Schopp W. [Tropical medicine and ubiquitous diseases]. Munch Med Wochenschr 1970;112(43):1929-32.
465. Popov VF, Rybkina NM. [The epidemiologic patterns of measles in the USSR]. Br J Soc Clin Psychol 1970;9(4):68-73.
466. Pène P, Bourgeade A, Serres JJ, et al. [Mediastinal emphysema, a frequent complication of measles in tropical environment. 46 cases]. Sem Hop 1970;46(15):989-99.
467. Naruszewicz-Lesiuk D. [Measles in Poland in the years 1962-1968 as viewed against the background of the epidemiologic situation throughout the world]. Przegl Epidemiol 1970;24(1):1-14.
468. Maslovskaia G. [Infectious morbidity in the USA (literature review)]. Zh Mikrobiol Epidemiol Immunobiol 1970;47(11):126-34.
469. Lebart L, Péquignot H, Rosch G. [Public health policy. Smallpox vaccination in the USA, and measles vaccination in Senegal]. Sem Hop 1970;46(42):2672-7.
470. Laugier J, Grenier B, Mercier C, et al. [3 cases of nontraumatic pneumomediastinum in young infants and children]. Ann Pediatr (Paris) 1970;17(1):37-48.
471. Krupińska-Sanecka I. [Measles in tropical Africa]. Pediatr Pol 1970;45(11):1363-6.
472. Jellinger K, Radl H. [Unusual complication following measles encephalitis]. Wien Klin Wochenschr 1970;82(3):42-50.
473. Ehrengut W, Kohlmann HG. [Serological immunity against German measles in Hamburg. Preliminary experiences with German measles vaccination with the Cendehill strain]. Dtsch Med Wochenschr 1970;95(10):501-5. doi: 10.1055/s-0028-1108492
474. Clément R. [Immunization against infectious diseases]. Presse Med (1893) 1970;78(7):315-7.
475. Budai J, Tóth M, Melis L. [Experimental active and passive immunization against rubeola]. Orv Hetil 1970;111(47):2767-72.
476. Wedler E. [Frequency of allergens causing bronchial asthma in childhood with reference to sex and age]. Allerg Asthma (Leipz) 1969;15(6):330-6.
477. Spiess H, Stehr K. [Indication and use of gamma globulin]. Dtsch Med Wochenschr 1969;94(6):282-5. doi: 10.1055/s-0028-1108944
478. Shtarke G, Glinak P, Graneĭs K. [An epidemiologic analysis of the effectiveness of immunization against measles thoughout the year following the vaccination campaign]. Zh Mikrobiol Epidemiol Immunobiol 1969;46(9):46-9.
479. Saito H. [Study of immunoglobulin levels and antibody formation in allergic children]. Arerugi 1969;18(4):263-76.
480. Karachunskaia MI. [Infectious disease morbidity and the most important causes of death in England (review of the foreign literature)]. Zh Mikrobiol Epidemiol Immunobiol 1969;46(9):91-6.
481. Hahn M. [Various acute infectious diseases in day nursery. I. Measles, chickenpox]. Z Arztl Fortbild (Jena) 1969;63(1):41-4.
482. Charpin J, Boutin C, Aubert J, et al. [Statement about a prospective study on child asthma]. Ann Pediatr (Paris) 1969;16(5):326-33.
483. Vanista J, Adamová V, Mílek E, et al. [Purpura in German measles in 6 children]. Cesk Pediatr 1968;23(6):509-12.
484. Turczynowski R, Kisielewicz J. [Causes of deaths in children up to 4-years-old in the town of Chojna in the 2d half of the 18th century]. Ann Acad Med Stetin 1968;14:335-43.
485. Sukhareva ME, Berzina LA, Shapiro SL, et al. [Dynamics of epidemiological and clinical characteristics of measles]. Pediatriia 1968;7:30-4.
486. Sejda J, Hrachovina V, Vítková V, et al. [Frequency of complications in measles]. Cesk Pediatr 1968;23(4):293-300.
487. Procházka J, Mílek E, Grantová H. [Importance of measles in Czechoslovakia]. Cesk Pediatr 1968;23(4):289-92.
488. Markvart K, Novák K, Sejda J, et al. [Analysis of reported morbidity in measles in the Northern and Eastern region of Bohemia]. Cesk Epidemiol Mikrobiol Imunol 1968;17(5):280-7.
489. Gsell O. [Epidemiology of infectious diseases since the application of antibiotics and chemotherapeutic agents. Statistically demonstrable change in mortality, lethality and morbidity of infectious diseases within the last 40 years]. Antibiot Chemother 1968;14:1-51.
490. Yoneda Y. [Clinical and immunoserological study of vaccine and spontaneous measles]. Nihon Shonika Gakkai Zasshi 1967;71(11):1406-16.
491. Voltay B. [How long can a child with measles be treated at home?]. Orv Hetil 1967;108(20):942-4.
492. Veronesi R, Penna HA, Issler H, et al. [Measles and measles vaccination in Brazil. Magnitude of the problem and contribution to the method of small doses in mass immunization. Clinico-serological evaluation of 2 types of highly attenuated vaccine]. Hospital (Rio J) 1967;72(4):1203-32.
493. Ulivelli A, Casini Raggi G, Silenzi M. [Pneumopathies during measles: considerations on 314 cases of bronchopulmonary complications]. G Mal Infett Parassit 1967;19(4):197-203.
494. Spiess H. [Immunologic prevention of measles]. Monatsschr Kinderheilkd (1902) 1967;115(9):490-1.
495. Shatrov, II, Mastiukova Iu N, Ignat'eva GV, et al. [Achievements in the control of measles mortality in the RSFSR during the years of Soviet power]. Zh Mikrobiol Epidemiol Immunobiol 1967;44(11):60-6.
496. Payet M, Pène P, Sankale M, et al. [Public health in the Niayes region (Senegal)]. Sem Hop 1967;43(28):1815-28.
497. Le Viguelloux J, Millan J. [Reflections apropos of measles in Africa]. Med Trop (Mars) 1967;27(3):Suppl:1-10.
498. Hässig A. [On the prophylaxis and therapy with human immune globulin preparations]. Bibl Haematol 1967;27:239-52.
499. Grantová H, Mílek E. [Infectious diseases and pregnancy]. Dtsch Med J 1967;18(16):489-96.
500. Ehrengut W. [Current aspects of new immunizations]. Landarzt 1967;43(36):1777-83.
501. Biener K. [Accident and surgery anamnesis indolescence]. Praxis 1967;56(43):1460-4.

502. Adam E, Mares I, Drevo M, et al. [Study on the dynamics of the decline of transplacentary transferred antibodies against measles in infants]. Cesk Pediatr 1967;22(12):1075-9.
503. Wiederhold A. [(Measles and its complications). Clinical Study]. Rev Chil Pediatr 1966;37(8):470-5.
504. Suárez Guglielmini H, Schürmann Ruppert R, O'Ryan Costa P. [Measles. Clinical and anatomo-pathologic study]. Rev Chil Pediatr 1966;37(8):477-84.
505. Polster H, Grimm J. [Prophylaxis and therapy with human gamma-globulin in childhood]. Z Arztl Fortbild (Jena) 1966;60(18):1029-33.
506. Feo F, Angela GC. [Measles today]. Minerva Med 1966;57(22):954-73.
507. [Mortality statistics (1965). (Typhoid, diphtheria, tetanus, measles, whooping cough infections)]. Bull Inst Natl Sante Rech Med 1966;21(4):689-708.
508. Starke G. [Experiences with the live measles virus vaccines L-4 and L-16 in the German Democratic Republic]. Pathol Microbiol (Basel) 1965;28(6):950-5.
509. Revenok ND, Smorodintsev AA. [Results of the use of the Leningrad-4 live measles vaccine during increasing measles morbidity]. Tr Leningr Nauchnoissled Inst Epidemiol Mikrobiol 1965;28:361-71.
510. Măgureanu E, Feller H. [Contributions to the study of the historical evolution of some infectious diseases in Rumania. II. Measles (1898-1964)]. Arch Roum Pathol Exp Microbiol 1965;24(4):959-78.
511. Decker J. [On measles complications in Africa]. Z Tropenmed Parasitol 1965;16(4):437-44.
512. Cantrelle P. [MEASLES MORTALITY AND MORBIDITY IN FRENCH-SPEAKING COUNTRIES OF WEST AFRICA (SURVEY METHODS AND RESULTS]. Arch Gesamte Virusforsch 1965;16:35-45.
513. [Mortality statistics (1964). Typhoid, diphtheria, tetanus, measles, whooping cough infections]. Bull Inst Natl Sante Rech Med 1965;20(5):625-44.
514. Uher M, Uhlir M, Bednar A. [PERINATAL MORTALITY IN MATERNAL INFECTIONS]. Cesk Gynekol 1964;29:562-7.
515. Solodovnikov IP. [MEASLES IN DIFFERENT COUNTRIES OF THE WORLD AND ACTIVE IMMUNIZATION AGAINST THE DISEASE. (REVIEW OF THE LITERATURE)]. Pediatriia 1964;43:80-4.
516. Smorodintsev AA, Boichuk LM, Shikina ES. [LIVE VACCINE AGAINST MEASLES]. Vopr Okhr Materin Det 1964;9:3-7.
517. Perelman R. [THE COMPLICATIONS OF WHOOPING COUGH]. Concours Med 1964;86:1333-40.
518. Dudas P, Augustin V. [ROLE OF VARIOUS ORGANISMS IN LUNG DISEASES COMPLICATING MEASLES]. Gyermekgyogyaszat 1964;15:80-5.
519. Bastin R, Verliac F, Frottier J. [MEASLES IN HOSPITALS IS TODAY A BENIGN INFECTION. (INFORMATION DERIVED FROM A RECENT STATISTICAL SERIES OF THE H OPITAL CLAUDE-BERNARD)]. Presse Med (1893) 1964;72:1711-5.
520. Veronesi R, Schmid AW, Mourarde A, et al. [REVIEW OF DATA CONCERNING THE EPIDEMIOLOGY AND ETIOLOGY OF MEASLES AND AIDS FOR VACCINATION AGAINST THE DISEASE]. Arq Fac Hig Saude Publica Univ Sao Paulo 1963;17:135-204.
521. Ristori C, Boccardo H, Miranda M, et al. [VACCINATION AGAINST MEASLES WITH LIVE VIRUS FROM THE EDMONSTON STRAIN-NATIONAL EXPERIENCE]. Rev Chil Pediatr 1963;34:656-63.
522. Leverington J, Soriano H, Perez M. [MEASLES IN THE AREA OF VI NA DEL MAR AS A PROBLEM OF HEALTH]. Rev Chil Pediatr 1963;34:868-74.
523. Kassai S, Molnar M. [NEROBOL OVERDOSAGE IN INFANCY AND CHILDHOOD]. Gyermekgyogyaszat 1963;14:370-4.
524. Sakamoto K. [A maximum likelihood method in medicine]. Osaka City Med J 1962;11:361-5.
525. Monastiri H. [Some statistical data relating to mortality from measles in the commune of Tunis]. Tunis Med 1961;39:179-87.
526. Hoogendoorn D. [Measles mortality in the Netherlands]. Ned Tijdschr Geneeskd 1957;101(23):1074-7.
527. Mathov E, Schcolnicov J. [Asthma in Buenos Aires; statistical examination of 35,000 asthmatic attacks; relation of asthmatic attack frequency to influenza, measles, biliary colic and epileptic seizures]. Sem Med 1954;105(18):799-803; passim.
