# Supplementary material for: Measles vaccines and non-specific effects on mortality or morbidity: A systematic review and meta-analysis
Source: PLoS One. 2025 Jul 2;20(7):e0321982. doi: 10.1371/journal.pone.0321982 (PMC12221017; doi:10.1371/journal.pone.0321982)
Supplement: S1 Table — (DOCX) [file pone.0321982.s004.docx]

**S1 Table. Systematic search string**

|  | Intervention column 1 |  | Outcome column 2A |
| --- | --- | --- | --- |
| MeSH-terms/ keywords | Measles vaccine or Measles prevention and control or Vaccines, attenuated or | And | Cross protection or Immunity, heterologous or Immunity, maternally-acquired or |
| Text Word | ”Live attenuated measles vaccine” or  ”Live attenuated” or  “Measles vaccine” or  “MMR vaccine” or  “MMRV vaccine” or “MMR” or “MFR” or “MMRV” or “Measles containing” or   “measles-containing” or “measles-containing-vaccine” or “Measles containing vaccine”  AND  “Measles” AND Vaccine* |  | “Non-specific effect” or “Nonspecific effect” or “Nonspecific immunity” or Non specific immunity” or “Cross protection” or “Heterologous immunity” or “Maternally acquired immunity” or “ Indirect effect” or “Non-targeted infection” or “In-direct effect” or “Indirect effect” or Indirect immunity” or “In-direct immunity” or “Trained immunity” or “Long-term beneficial effect” or “Non-targeted immunization” or “Off-target immunization” or “Survival” or “Morbidity” or “Atopy” or “Mortality” or “Admission” or “Hospital contact” or “Hospitalization” or “Hospitalisation” or “asthma” or “atopic dermatitis” AND “measles” |

Pubmed:

((((((((((((((((((("Measles Vaccine"[Mesh]) OR "Vaccines, Attenuated"[Mesh]) OR "Measles/prevention and control"[Mesh]) OR ("Live attenuated measles vaccine"[Text Word])) OR ("Live attenuated"[Text Word])) OR ("Measles vaccine*"[Text Word])) OR ("MMR vaccine*"[Text Word])) OR ("MMRV vaccine*"[Text Word])) OR (MMR*[Text Word])) OR (MFR*[Text Word])) OR (MMRV*[Text Word])) OR ("measles containing*"[Text Word])) OR ("measles-containing*"[Text Word])) OR ("measles-containing-vaccine"[Text Word])) OR ("Measles containing vaccine"[Text Word])) OR (measles*[Text Word])) OR (vaccine*[Text Word])) OR (MCV*[Text Word])) AND ((((((((((((((((((((((((((("Cross Protection"[Mesh]) OR "Immunity, Heterologous"[Mesh]) OR "Immunity, Maternally-Acquired"[Mesh]) OR (Non-specific effect*[Text Word])) OR (Nonspecific effect*[Text Word])) OR (Nonspecific immunity*[Text Word])) OR (Non specific immunity*[Text Word])) OR (Cross protection*[Text Word])) OR (Heterologous immunity*[Text Word])) OR (Maternally acquired immunity*[Text Word])) OR (Indirect effect*[Text Word])) OR (Non-targeted infection*[Text Word])) OR (In-direct effect*[Text Word])) OR (Indirect effect*[Text Word])) OR (Indirect immunity*[Text Word])) OR (In-direct immunity[Text Word])) OR (Trained immunity*[Text Word])) OR (Long-term beneficial effect*[Text Word])) OR (Non-targeted immunization*[Text Word])) OR (Off-target immunization*[Text Word])) OR (Survival*[Text Word])) OR (Morbidity*[Text Word])) OR (Atopy*[Text Word]))OR (Mortality*[Text Word])) OR (Admission*[Text Word])) OR (Hospital contact*[Text Word])) OR (Hospitalization*[Text Word])) OR (Hospitalisation*[Text Word])) OR (asthma*[Text Word]) OR (atopic dermatitis*[Text Word]) AND (measles[Text Word]) 

Embase:

1: exp measles mumps vaccine/ or exp measles rubella vaccine/ or exp measles vaccination/ or exp measles vaccine/

2: ((Live attenuated measles vaccine or Live attenuated or Measles vaccine* or MMR vaccine* or MMRV vaccine or MMR* or MFR vaccine or MMRV* or MFR* or MCV* or Measles containing or Measles containing vaccine*) and measles and vaccine*).mp. [mp=title, abstract, heading word, drug trade name, original title, device manufacturer, drug manufacturer, device trade name, keyword heading word, floating subheading word, candidate term word]

3: 1 or 2

4: exp heterologous immunity/ or exp immunomodulation/ or exp cross protection/

5: (Survival* or Morbidity* or Mortality* or Admission* or hospital contact* or Hospitali?ation* or atopy or asthma or atopic dermatitis).ab,kw,ti.

6: (In-direct effect* or Indirect effect* or Indirect immunity or In-direct immunity or Trained immunity* or Long-term beneficial effect* or Non-targeted immunization* or Cross protection* or Heterologous immunity* or Maternally acquired immunity*).mp. [mp=title, abstract, heading word, drug trade name, original title, device manufacturer, drug manufacturer, device trade name, keyword heading word, floating subheading word, candidate term word]

7: 4 or 5 or 6

8: 3 and 7

9: limit 8 to conference abstracts

10: 8 not 9

11: limit 10 to (meta analysis or "systematic review")

12: 10 not 11

13: 12 not ((exp animal/ or exp invertebrate/ or nonhuman/ or animal experiment/ or animal tissue/ or animal model/ or exp plant/ or exp fungus/) not (exp human/ or human tissue/))

14: limit 13 to (danish or english or norwegian or swedish)

S1 Table: Systematic search string and raw text than can be used to reproduce the same search on Pubmed and Embase.
