# Supplementary material for: Measles vaccines and non-specific effects on mortality or morbidity: A systematic review and meta-analysis
Source: PLoS One. 2025 Jul 2;20(7):e0321982. doi: 10.1371/journal.pone.0321982 (PMC12221017; doi:10.1371/journal.pone.0321982)
Supplement: S2 Table — (DOCX) [file pone.0321982.s005.docx]

**S2 Table. Risk of bias assessment.**

| Year | Author | Randomisation process | Deviations from intended interventions | Blinding of participants and personnel | Measurement of the outcome | Missing outcome data | Selection of the reported results | Other source of bias | Overall quality grading |
| --- | --- | --- | --- | --- | --- | --- | --- | --- | --- |
| 2010 | Aaby et al |  |  |  |  |  |  | a) |  |
| 2014 | Martins et al |  |  |  |  |  |  | c) |  |
| 2018 | Fisker et al |  |  |  |  |  |  | a) |  |
| 2017 | Do et al |  |  |  |  |  |  | c) |  |
| 2007 | Aaby et al |  |  |  |  |  |  | c) |  |
| 2014 | Aaby et al |  |  |  |  |  |  | c) |  |
| 2018 | Schoeps et al |  |  |  |  |  |  | c) |  |
| 2018 | Brønd et al |  |  |  |  |  |  | c) |  |
| 2020 | Varma et al |  |  |  |  |  |  | a) |  |
| 2016 | Rasmussen et al |  |  |  |  |  |  | c) |  |
| 2020 | Steiniche et al |  |  |  |  |  |  | c) |  |
| 2007 | Hennino et al |  |  |  |  |  |  | b) |  |
| 1994 | Aaby et al |  |  |  |  |  |  | a) |  |
| 1993 | Aaby et al |  |  |  |  |  |  | b) |  |
| 2021 | Byberg et al |  |  |  |  |  |  | a) |  |
| 2022 | Berendsen et al |  |  |  |  |  |  | a) |  |
| 1996 | Aaby et al |  |  |  |  |  |  | c) |  |
| 1994 | Aaby et al |  |  |  |  |  |  | c) |  |
| 2002 | Libmann et al |  |  |  |  |  |  | a) |  |
| 1991 | Garenne et al |  |  |  |  |  |  | a) |  |
| 1993 | Holt et al |  |  |  |  |  |  | c) |  |
| 2022 | Nielsen et al |  |  |  |  |  |  | a) |  |
| 2023 | Zimakoff et al |  |  |  |  |  |  |  |  |
| Other source of bias: a) external validity, b) small population, c) secondary analyses | | | | | | | | |  |

S2 table: While the format of this table is different from the Rob-2 template, the content is the same and created independently by three authors. All discrepancies were discussed, and consensus was made. Green: Low risk of bias. Yellow: Moderate risk of bias. Red: High risk of bias
