# Supplementary material for: Measles vaccines and non-specific effects on mortality or morbidity: A systematic review and meta-analysis
Source: PLoS One. 2025 Jul 2;20(7):e0321982. doi: 10.1371/journal.pone.0321982 (PMC12221017; doi:10.1371/journal.pone.0321982)
Supplement: S3 Table — (DOCX) [file pone.0321982.s006.docx]

## **S3 Table. Study characteristics and data included in main meta-analyses.**

| Mortality |  |  |  |  |  |  |
| --- | --- | --- | --- | --- | --- | --- |
| Trial | Original trial? | Inclusion in main meta-analysis | Sample size | Intervention | Risk of bias | Follow up |
| P. Aaby et al, 2010 | Yes | Yes, forest plot and TSA | 6648 | Two dose vs one dose (early dose) | High due to lack of blinding | 4.5-36 months |
| P. Aaby et al, 2014 | No | No | 1765 | Two dose vs one dose (early dose) | High due to lack of blinding | 4.5-9 months |
| A. Fisker et al, 2018 | Yes | Yes, forest plot and TSA | 8454 | Two dose vs one dose (early dose) | High due to lack of blinding | 4-36 months |
| A. Schoeps et al, 2018 | No | No | 4559 | Two dose vs one dose (early dose) | High due to lack of blinding | 4.5-36 months |
| S. Nielsen et al, 2022 | Yes | Yes, forest plot and TSA | 6636 | Two dose vs one dose (early dose) | High due to lack of blinding | 4-60 months |
| M. Berendsen et al, 2022 | Yes | Yes, forest plot | 3164 | Two dose vs one dose (booster dose) | High due to lack of blinding | 18-48 months |
| S. Byberg et al, 2021 | Yes | Yes, forest plot | 4767 | MCV for all vs restrictive policy | High due to lack of blinding | 9-60 months |
| Morbidity | | | | | |  |
| Trial | Original trial? | Inclusion in main meta-analysis | Sample size | Intervention | Risk of bias | Follow-up |
| A. Varma et al, 2020 | Yes | Yes, forest plot | 8996 | MCV to intervention group irrespective of prior vaccines | High due to lack of blinding | 2 months from intervention |
| M. Berendsen et al, 2022 | Yes | Yes, forest plot | 3164 | Two dose vs one dose (booster dose) | High due to lack of blinding | 18-48 months |
| M. Brønd et al, 2018 | No | Yes, forest plot and TSA | 5626 | Two dose vs one dose (early dose) | High due to lack of blinding | 4.5-18 months |
| Martins et al, 2014 | No | No | 6648 | Two dose vs one dose (early dose) | High due to lack of blinding | 4.5-9 months |
| A. Schoeps et al, 2018 | No | Yes, forest plot and TSA | 4559 | Two dose vs one dose (early dose) | High due to lack of blinding | 4.5-36 months |
| M. Steiniche et al, 2020 | No | No | 3750 | Two dose vs one dose (early dose) | High due to lack of blinding | 4-9 months |
| V. Do et al, 2017 | No | No | 1625 | Two dose vs one dose (early dose) | High due to lack of blinding | 4.5-9 months |
| S. Byberg et al, 2021 | Yes | Yes, forest plot | 4767 | MCV for all vs restrictive policy | High due to lack of blinding | 9-60 months |
| A. Zimakoff et al, 2023 | Yes | Yes, forest plot | 6540 | Early dose vs placebo | Low | 5-12 months |
| Atopy | | | | | |  |
| Trial | Original trial? | Inclusion in main meta-analysis | Sample size | Intervention | Risk of bias | Follow-up |
| A. Hennino et al, 2007 | Yes | No | 12 | MV vs placebo | Low | 6 months |
| Growth | | | | | |  |
| Trial | Original trial? | Inclusion in main meta-analysis | Sample size | Intervention | Risk of bias | Follow-up |
| S. Rasmussen et al, 2016 | No | No | 6417 | Two dose vs one dose (early dose) | High due to lack of blinding | 4.5-24 months |

S3 Table. Trials presented according to outcome. Table is colour coded: All trials that are presented in the same colour share the same study population and therefore only one trial from each colour could be included in the same meta-analysis.
