# Supplementary material for: Measles vaccines and non-specific effects on mortality or morbidity: A systematic review and meta-analysis
Source: PLoS One. 2025 Jul 2;20(7):e0321982. doi: 10.1371/journal.pone.0321982 (PMC12221017; doi:10.1371/journal.pone.0321982)
Supplement: S4 Table — (DOCX) [file pone.0321982.s007.docx]

## **S4 Table. Full data extraction including estimates and 95% confidence intervals.**

| Author and year | Design | Follow-up | Population | Vaccine | Data extraction and eligibility | Main findings | Secondary analyses | Conflict of interest |
| --- | --- | --- | --- | --- | --- | --- | --- | --- |
|  |  |  |  |  |  |  |  |  |
| 1: P. Aaby et al, 2010 | Original RCT, Bandim. No placebo, not fully  blinded | 4.5-36 months | 6648 children 4.5 months  6417 included in main analysis | **Group A:** Edmonston-Zagreb measles vaccine at 4.5 and 9 months of age.  **Group B:** No vaccine at 4.5 months and Edmonston-Zagreb measles vaccine at 9 months of age.  **Group C:** No vaccine at 4.5 months and Schwarz measles vaccine at 9 months of age. | Data extraction by LF and AC in August 2022.  Eligible for inclusion: Yes  Eligible for analysis: Yes. | **Mortality,** **9-36 months, MRR**  **ITT**: 0. 78 (0.59-1.05)  **Censoring measles:** 0.84 (0.63 - 1.12) SEM 0.1468.  *Crude data*  Event intervention: 65  Total intervention: 2129  Event control: 165  Total control: 4288  *Crude data, male*  Event intervention: 36  Total intervention: 1084  Event control: 75  Total control: 2151  *Crude data, female*  Event intervention: 29  Total intervention: 1045  Event control: 90  Total control: 2137 | **Mortality, 4.5-36 months, MRR**  **ITT_female_**: 0.64 (0.42 - 0.98)  **ITT_male_**: 0.95 (0.64 - 1.42)  **Sex interaction:** 0.68 (0.38-1.20)  **1 vs 0 dose**: MRR from 4.5-9 months 0.67 (0.38-1.19) | None |
| 2: P. Aaby et al, 2014 | Data from #1 and another RCT 1993-1995. No placebo, not fully blinded. Incomplete outcome data. 14/15 deaths occurred after second vaccination | 6-60 months | Population 1: 1765 children Population 2: 750 children 4.5 months | Refer to #1 | Data extraction by LF and AC in August 2022.  Eligible for inclusion: Yes  Eligible for analysis: No | **Mortality, MRR**  **MCV vs no MCV:** 0.61 (0.34–1.09)  With MV:  Antibodies present vs no antibodies present  0.29 (0.09–0.91) |  | None |
| 3: Martins et al, 2014 | Data from #1. No placebo, not fully blinded | 4.5-36 months | Refer to #1 | Refer to #1 | Data extraction by LF and AC in August 2022.  Eligible for inclusion: Yes  Eligible for analysis: No | **Admissions, 4.5-9 months, 1 vs 0 dose: HRR**  **Intervention vs control:** 0.70 (0.52–0.95)**Excluding measles admissions:** 0.78 (0.58–1.07) | **Female**: 0.53 (0.32–0.86)  **Excluding measles admissions, female**: 0.59 (0.36–0.97)  **Male:** 0.86 (0.58–1.26)  **Excluding measles admissions, male**: 0.96 (0.64–1.41) | None |
| 4: M Brønd et al, 2018 | Data from #1. No placebo, not fully blinded | 4.5-18 months | Refer to #1 | Refer to #1 | Data extraction by LF and AC in August 2022.  Eligible for inclusion: Yes  Eligible for analysis: Yes. | **Admissions, excluding measles, HR**  **Intervention vs control:** 0.86 (0.68–1.10), SEM 0.1237  *Crude data*  Event intervention: 97  Total intervention: 1952  Event control: 214  Total control: 3645  *Crude data, male*  Event intervention: 53  Total intervention: 994  Event control: 113  Total control: 1815  *Crude data, female*  Event intervention: 44  Total intervention: 958  Event control: 101  Total control: 1830 |  | None |
| 5: S. Rasmussen et al, 2016 | Data from #1. No placebo, not fully blinded | 4.5-24 months | Refer to #1 | Refer to #1 | Data extraction by LF and AC in August 2022.  Eligible for inclusion: Yes  Eligible for analysis: No | **Growth, 24 months, Mid-upper-arm-circumference (MUAC/in cm)**  **Intervention vs control:** 0.08 (0.02-0.14)  **9 months:** no effect  **Weight for length:** no effect  Half of control group received extra MV at 18 months | Female**:** 0.12 (0.03-0.20).  Male: 0.05 (−0.04- 0.13) | None |
| 6: A. Fisker et al, 2018 | Original two-center RCT. No placebo, not fully blinded | 4-36 months | 8309 children, 121-215 days (3750 in Guinea-Bissau, 4559 in Burkina Faso) | Extra early standard dose of MCV (EZ strain) or no extra MCV 4 weeks after DTP3. All children received routine MCV at 9 months. | Data extraction by LF and AC in August 2022.  Eligible for inclusion: Yes  Eligible for analysis: Yes. | **Mortality, 4-36 months, HR**  **PPA:** 1.05 (0.75–1.46) SEM 0.1699  *Crude data*  Event intervention: 74  Total intervention: 4153  Event control: 71  Total control: 4156  ITT: 1.12 (0.87-1.44)  *Crude data*  Event intervention: 129  Total intervention: 6999  Event control: 114  Total control: 6960  *Crude data, male*  Event intervention: 67  Total intervention: 3505  Event control: 60  Total control: 3491  *Crude data, female*  Event intervention: 62  Total intervention: 3493  Event control: 54  Total control: 3469 | **1 vs 0 dose, 4-9 months:** HR 1.10 (0.66-1.83)  **9-36 months:** HR 1.01 (0.66-1.56)  No site- or sex-differential effect. | None |
| 7: V. Do et al, 2017 | Data from #10. No placebo, not fully blinded | 4-9 months | 1625 children, 4.5-9 months | Refer to #6 | Data extraction by LF and AC in August 2022.  Eligible for inclusion: Yes  Eligible for analysis: No | **General morbidity, HR**  **Diarrhoea:** 0.89 (0.82-0.97)  **Vomiting:** 0.86 (0.75-0.98) | **General morbidity, males**  **Diarrhoea:** 0.87 (0.78–0.98)  **Vomiting:** 0.78 (0 .66–0.92)  **Coughing:** 0.88 (0.80–0.98)  **Reported fever:** 0.89 (0.82–0.98) | None |
| 8: A. Schoeps et al, 2018 | Data from #6. No placebo, not fully blinded | 4-36 months | 4559 children, 121-215 days | Refer to #6 | Data extraction by LF and AC in August 2022.  Eligible for inclusion: Yes  Eligible for analysis: Yes. | **Composite mortality and admissions:**  **ITT composite:** 1.05 (0.92–1.21) SEM 0.0699  PP composite: 1.00 (0.83–1.20)  *Crude data*  Event intervention: 233  Total intervention: 2258  Event control: 231  Total control: 2238  PP deaths:  Intervention: 37  Control: 39  **PP deaths subtracted:**  *Crude data*  Event intervention: 196  Total intervention: 2258  Event control: 192  Total control: 2238 | **Sub-analyses, PP:**  **Before 9 months:**  1.06 (0.77–1.45)  **After 9 months:** 1.01 (0.80–1.27)  **Female:** 1.16 (0.88–1.53)  **Male:** 0.88 (0.69–1.13) | None |
| 9: M. Steiniche et al, 2020 | Data from #6. No placebo, not fully blinded | 4-9 months | 3750 children, 121-215 days | Refer to #6 | Data extraction by LF and AC in August 2022.  Eligible for inclusion: Yes  Eligible for analysis: No | **Consultations, HR**  **Reported:** 1.03 (0.91–1.15)  **Identified:** 1.04 (0.90–1.19)  *Crude data, male*  Event intervention: 269  Total intervention: 895  Event control: 276  Total control: 888  *Crude data, female*  Event intervention: 238  Total intervention: 875  Event control: 215  Total control: 890  **More severe illness leading to admissions**  **Reported:** 0.83 (0.47–1.46)  **Identified:** 0.64 (0.35–1.16) | **Female:** 1.02 (0.86–1.21)  **Male:** 1.03 (0.88–1.21)  **Age:** 1.03 (0.91–1.15)  **Omit first 14 days:** 1.01 (0.89–1.14)  **Dry** **season:** 1.11 (0.94–1.32)  **Rainy season:** 0.96 (0.82-1.12) | None |
| 10: S. Nielsen et al, 2022 | Original RCT. No placebo, not fully blinded | 4-60 months | 6636 children, 121-215 days | Refer to #6 | Data extraction by LF and AC in August 2022.  Eligible for inclusion: Yes  Eligible for analysis: Yes. | **Mortality, 2 dose vs 1 dose, HR**  **4-60 months:** 1.38 (0.92−2.06) SEM 0.2056  *Crude data*  Event intervention: 90  Total intervention: 4397  Event control: 33  Total control: 2201  *Crude data, male*  Event intervention: 50  Total intervention: 2302  Event control: 19  Total control: 1151  *Crude data, female*  Event intervention: 40  Total intervention: 2095  Event control: 14  Total control: 1050  **9−60 months:** 1.60 (0.99−2.59)  **4−9 months:** 1.29 (0.46−3.62) | No significant difference for  sex and season.  Significant difference for C-OPV  **1 dose vs 0 dose:**  0.94 (0.45–1.96) | None |
| 11: A. Varma et al, 2020 | From RECAMP RCT. Unblinded | 18 months | 8996 children out of 12183 from RECAMP, 0-59 months of age | Standard dose of the EZ strain from Serum Institute of India to children in the intervention group | Data extraction by LF and AC in August 2022.  Eligible for inclusion: Yes  Eligible for analysis: Yes. | **Outpatient consultations, RR**  **Primary:** 0.84 (0.65–1.11) SEM 0.1365  *Crude data*  Event intervention: 322  Total intervention: 4437  Event control: 330  Total control: 3882  *Crude data, male*  Event intervention: 163  Total intervention: 2275  Event control: 169  Total control: 2007  *Crude data, female*  Event intervention: 159  Total intervention: 2162  Event control: 161  Total control: 1875  **Documented:** 0.66 (0.43–1.03) | Caused by respiratory symptoms: 0.68 (0.42–1.11) | None |
| 12: A. Hennino et al, 2007 | Original RCT. Double blinded | 10-20 months | 12 children | ROUVAX (attenuated MV, vaccine strain Schwartz (‡1000 DICC 50), Laboratory AventisPasteur MSD, Lyon, France) | Data extraction by LF and AC in August 2022.  Eligible for inclusion: Yes  Eligible for analysis: No | **Atopy, atopic dermatitis**  No difference in evolution of atopic dermatitis in infants after measles vaccination |  | None |
| 13: S. Byberg et al, 2021 | Original RCT. No placebo, not fully blinded | 9-60 months | 4767 children 9-35 months | EZ, Serum  Institute of India, medium titre  The 182 rural clusters were randomised 1:1 to either ‘MV-for-all-policy’ or ‘Restrictive-MV-policy’ | Data extraction by LF and AC in August 2022.  Eligible for inclusion: Yes  Eligible for analysis: Yes. | **Mortality, ‘**MV for all- versus ‘Restrictive MV policy’, HR  **Primary:** 0.95 (0.64-1.43) SEM 0.2051  *Crude data*  Event intervention: 92  Total intervention: 2428  Event control: 81  Total control: 2339  **Female:** 1.20 (0.63-2.27)  **Male:** 0.78 (95% CI 0.42-1.45)  **Hospital admissions,**  **12–35 months**: 1.09 (0.66-1.79) SEM 0.2545  *Crude data*  Event intervention: 68  Total intervention: 2428  Event control: 65  Total control: 2339 | **After versus before MV campaigns:** 0.45 (0.28-0.75)  **After versus before OPV campaigns:** 0.81 (0.45-1.45)  **After versus before the MenAfriVac campaign:** 1.11 (0.48-2.58). | None |
| 14: M. Berendsen et al, 2022 | Original RCT. No placebo, not fully blinded | 17.5-48 months | 3164 children, 17.5-48 months | Standard titre EZ strain from Serum Institute of India | Data extraction by LF and AC in August 2022.  Eligible for inclusion: Yes  Eligible for analysis: Yes. | **Overall mortality, HR**: 0.50 (0.04-5.46) SEM 1.2542  *Crude data*  Event intervention: 18  Total intervention: 1566  Event control: 25  Total control: 1598  **Admissions, HR:** 0.71 (0.36-1.38) SEM 0.3428  **Severe morbidity, SMRR:** 0.72 (0.38-1.38) | No difference in time, sex, OPV, season | None |
| 15: P. Aaby et al, 1993 | RCT. Data from 2 trials 1984-1985 and 1986-1987 | 4-3 months | 384 children, 4 months | EZ-High titre or IPV for control at 4 months. At 9 months EZ group got IPV and IPV group got SW-ST | Data extraction by LF and AC in August 2022.  Eligible for inclusion: Yes  Eligible for analysis: Yes | **Mortality, MR**  **Censoring migrations:**  1.79 (1.06-3.02)  **Not censoring migrations:** 1.53 (0.94-2.49)  **Adjusted:** 1.56 (0.96-2.55)  *Crude data*  Event intervention: 42  Total intervention: 201  Event control: 26  Total control: 183  *Crude data, female*  Event intervention: 31  Total intervention: 104  Event control: 16  Total control: 98 | **Female:** 1.95 (1.07-3.56)  **Male:** 0.98 (0.41-2.30) | None |
| 16: P. Aaby et al,1994 | RCT. Data from 1984-1985 (refer to #15) | 4-60 months | 470 children, 4 months | EZ-High titre or IPV for control at 4 months. At 9 months EZ group got IPV and IPV group got SW-ST | Data extraction by LF and AC in August 2022.  Eligible for inclusion: Yes  Eligible for analysis: Yes. | **Mortality, MR**  **Censoring migrations:**  0.82 (0.48-1.41)  **Not censoring migrations:** 1.07 (0.67-1.70)  *Crude data*  Event intervention: 37  Total intervention: 234  Event control: 35  Total control: 236  *Crude data, female*  Event intervention: 11  Total intervention: 103  Event control: 18  Total control: 121 | There were no significant mortality differences by sex,  season, and measles infection when controlled for age. | None |
| 17: Libman et al, 2002 | RCT. Original RCT | 18-60 months | 510 children, 5 months | **Group 1:** meningococcal vaccine 5 months, SW-ST 9 months  **Group 2:** EZ-HT vaccine 5 months, meningococcal vaccine 9 months  **Group 3:** increased titre Connaught vaccine 5 months, SW-ST 9 months | Data extraction by LF and AC in August 2022.  Eligible for inclusion: Yes  Eligible for analysis: Yes. | No significant differences in morbidity or mortality between vaccine groups either during the first 4 months after vaccination or later in the study.  *Crude data*  Event intervention: 23  Total intervention: 323  Event control: 13  Total control: 148  *Crude data, female*  Event intervention: 13  Total intervention: 140  Event control: 6  Total control: 75 | Stratification by sex and age did not reveal any suggestion of significantly increased morbidity or mortality in either sex at any age attributable to the vaccine group. | None |
| 18: E. Holt et al, 1993 | RCT, reanalysis of 1987-1988 RCT | 6-36 months | 1972 children, 6-11 months | Either SW-ST, SW-HT, EZ-ST, or EZ-HT at inclusion vs only SW-ST at 9 months | Data extraction by LF and AC in August 2022.  Eligible for inclusion: Yes  Eligible for analysis: Yes. | **Mortality, Medium versus high, MRR**  *Crude data*  Event intervention: 40  Total intervention: 994  Event control: 35  Total control: 978  **Female:** 1.71 (0.91-3.24)  *Crude data*  Event intervention: 26  Total intervention: 512  Event control: 15  Total control: 475  **Male:** 0.71 (0.36-11.40) |  | None |
| 19: Garenne et al, 1991 | RCT, interim analysis from RCT 1987-1989 | 5-44 months | 1015 children, 5-44 months | **High titre group:** EZ-HT or Schwarz SW-HT at 5 months of age  **Standard-titre group:** placebo at 5 months of age and SW-std at 10 months | Data extraction by LF and AC in August 2022.  Eligible for inclusion: Yes  Eligible for analysis: No | **Mortality, RR**  **EZ-HT vs standard:**  1.80 (1.18-2.74)  **SW-HT vs. standard:** 1.51 (0.97-2.34)  **EZ-H vs SW-HT:** No difference | **Sex, RR, female versus male**  **EZ-HT group:** 1.20 p=0.497  **SW-HT group:** 1.14 p=0.636  **Std group:** 0.64 **Non-participants:** 1.16 | None |
| 20: P. Aaby et al, 1994 | RCT, final report from #19 | 5-44 months | 2177 children, 5-44 months | Refer to #19 | Data extraction by LF and AC in August 2022.  Eligible for inclusion: Yes  Eligible for analysis: Yes. | **Mortality, MR**  **EZ-HT vs standard:** 1.32 (0.96-1.82)  *Crude data*  Event intervention: 134  Total intervention: 945  Event control: 67  Total control: 634  *Crude data, female*  Event intervention: 78  Total intervention: 495  Event control: 29  Total control: 321  ***At 36 months:*** 1.17 (0.84-1.62)  **SW-HT vs standard:** 1.45 (0.94-2.23) | **Mortality, MR, female**  **SWHT vs standard**: 2.14 (1.12-4.09) | None |
| 21: P. Aaby et al, 1996 | RCT, re-analysis from #20 | 5-44 months | 2177 children, 5-44 months | Refer to #20 | Data extraction by LF and AC in August 2022.  Eligible for inclusion: Yes  Eligible for analysis: No | **Mortality:**  At this age there was no difference in mortality between female recipients of high-titre and standard titre measles vaccines |  | None |
| 22: P. Aaby et al, 2007 | RCT, reanalysis from four different RCT 1980’s and 1990’s | N/A | 9294 children | Four trials with four different designs  1 and 2: EZ-ST at 4 months and IPV at 9 months vs IPV at 4 months and SW-ST at 9 months  3 and 4: SW-ST at six and 9 months vs IPV at six months and SW-ST at 9 months | Data extraction by LF and AC in August 2022.  Eligible for inclusion: Yes  Eligible for analysis: No | **Mortality, female/male MRR**  **IPV group:** 1.52 (1.02–2.28)  **MV group:** (1.01, (0.69 –1.46)  IPV group after receiving MV at 9 months or later: 0.88 (0.68 –1.14) |  | None |
| 23: A. Zimakoff et al, 2023 | RCT, original trial | 5-12 months | 6540 infants, 5-7 months | **Intervention:**  M-M-R VaxPro vaccine  **Control:** placebo | Data extraction by LF and AC in August 2022.  Eligible for inclusion: Yes  Eligible for analysis: Yes. | **Morbidity, hospitalisations for infection, HR**  **Intervention vs control. 1 dose vs 0 dose:** 1.03 (0.91-1.18) SEM 0.0663  *Crude data*  Event intervention: 786  Total intervention: 3264  Event control: 762  Total control: 3272 | **Hospitalisations >**  **12 hours:**  1.25 (0.88-1.77)  ***Adjusted:*** 1.24 (0.87-1.76).  **Time to first hospitalisation**  **for infection:** 1.01 (0.90-1.13)  **Prescriptions for a systemic**  **antibiotics:** 1.04 (0.88-1.23) | None |

S4 Table. Includes all data extracted from all included trials.
