# Supplementary material for: Measles vaccines and non-specific effects on mortality or morbidity: A systematic review and meta-analysis
Source: PLoS One. 2025 Jul 2;20(7):e0321982. doi: 10.1371/journal.pone.0321982 (PMC12221017; doi:10.1371/journal.pone.0321982)
Supplement: S5 Table — (DOCX) [file pone.0321982.s008.docx]

**S5 Table. Exclusion reasons for all excluded studies after full text screening**

| Author | Article name | Exclusion reason |
| --- | --- | --- |
| Hviid, A.; Melbye, M. | Measles-mumps-rubella vaccination and asthma-like disease in early childhood | Observational study design |
| P. J. WILLIAMS, Ministry of Health, Environment, Labour and Social Welfare, Banjul, Gambia | EFFECT OF MEASLES IMMUNIZATION ON CHILD MORTALITY IN RURAL GAMBIA | Observational study design |
| Peter Aaby, Andreas Andersen, Cesário L Martins, Ane B Fisker, Amabelia Rodrigues, Hilton C Whittle, Christine S Benn | Does oral polio vaccine have non-specific effects on all-cause mortality? Natural experiments within a randomised controlled trial of early measles vaccine | Wrong intervention |
| J Agergaard 1, E Nante, G Poulstrup, J Nielsen, K L Flanagan, L Østergaard, C S Benn, P Aaby | Diphtheria-tetanus-pertussis vaccine administered simultaneously with measles vaccine is associated with increased morbidity and poor growth in girls. A randomised trial from Guinea-Bissau. | Wrong intervention |
| M Garenne 1, O Leroy, J P Beau, I Sene | Child mortality after high-titre measles vaccines: prospective study in Senegal | Duplicate |
| Fidel PL Jr; Noverr MC | Could an Unrelated Live Attenuated Vaccine Serve as a Preventive Measure To Dampen Septic Inflammation Associated with COVID-19 Infection? | Commentary |
| Elguero E; Simondon KB; Vaugelade J; Marra A; Simondon F | Non-specific effects of vaccination on child survival? A prospective study in Senegal. | Commentary |
| Koenig, M. A.; Khan, M. A.; Wojtyniak, B.; Clemens, J. D.; Chakraborty, J.; Fauveau, V.; Phillips, J. F.; Akbar, J.; Barua, U. S. | Impact of measles vaccination on childhood mortality in rural Bangladesh | Observational study design |
| Shann F | The heterologous (non-specific) effects of vaccines: implications for policy in high-mortality countries. | Commentary |
| Aaby P; Nielsen J; Benn CS; Trape JF | Sex-differential and non-specific effects of routine vaccinations in a rural area with low vaccination coverage: an observational study from Senegal. | Observational study design |
| Clemens, J. D.; Stanton, B. F.; Chakraborty, J.; Chowdhury, S.; Rao, M. R.; Ali, M.; Zimicki, S.; Wojtyniak, B. | Measles vaccination and childhood mortality in rural Bangladesh | Observational study design |
| Mommers, M.; Weishoff-Houben, M.; Swaen, G. M. H.; Creemers, H.; Freund, H.; Dott, W.; Van Schayck, C. P. | Infant immunization and the occurrence of atopic disease in Dutch and German children: A nested case-control study | Observational study design |
| Kabir Z; Long J; Reddaiah VP; Kevany J; Kapoor SK | Non-specific effect of measles vaccination on overall child mortality in an area of rural India with high vaccination coverage: a population-based case-control study. | Observational study design |
| Paunio, M.; Peltola, H.; Virtanen, M.; Leinikki, P.; Makela, A.; Heinonen, O. P. | Acute infections, infection pressure, and atopy | Observational study design |
| Benn CS; Aaby P; Særup S | Non-specific effects of vaccination in Denmark | Commentary |
| Anbarasu A; Ramaiah S; Livingstone P | Vaccine repurposing approach for preventing COVID 19: can MMR vaccines reduce morbidity and mortality? | Commentary |
| Zimmermann P; Perrett KP; van der Klis FR; Curtis N | The immunomodulatory effects of measles-mumps-rubella vaccination on persistence of heterologous vaccine responses. |  |
| Libraty, D. H. | Effect of an Early Dose of Measles Vaccine on Morbidity Between 18 Weeks and 9 Months of Age: A Randomized, Controlled Trial in Guinea-Bissau | Commentary |
| Diaz-Ortega, J. L.; Luna-Abascal, M.; Valdespino, J. L.; Sepulveda, J.; Markowitz, L. E.; Zell, E. R. | Mortality and morbidity after high titre measls vaccine in Mexico | Commentary |
| Nono, J. K.; Kamdem, S. D.; Netongo, P. M.; Dabee, S.; Schomaker, M.; Oumarou, A.; Brombacher, F.; Moyou-Somo, R. | Schistosomiasis Burden and Its Association With Lower Measles Vaccine Responses in School Children From Rural Cameroon | Observational study design |
| Tanday, S. | MMR lowers infection-related hospital admissions in children | Commentary |
| Dulny, G.; Sybilski, A. J.; Zalewska, M.; Raciborski, F.; Komorowski, J.; Piekarska, B.; Lipiec, A.; Samolinski, B. | The effect of preventive immunization on the incidence of allergic conditions | Population>18 years |
| Rudan, I.; Boschi-Pinto, C.; Biloglav, Z.; Mulholland, K.; Campbell, H | Epidemiology and etiology of childhood pneumonia | Observational study design |
| Shann F | Immunization--dramatic new evidence. | Commentary |
| McGovern, M. E.; Canning, D. | Vaccination and All-Cause Child Mortality From 1985 to 2011: Global Evidence From the Demographic and Health Surveys | Observational study design |
| Mysore V; Cullere X; Settles ML; Ji X; Kattan MW; Desjardins M; Durbin-Johnson B; Gilboa T; Baden LR; Walt DR; Lichtman AH; Jehi L; Mayadas | Protective heterologous T cell immunity in COVID-19 induced by the trivalent MMR and Tdap vaccine antigens. | Wrong outcome |
| Flöistrup, H.; Swartz, J.; Bergström, A.; Alm, J. S.; Scheynius, A.; van Hage, M.; Waser, M.; Braun-Fahrländer, C.; Schram-Bijkerk, D.; Huber, M.; Zutavern, A.; von Mutius, E.; Ublagger, E.; Riedler, J.; Michaels, K. B.; Pershagen, G. | Allergic disease and sensitization in Steiner school children | Observational study design |
| Du Lou, A. D.; Pison, G.; Aaby, P. | Role of immunizations in the recent decline in childhood mortality and the changes in the female/male mortality ratio in rural Senegal | Observational study design |
| Alemayehu, K.; Oljira, L.; Demena, M.; Birhanu, A.; Workineh, D. | Prevalence and Determinants of Diarrheal Diseases among Under-Five Children in Horo Guduru Wollega Zone, Oromia Region, Western Ethiopia: A Community-Based Cross-Sectional Study | Observational study design |
| Mina M.J.; Metcalf C.J.E.; De Swart R.L.; Osterhaus A.D.M.E.; Grenfell B.T. | Long-term measles-induced immunomodulation increases overall childhood infectious disease mortality | Commentary |
| Altulayhi, R. I.; Alqahtani, R. M.; Alakeel, R. A.; Khorshid, F. A.; Alshammari, R. H.; Alattas, S. G.; Alshammari, F. A. M.; Bin-Jumah, M.; Abdel-Daim, M. M.; Almohideb, M. | Correlation between measles immunization coverage and overall morbidity and mortality for COVID-19: an epidemiological study | Observational study design |
| Sato, R.; Haraguchi, M. | Effect of measles prevalence and vaccination coverage on other disease burden: evidence of measles immune amnesia in 46 African countries | Observational study design |
| Anderson, H. R.; Poloniecki, J. D.; Strachan, D. P.; Beasley, R.; Björkstén, B.; Asher, M. I. | Immunization and symptoms of atopic disease in children: results from the International Study of Asthma and Allergies in Childhood | Observational study design |
| Shohat, T.; Green, M. S.; Nakar, O.; Ballin, A.; Duvdevani, P.; Cohen, A.; Shohat, M. | Gender differences in the reactogenicity of measles-mumps-rubella vaccine | Wrong outcome |
| Nishi, M.; Miyake, H. | A case-control study of non-T cell acute lymphoblastic leukaemia of children in Hokkaido, Japan | Observational study design |
| Miller, E.; Andrews, N.; Waight, P.; Taylor, B | Bacterial infections, immune overload, and MMR vaccine | Observational study design |
| Van Balen, H.; Mercenier, P.; Daveloose, P. | Influence of measles vaccination on survival pattern of 7-35-month-old children in Kasongo, Zaire | Observational study design |
| Wickens, K.; Crane, J.; Kemp, T.; Lewis, S.; D'Souza, W.; Sawyer, G.; Stone, L.; Tohill, S.; Kennedy, J.; Slater, T.; Rains, N.; Pearce, N. | A case-control study of risk factors for asthma in New Zealand children | Observational study design |
| Nagel, G.; Weinmayr, G.; Flohr, C.; Kleiner, A.; Strachan, D. P. | Association of pertussis and measles infections and immunizations with asthma and allergic sensitization in ISAAC Phase Two | Observational study design |
| Ogimi, C.; Qu, P.; Boeckh, M.; Bender Ignacio, R. A.; Zangeneh, S. Z. | Association between live childhood vaccines and COVID-19 outcomes: a national-level analysis | Observational study design |
| Aaby, P.; Pedersen, I. R.; Knudsen, K.; da Silva, M. C.; Mordhorst, C. H.; Helm-Petersen, N. C.; Hansen, B. S.; Thårup, J.; Poulsen, A.; Sodemann, M.; et al., | Child mortality related to seroconversion or lack of seroconversion after measles vaccination | Wrong outcome |
| Hviid, A.; Hansen, J. V.; Frisch, M.; Melbye, M. | Measles, Mumps, Rubella Vaccination and Autism: A Nationwide Cohort Study | Observational study design |
| Aaby, P.; Samb, B.; Simondon, F.; Knudsen, K.; Seck, A. M.; Bennett, J.; Markowitz, L.; Whittle, H | A comparison of vaccine efficacy and mortality during routine use of high-titre Edmonston-Zagreb and Schwarz standard measles vaccines in rural Senegal | Observational study design |
| Whittle, H.; Hanlon, P.; O'Neill, K.; Hanlon, L.; Marsh, V.; Jupp, E.; Aaby, P. | Trial of high-dose Edmonston-Zagreb measles vaccine in the Gambia: antibody response and side-effects | Wrong outcome |
| Miller, E.; Andrews, N.; Grant, A.; Stowe, J.; Taylor, B. | No evidence of an association between MMR vaccine and gait disturbance | Observational study design |
| Wilson, K.; Ducharme, R.; Ward, B.; Hawken, S. | Increased emergency room visits or hospital admissions in females after 12-month MMR vaccination, but no difference after vaccinations given at a younger age | Observational study design |
| Bernsen, R. M.; van der Wouden, J. C | Measles, mumps and rubella infections and atopic disorders in MMR-unvaccinated and MMR-vaccinated children | Observational study design |
| Korbely, C.; Weinberger, A.; Kutzora, S.; Huß, J.; Hendrowarsito, L.; Nennstiel, U.; Heißenhuber, A.; Herr, C.; Heinze, S. | Atopic diseases and airway-related symptoms in Bavarian pre-schoolers: determinants and association with immunization | Observational study design |
| Deng, L.; Macartney, K.; Gill, D.; Fathima, P.; Wood, N.; Gidding, H. | Status epilepticus outcomes among vaccinated and unvaccinated children: A population-based study | Observational study design |
| Mullooly, J. P.; Schuler, R.; Barrett, M.; Maher, J. E. | Vaccines, antibiotics, and atopy | Observational study design |
| Biai, S.; Rodrigues, A.; Nielsen, J.; Sodemann, M.; Aaby, P. | Vaccination status and sequence of vaccinations as risk factors for hospitalisation among outpatients in a high mortality country | Observational study design |
| Seagroatt, V.; Goldacre, M. J. | Crohn's disease, ulcerative colitis, and measles vaccine in an English population, 1979-1998 | Observational study design |
| Hirve S; Bavdekar A; Juvekar S; Benn CS; Nielsen J; Aaby P | Non-specific and sex-differential effects of vaccinations on child survival in rural western India. | Observational study design |
| Roost, H. P.; Gassner, M.; Grize, L.; Wüthrich, B.; Sennhauser, F. H.; Varonier, H. S.; Zimmermann, H.; Braun-Fahrländer, Ch | Influence of MMR-vaccinations and diseases on atopic sensitization and allergic symptoms in Swiss schoolchildren | Observational study design |
| Deng, L.; Danchin, M.; Lewis, G.; Wen, S. C. H.; Doyle, R.; Barnett, M.; Campbell, A. J.; Wadia, U.; Ewe, K.; Bhatia, R.; Wood, N. | Status epilepticus following vaccination in children aged <=24months: A five-year retrospective observational study | Observational study design |
| Geier, D. A.; Geier, M. R. | A longitudinal cohort study of childhood MMR vaccination and seizure disorder among American children | Observational study design |
| Maclure, A.; Stewart, G. T. | Admission of children to hospitals in Glasgow: relation to unemployment and other deprivation variables | Observational study design |
| Timmermann, C. A.; Osuna, C. E.; Steuerwald, U.; Weihe, P.; Poulsen, L. K.; Grandjean, P. | Asthma and allergy in children with and without prior measles, mumps, and rubella vaccination | Observational study design |
| Olesen, A. B.; Juul, S.; Thestrup-Pedersen, K. | Atopic dermatitis is increased following vaccination for measles, mumps and rubella or measles infection | Observational study design |
| Ogimi, C.; Qu, P.; Boeckh, M.; Bender Ignacio, R. A.; Zangeneh, S. Z. | Association between live childhood vaccines and COVID-19 outcomes: a national-level analysis | Observational study design |
| Benn C.S.; Martins C.L.; Andersen A.; Fisker A.B.; Whittle H.C.; Aaby P. | Measles Vaccination in Presence of Measles Antibody May Enhance Child Survival | Wrong intervention |
| PrayGod, G.; Mukerebe, C.; Magawa, R.; Jeremiah, K.; Török, M. E. | Indoor Air Pollution and Delayed Measles Vaccination Increase the Risk of Severe Pneumonia in Children: Results from a Case-Control Study in Mwanza, Tanzania | Observational study design |
| Pawlowski, C.; Puranik, A.; Bandi, H.; Venkatakrishnan, A. J.; Agarwal, V.; Kennedy, R.; O'Horo, J. C.; Gores, G. J.; Williams, A. W.; Halamka, J.; Badley, A. D.; Soundararajan, V. | Exploratory analysis of immunization records highlights decreased SARS-CoV-2 rates in individuals with recent non-COVID-19 vaccinations | Observational study design |
| Timmermann, C. A.; Budtz-Jørgensen, E.; Jensen, T. K.; Osuna, C. E.; Petersen, M. S.; Steuerwald, U.; Nielsen, F.; Poulsen, L. K.; Weihe, P.; Grandjean, P. | Association between perfluoroalkyl substance exposure and asthma and allergic disease in children as modified by MMR vaccination | Observational study design |
| Newcomer, S. R.; Daley, M. F.; Narwaney, K. J.; Xu, S.; DeStefano, F.; Groom, H. C.; Jackson, M. L.; Lewin, B. J.; McLean, H. Q.; Nordin, J. D.; Zerbo, O.; Glanz, J. M. | Order of Live and Inactivated Vaccines and Risk of Non-vaccine-targeted Infections in US Children 11-23 Months of Age | Observational study design |
| Stowe, J.; Andrews, N.; Taylor, B.; Miller, E. | No evidence of an increase of bacterial and viral infections following Measles, Mumps and Rubella vaccine | Observational study design |
| Lewis, S. A.; Britton, J. R. | Measles infection, measles vaccination and the effect of birth order in the aetiology of hay fever | Observational study design |
| DeStefano, F.; Gu, D.; Kramarz, P.; Truman, B. I.; Iademarco, M. F.; Mullooly, J. P.; Jackson, L. A.; Davis, R. L.; Black, S. B.; Shinefield, H. R.; Marcy, S. M.; Ward, J. I.; Chen, R. T. | Childhood vaccinations and risk of asthma | Observational study design |
| Byberg S; Østergaard MD; Rodrigues A; Martins C; Benn CS; Aaby P; Fisker AB | Analysis of risk factors for infant mortality in the 1992-3 and 2002-3 birth cohorts in rural Guinea-Bissau. | Observational study design |
| Hassani D; Amiri MM; Maghsood F; Salimi V; Kardar GA; Barati O; Hashemian SMR; Jeddi-Tehrani M; Zarnani AH; Shokri F | Does prior immunization with measles, mumps, and rubella vaccines contribute to the antibody response to COVID-19 antigens? | Observational study design |
