# Supplementary material for: Measles vaccines and non-specific effects on mortality or morbidity: A systematic review and meta-analysis
Source: PLoS One. 2025 Jul 2;20(7):e0321982. doi: 10.1371/journal.pone.0321982 (PMC12221017; doi:10.1371/journal.pone.0321982)
Supplement: S6 Table — (DOCX) [file pone.0321982.s009.docx]

**S6 Table: Decisions made in the TSA program in chronological order**

| **Parameter** | **Chosen option** |
| --- | --- |
| Effect measure | Relative risk (RR) |
| Model | Random-Effects (DL) |
| **Set zero event handling** |  |
| Method | Constant |
| Value | 0.5 |
| Include trials with no events | Yes |
| Set confidence intervals | Conventional 95% |
| **Trials** |  |
| All included trials marked as high risk of bias | |
| **Conventional test boundary** | Twosided, 5% |
| **Alpha-spending boundaries** |  |
| Boundary type | Twosided |
| Type 1 error | 5% |
| Alpha-spending function | O’Brien-Fleming |
| Information axis | Sample size |
| Apply inner wedge | Yes |
| Beta-spending function | O’Brien-Fleming |
| Information size | Estimate |
| Power | 80% |
| Relative Risk Reduction | User defined, 25% |
| Incidence in intervention arm | User defined, No |
| Incidence in control arm | Yes |
| Heterogeneity correction | Model variance based |
| **Law of the Iterated Logarithm** | Not applied |

S6 table: All decisions made when using the TSA software. This table can be used if one wishes to reproduce the TSA analyses made in this paper.
