# Supplementary figures and images for: Measles vaccines and non-specific effects on mortality or morbidity: A systematic review and meta-analysis
Source: PLoS One. 2025 Jul 2;20(7):e0321982. doi: 10.1371/journal.pone.0321982 (PMC12221017; doi:10.1371/journal.pone.0321982)

**Figure S2: Standard titre measles vaccine. Mortality. Two versus one doses. Crude data.**


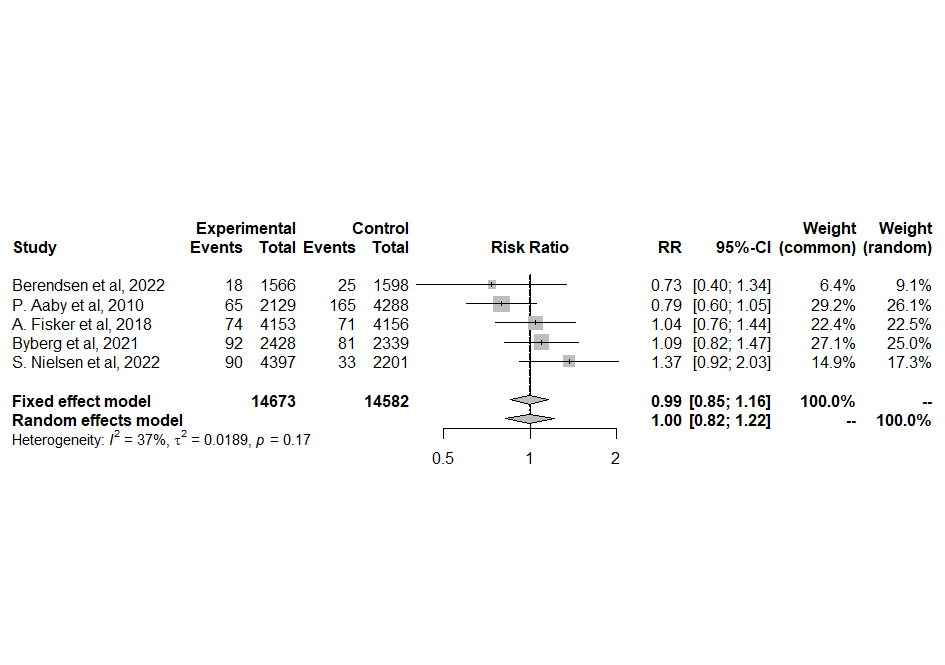

Supplement: S2 Fig — (DOCX) [file pone.0321982.s010.docx]

**Figure S3: Morbidity. Standard titre measles vaccine. Crude data**

**
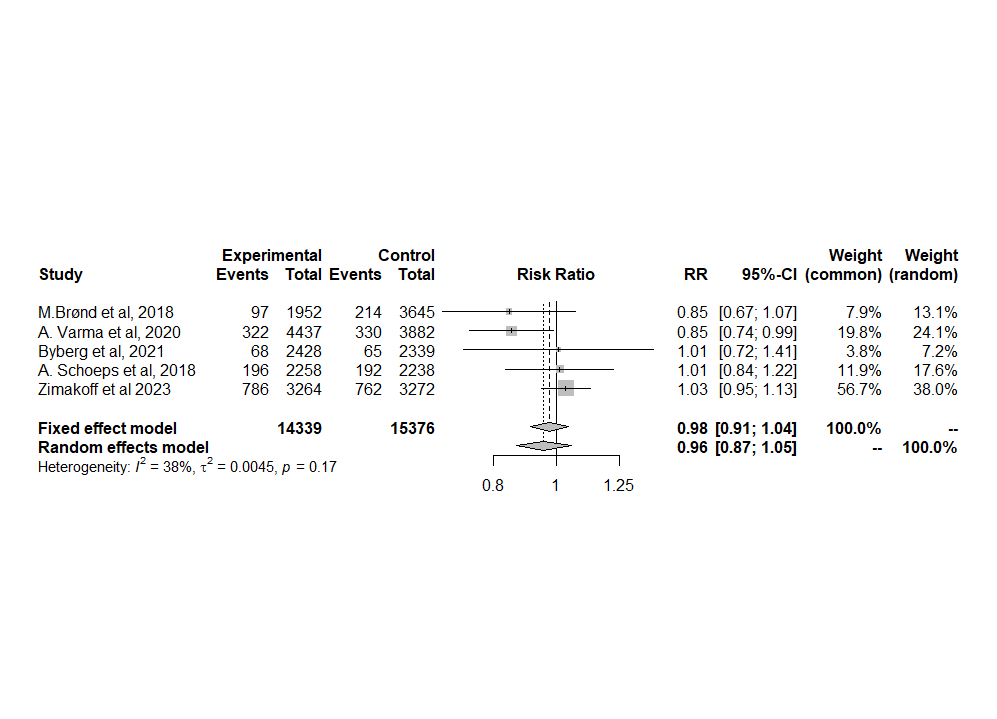
**

Supplement: S3 Fig — (DOCX) [file pone.0321982.s013.docx]
