# Supplementary material for: Measles vaccines and non-specific effects on mortality or morbidity: A systematic review and meta-analysis
Source: PLoS One. 2025 Jul 2;20(7):e0321982. doi: 10.1371/journal.pone.0321982 (PMC12221017; doi:10.1371/journal.pone.0321982)
Supplement: S2 Appendix — (DOCX) [file pone.0321982.s011.docx]

**S2 Appendix. The standard titre measles vaccine. Mortality. One versus zero doses.**

**Figure A: Mortality. Standard titre measles vaccine. One versus zero doses. Crude data**


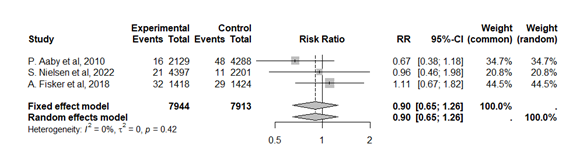


**Figure B: Mortality effects of one dose of standard titre measles vaccine compared to zero doses. Risk ratios (RR) with 95% confidence intervals. RRR=25%**

**
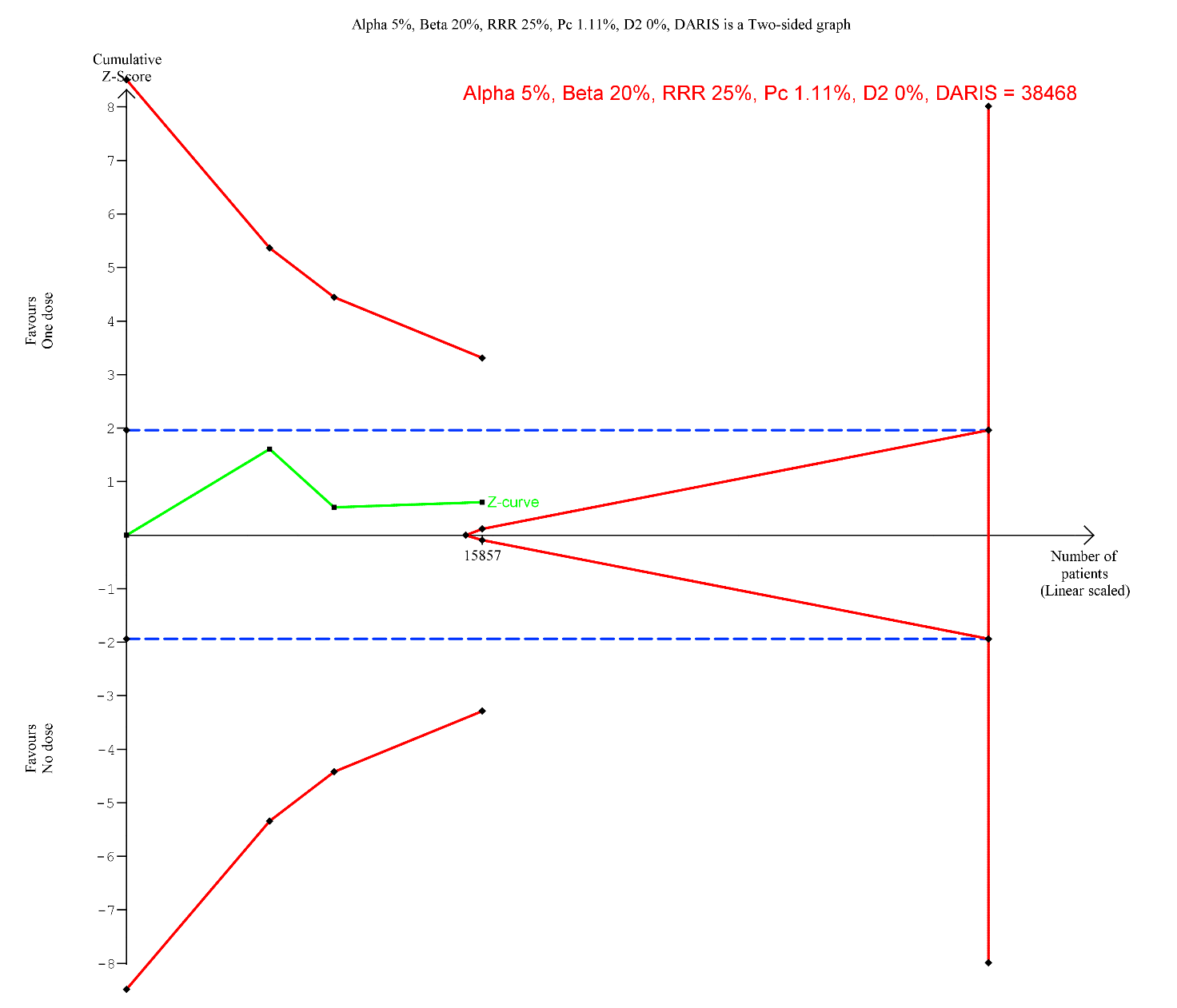
**

Figure B:

Pooled Effect, RR = 0.90 (0.65 to 1.26) p-value: 0.55

Heterogeneity, Q = 1.74 Heterogeneity, Q, p-value = 0.42

Inconsistency, I² = 0.00 Diversity, D² = 0.00

**Figure C: Mortality effects of one dose of standard titre measles vaccine compared to zero doses. Risk ratios (RR) with 95% confidence intervals. RRR=33%**


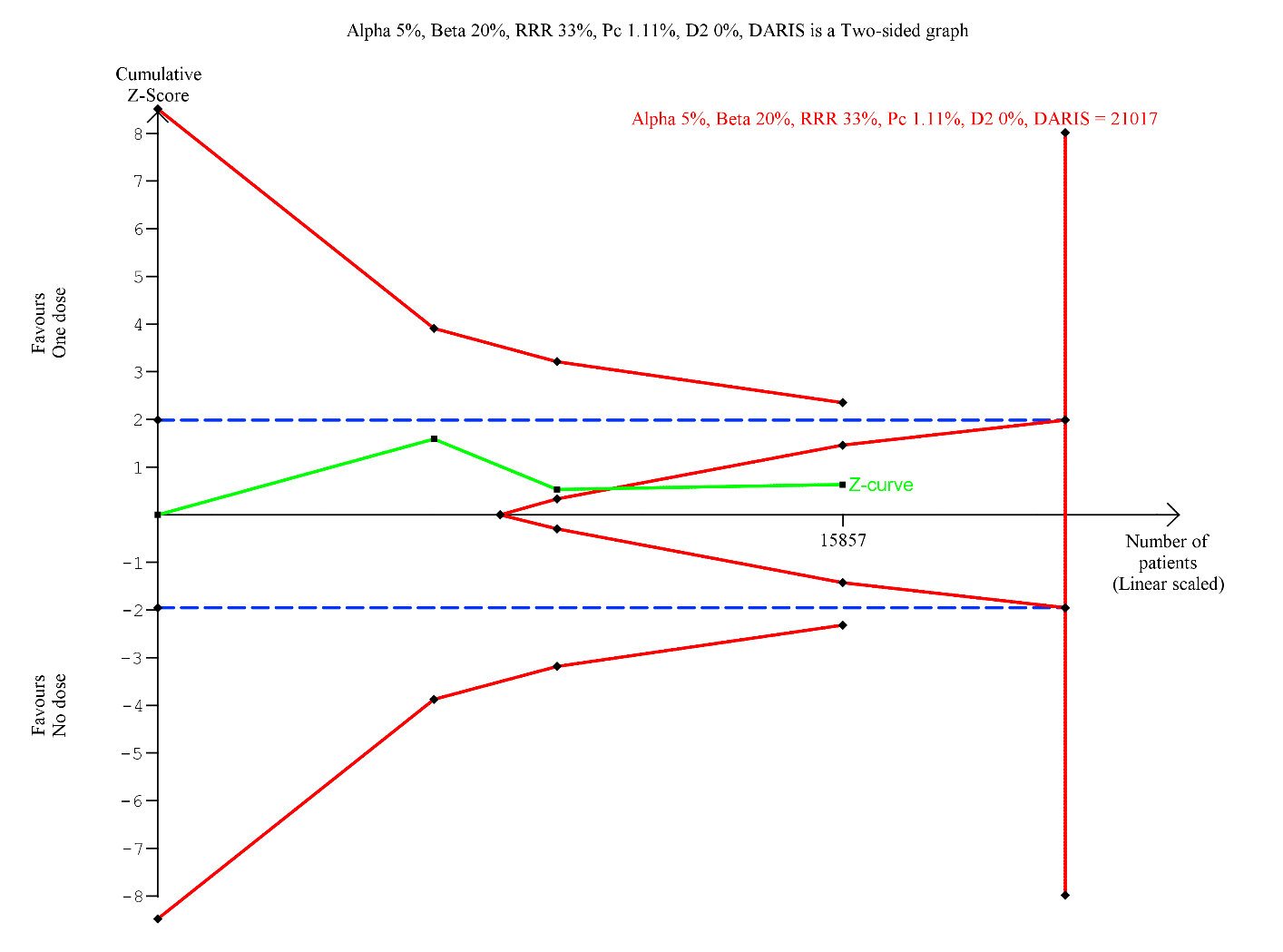


Figure C:

Pooled Effect, RR = 0.90 (0.65 to 1.26) p-value: 0.55

Heterogeneity, Q = 1.74 Heterogeneity, Q, p-value = 0.42

Inconsistency, I² = 0.00 Diversity, D² = 0.00

This meta-analysis has a pooled effect of RR = 0.90 with an insignificant p-value=0.55. The z-curve (green) does not reach the area of futility. Thus, no significant difference between the mortality risks of the two groups were found on a 25% risk reduction level but there was not enough data to reach a conclusion.

Two TSA analyses were made for the mortality effects of one dose versus zero doses of MCV. The meta-analysis did not change as the data were the same in both. The difference was the investigated RRR value. This value was changed from 25% in figure S10a to 33% in figure S10b. This meta-analysis resulted in a pooled effect of RR = 0,90 with an insignificant p-value, p=0.55. Therefore, enough data have been gathered to conclude that no 33% reduction in relative mortality risk can be found. No significant difference between the mortality risks of the two groups were found on the 25% risk reduction level but there was not enough data to reach a conclusion.
