# Supplementary material for: Measles vaccines and non-specific effects on mortality or morbidity: A systematic review and meta-analysis
Source: PLoS One. 2025 Jul 2;20(7):e0321982. doi: 10.1371/journal.pone.0321982 (PMC12221017; doi:10.1371/journal.pone.0321982)
Supplement: S3 Appendix — (DOCX) [file pone.0321982.s012.docx]

**S3 Appendix. The standard titre measles vaccine. Mortality. Potential sex-differential effects**

**Figure A: Mortality effects of a two- versus one-dose program of standard titre measles vaccine. Male infants only. Risk ratios (RR) with 95% confidence intervals.**


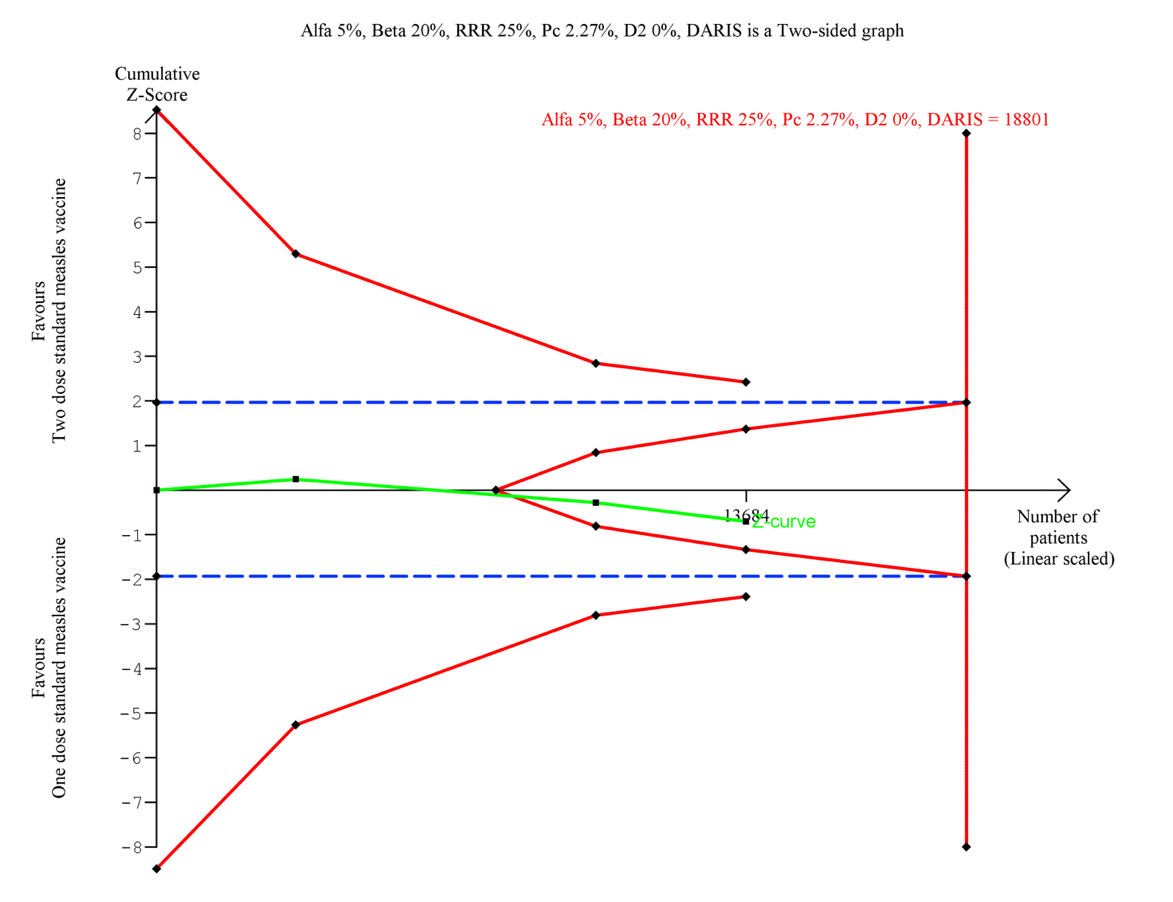


Figure A:

Pooled Effect, RR = 1.09 (0.86 to 1.37) p-value = 0.47

Heterogeneity, Q = 0.97 Heterogeneity, Q, p-value: 0.62

Inconsistency, I² = 0.00 Diversity, D² = 0.00

Mortality effects for male sex only. The pooled effect is RR=1.09 with p = 0.47. The z-curve reaches the area of futility. Thus, no significant difference is found between a two-dose measles vaccine program and a one-dose measles vaccine program when investigating a 25% relative risk reduction level.

**Figure B: Mortality effects of a two- versus one-dose program of standard titre measles vaccine. Female infants only. Risk ratios (RR) with 95% confidence intervals.

*RRR = 25%***

*
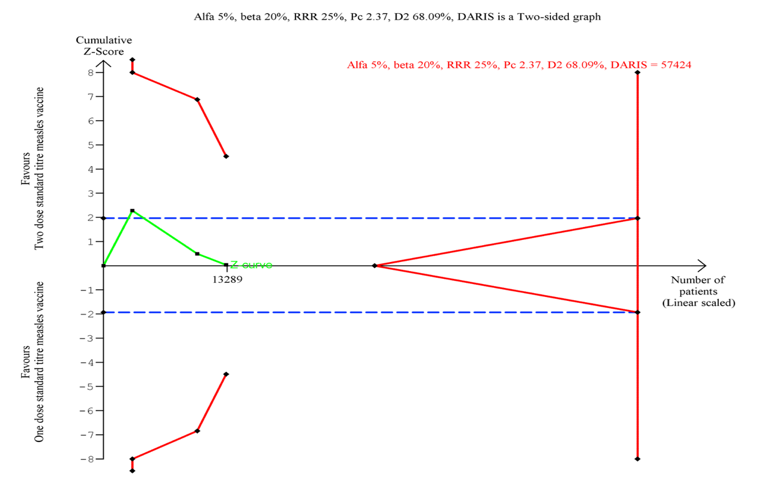
*

***RRR = 33%***


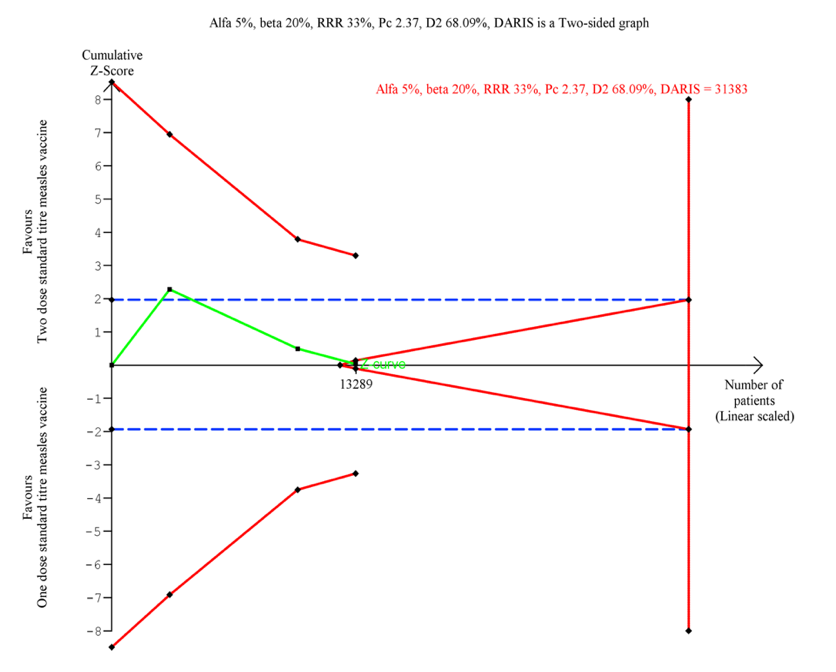


Figure B:
Pooled Effect: 1.00 (0.64 to 1.54) p-value = 0.99

Heterogeneity, Q = 5.75 Heterogeneity, Q, p-value: 0.06

Inconsistency, I² = 0.65 Diversity, D² = 0.68

Two TSA analyses were made for females only. The meta-analysis did not change as the data were the same in both. The difference was the investigated RRR value. This value was changed from 25% in figure S1a to 33% in figure S1b. This meta-analysis resulted in a pooled effect of RR = 1.00 with an insignificant p-value, p=0.985. Therefore, enough data have been gathered on females, to conclude that no 33% reduction in relative mortality risk can be found. No significant difference between the mortality risks of the two groups were found for females on the 25% risk reduction level but there was not enough data to reach a conclusion.
