# Supplementary material for: Measles vaccines and non-specific effects on mortality or morbidity: A systematic review and meta-analysis
Source: PLoS One. 2025 Jul 2;20(7):e0321982. doi: 10.1371/journal.pone.0321982 (PMC12221017; doi:10.1371/journal.pone.0321982)
Supplement: S4 Appendix — (DOCX) [file pone.0321982.s014.docx]

**S4 appendix. The standard titre measles vaccine. Morbidity. One versus zero doses.**

**Figure A: Morbidity. Standard titre measles vaccine. One versus zero doses. Crude data**


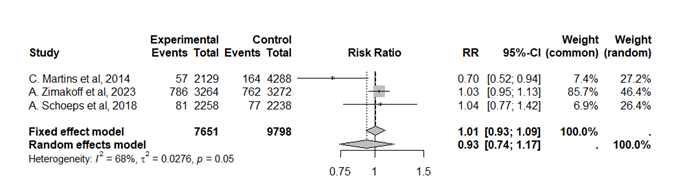


**Figure B: Morbidity effects of one dose of standard titre measles vaccine compared to zero doses. Risk ratios (RR) with 95% confidence intervals.**


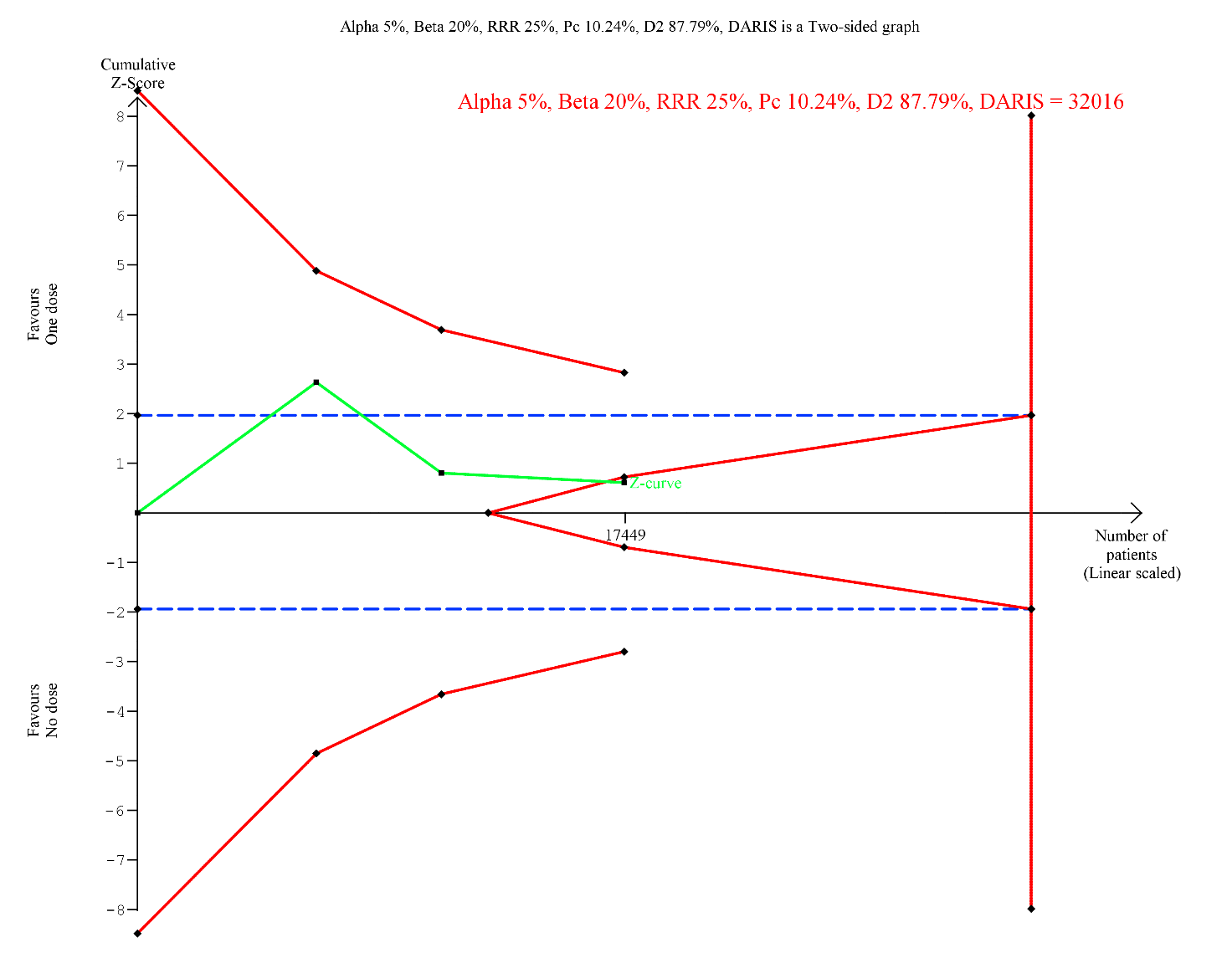


Figure B:

Pooled Effect, RR = 0.93 (0.74 to 1.17) p-value: 0.55

Heterogeneity, Q = 6.21 Heterogeneity, Q, p-value = 0.05

Inconsistency, I² = 0.68 Diversity, D² = 0.88

This meta-analysis has a pooled effect of RR = 0.93 with an insignificant p-value=0.55. The z-curve (green) reaches the area of futility. Thus, the result, that no significant difference between the mortality risks of the two groups were found on a 25% risk reduction level, was conclusive.
