# Supplementary material for: Measles vaccines and non-specific effects on mortality or morbidity: A systematic review and meta-analysis
Source: PLoS One. 2025 Jul 2;20(7):e0321982. doi: 10.1371/journal.pone.0321982 (PMC12221017; doi:10.1371/journal.pone.0321982)
Supplement: S5 Appendix — (DOCX) [file pone.0321982.s015.docx]

**S5 appendix. The standard titre measles vaccine. Morbidity. Potential sex-differential effects.**

**Figure A: Morbidity effects of a two- versus one-dose program of standard titre measles vaccine on female sex only. Risk ratios (RR) with 95% confidence intervals.**

**
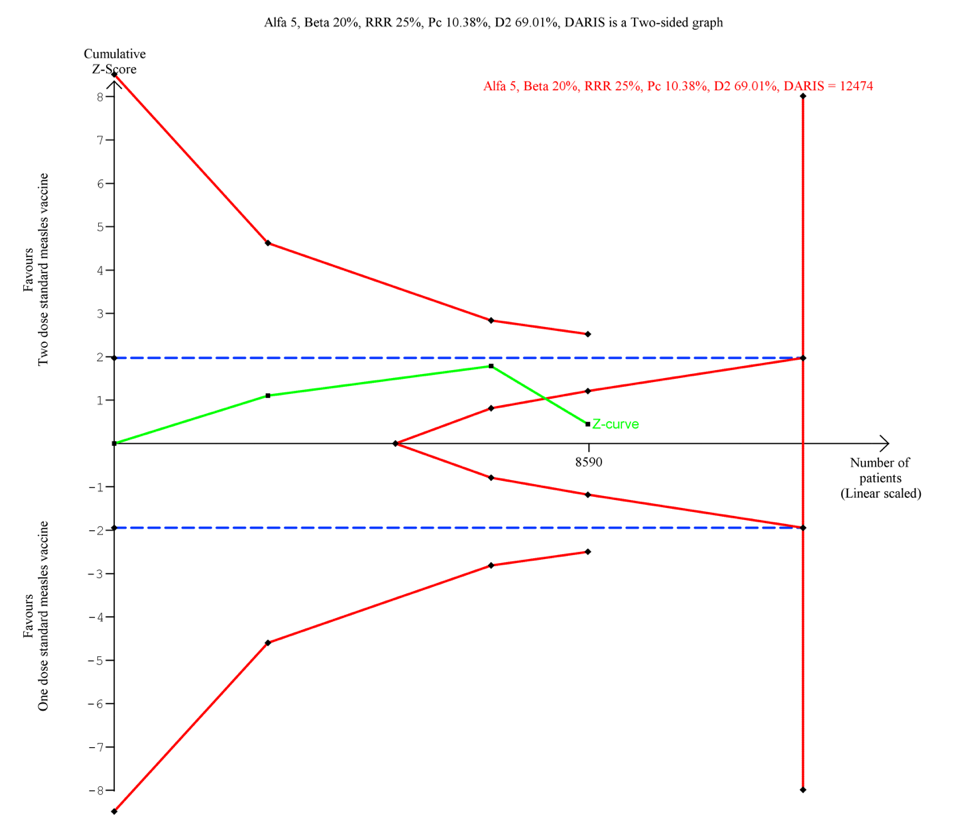
**

Figure A:

Pooled Effect, RR = 0.95 (0.77 to 1.18) p-value: 0.67

Heterogeneity, Q = 5.37 Heterogeneity, Q, p-value = 0.07

Inconsistency, I² = 0.63 Diversity, D² = 0.69

This TSA analysis was made for female sex only. The pooled effect was RR = 0.95 with p = 0.67. The z-curve reaches the area of futility. Thus, no significant difference is found between a two-dose measles vaccine program and a one-dose measles vaccine program when investigating a 25% relative risk reduction level in morbidity.

**Figure B: Morbidity effects of a two- versus one-dose program of standard titre measles vaccine on male sex only. Risk ratios (RR) with 95% confidence intervals.**


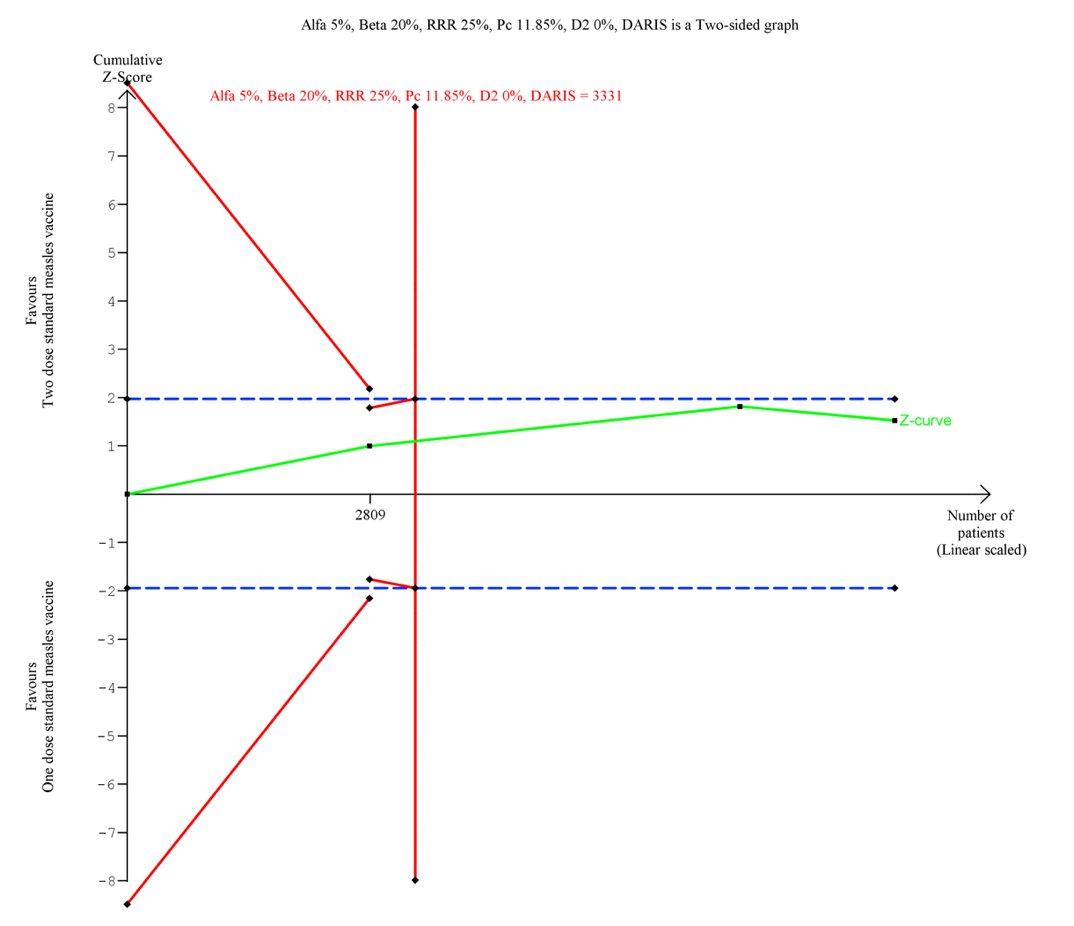


Figure B:

Pooled Effect, RR = 0.92 (0.83 to 1.03) p-value: 0.13

Heterogeneity, Q = 1.26 Heterogeneity, Q, p-value = 0.53

Inconsistency, I² = 0.00 Diversity, D² = 0.00

This TSA analysis was made for male sex only. The pooled effect was RR = 0.92 with p= 0.13. The z-curve reaches the area of futility. Thus, no significant difference is found between a two-dose measles vaccine program and a one-dose measles vaccine program when investigating a 25% relative risk reduction level in morbidity.
