# Supplementary material for: Measles vaccines and non-specific effects on mortality or morbidity: A systematic review and meta-analysis
Source: PLoS One. 2025 Jul 2;20(7):e0321982. doi: 10.1371/journal.pone.0321982 (PMC12221017; doi:10.1371/journal.pone.0321982)
Supplement: S6 appendix — (DOCX) [file pone.0321982.s016.docx]

## **S6 appendix. The high titre measles vaccine**

Eight trials or re-analyses of trials investigating HTMV were identified. Two re-analyses reported no significant differences in overall mortality, or mortality sub-analyses stratified by sex during follow-up periods of 6-36 and 18-60 months, respectively[9, 10]. Two other re-analyses found no differences in mortality after five years of follow-up. The sex-specific effects were reported as ambiguous[31, 32]. Another trial compared the Edmonston-Zagreb High-titre (EZ-HT), the high-titre Schwarz measles vaccine (SW-HT), and the medium titre Schwarz measles vaccine (SW-MT) given to 5-month-old infants[11]. Three articles based on this trial were found: First, an interim analysis reported significantly higher child mortality for the HTMVs and no sex difference. In the final analysis, no significant difference in overall mortality but significantly higher mortality for females were reported. Finally, after five years of follow-up, no significant overall or sex difference in mortality was reported[11, 33, 34].

A re-analysis of four RCTs from the 1980’s and 1990 of infants randomised to inactivated polio vaccine after MCV, found a significantly higher mortality rate in females compared to males. In infants without a third dose of the diphtheria, tetanus, and pertussis vaccine (DTP), no significant sex difference was reported[35].

**Figure A: Mortality effects of standard titre measles vaccine compared to high titre measles vaccine (< 4.7 log_10_ infectious units per dose). Risk ratios (RR) with 95% confidence intervals.**


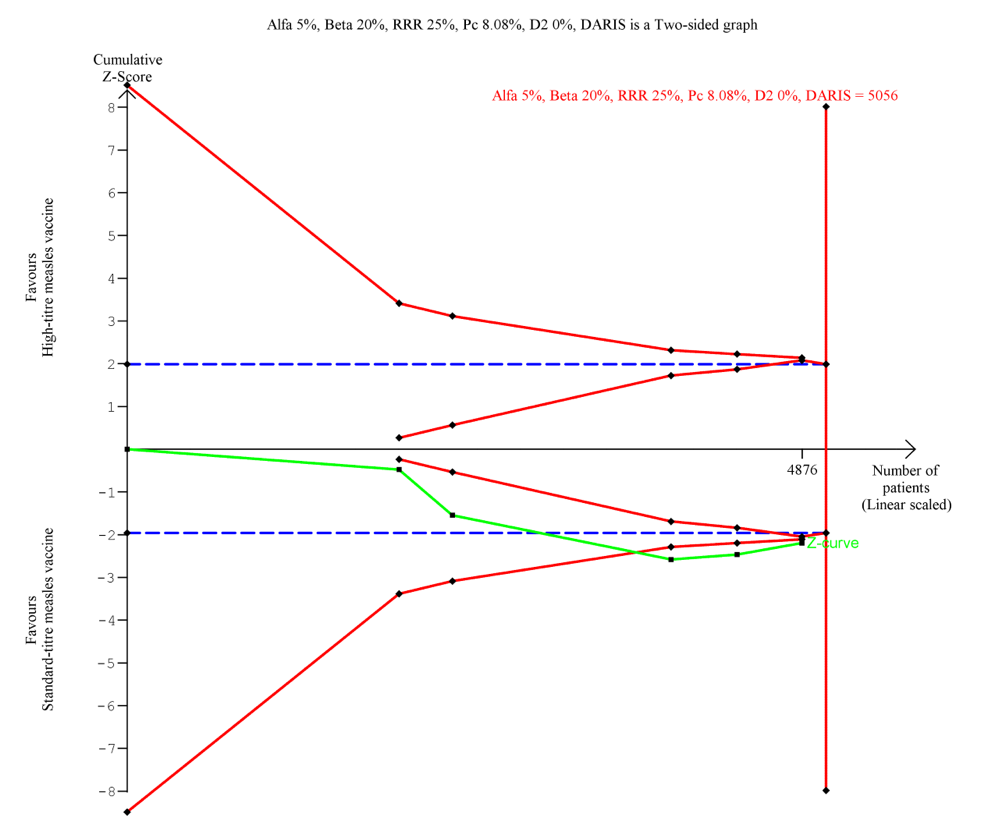


Figure A:

Pooled Effect, RR = 1.22 (1.02 to 1.46) p-value: 0.03

Heterogeneity, Q = 3.16 Heterogeneity, Q, p-value = 0.53

Inconsistency (I²): 0.00 Diversity, D² = 0.00

This TSA analysis has a pooled effect of RR = 1.22 with p = 0.03. The z-curve reaches the boundary of harm. Thus, a conclusive and significant difference was found between the high titre and the standard titre measles vaccine. There was a lower risk of mortality associated with the standard titre measles vaccine.

*
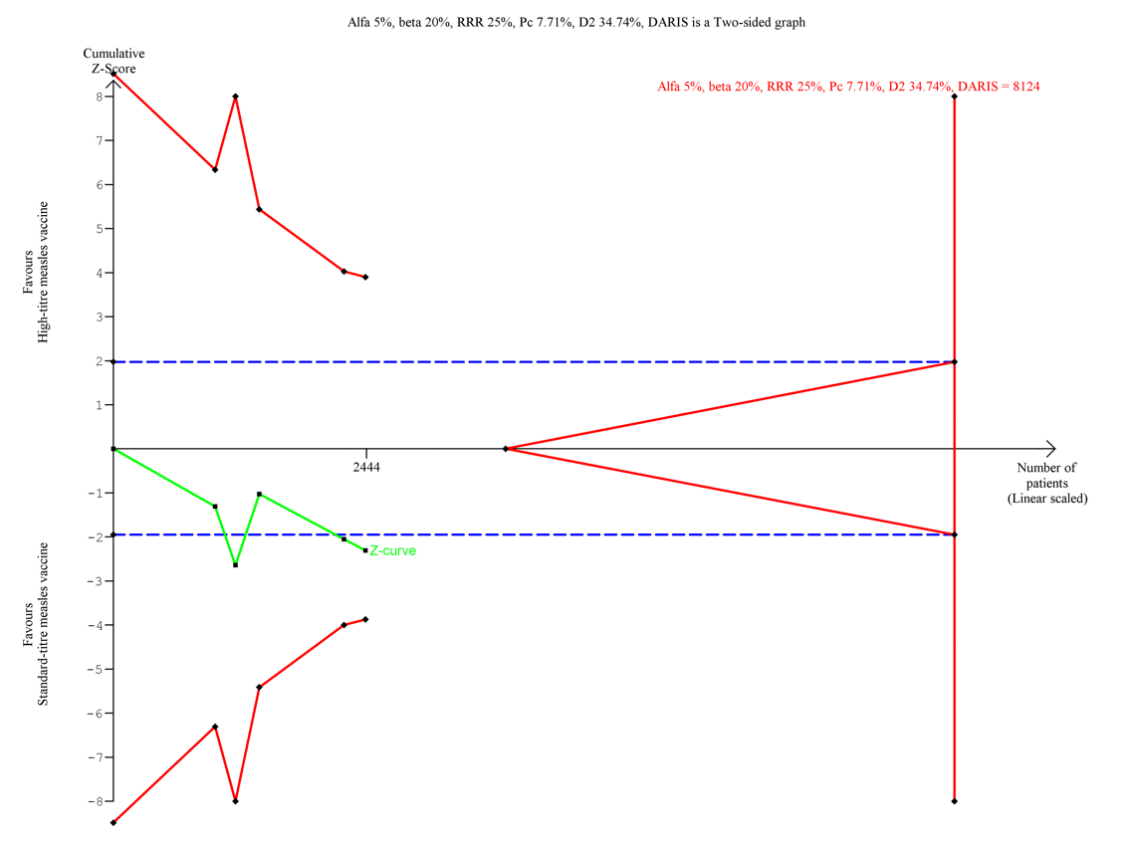
***Figure B: Mortality effects of standard titre measles vaccine compared to high titre measles vaccine for girls only (< 4.7 log_10_ infectious units per dose). RRR = 25%.**

**Figure C: Mortality effects of standard titre measles vaccine compared to high titre measles vaccine for girls only (< 4.7 log_10_ infectious units per dose), RRR = 33%.**


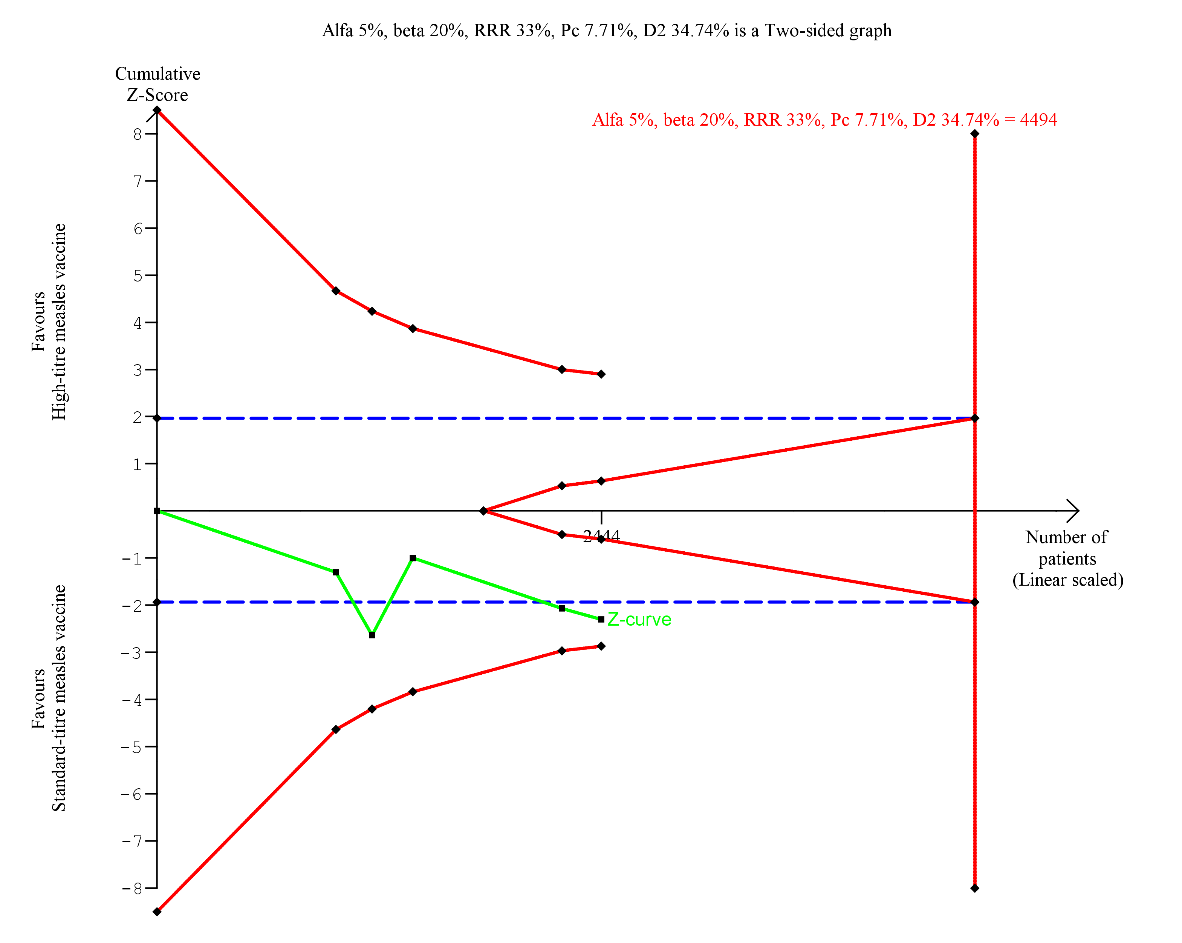


Figure B&C:

Pooled Effect, RR = 1.45 (1.06 to 1.99) p-value: 0.02

Heterogeneity, Q = 5.62 Heterogeneity, Q, p-value = 0.23

Inconsistency, I² = 0.29 Diversity, D² = 0.35

In this meta-analysis for girls only a statistically significant difference was found with a RR = 1.45 and a p = 0.02. This result favoured the standard titre measles vaccine, but the difference was not conclusive either on a RRR = 25% or a RRR = 33%
