# Supplementary material for: Measles vaccines and non-specific effects on mortality or morbidity: A systematic review and meta-analysis
Source: PLoS One. 2025 Jul 2;20(7):e0321982. doi: 10.1371/journal.pone.0321982 (PMC12221017; doi:10.1371/journal.pone.0321982)
Supplement: S7 Table — (DOCX) [file pone.0321982.s017.docx]

## **Table S7: GRADE**

| Quality assessment | | | | | | | N  participants | Pooled  effect | Quality  grading | Importance |
| --- | --- | --- | --- | --- | --- | --- | --- | --- | --- | --- |
| N  trials | Trial design | Risk of bias | Inconsistency | Indirectness | Imprecision | Other | Intervention/Control | RR (95% CI) |  |  |
| **MORTALITY (Standard titre: ‘two dose vs one dose’ or ’MV for all vs restrictive policy’)** | | | | | | | | | | |
| 7^1^ | RCT/reanalysis/sub-study | Moderate^A^ | I^2^=10% Low^F^ | High^B^ | Moderate^C^ | 1: Overlapping data  2: Publications bias^D^ | I: 17,531^E^ C: 17,985 | 0.87 (0.76- 0.99) | Low | Very important |
| 3^2^ | Original RCTs | Moderate-high^A^ | I^2^=60% Moderate | Moderate^L^ | Moderate^C^ | None | I: 10,679  C: 10,645 | 1.02 (0.75-1.37) | Moderate-high | Very important |
| **MORBIDITY (Standard titre: ‘two dose vs one dose’ or ’MV for all vs restrictive policy‘ or ’MV to intervention group vs prior vaccines’)** | | | | | | | | | | |
| 9^3^ | RCT/reanalysi/sub-study | High^A^ | I^2^=10% Low ^F^ | Moderate^G^ | Moderate^H^ | 1: Overlapping data  2: Publication bias^D^ | I: 21,013^E^ C: 24,202 | 1.04(0.97-1.11)^I^ | Low | Very important |
| **MORTALITY (High titre vs standard titre)** | | | | | | | | | | |
| 8^4^ | RCT/reanalysis/sub-study | High^A^ | I^2^=49% Moderate | Low | Moderate^J^ | 1: Overlapping data  2: Publication bias^D^ | I: 9093^E^ C: 7621 | 1.24 (1.10-1.39) | Moderate | Limited importance^K^ |
| CI: 95% Confidence intervals; RR: Risk ratio; RCT: randomised controlled trial; N: Number of; N/A: non-applicable; OIS: Optimal information size **Explanations**   1. Bias assessments available in Suppl. Table S1. Lack of blinding of infants’ mothers was present in 20/23 trials which led to a high risk of bias for the soft outcome morbidity. For the hard outcome mortality lack of blinding had a smaller impact on the overall risk of bias. 2. Low-income setting. Interventions differ largely between trials. Use of composite outcomes including mortality and morbidity. 3. The 95% CI includes the potential for both harm as well as benefit in all trials OIS criterion is met. 4. Publication bias not determined since less than 10 trials were included for each outcome[3, 11] 5. Individuals are overlapping between re-analyses of trials and sub-trials. 6. One trial[12] did not mention absolute number of events and was excluded from this calculation. 7. Low-income setting. Interventions differ largely between trials. Outcome measurement and definition of morbidity differ. 8. The 95% CI includes the potential for both harm as well as benefit in seven of nine trials. One was excluded from analyses. OIS criterion is met. 9. One study[13] was excluded from the pooled effect calculations as the events in both control and intervention groups were composed of different outcomes and therefore exceeded the number of individuals. 10. The 95% CI includes the potential for both harm as well as benefit in six of eight trials. OIS criterion is met. 11. Please note that the high titre measles vaccine was discontinued in 1992. 12. 50% of the study population in one trial received a non-intended intervention during follow-up.   **References**   1. Aaby et al 2010[9], Aaby et al 2014[14], Fisker et al 2018[15], Schoeps et al 2018[16], Nielsen et al 2022[17], Berendsen et al 2022[12], Byberg et al 2021[18] 2. Aaby et al 2010[9], Fisker et al 2018[15], Nielsen et al 2022[17] 3. Martins et al 2014[19], Brønd et al 2018[20], Schoeps et al 2018[16], Steiniche et al 2020[21], Varma et al 2020[22], Byberg et al 2021[18], Berendsen et al 2022[12], Zimakoff et al 2023[23], Do et al 2017[13] 4. Aaby et al 2007[24], Aaby et al 1994[25], Aaby et al 1993[26], Aaby et al 1996[27], Aaby et al 1994[28], Libman et al 2002[29], Garenne et al 1991[30], Holt et al 1993[31] | | | | | | | | | | |
